# Gastric Cancer Subtypes in Tumour and Nontumour Tissues by Immunologic and Hallmark Gene Sets

**DOI:** 10.1155/2022/7887711

**Published:** 2022-08-27

**Authors:** Xia Zheng, Yanzhong Wang, Yirui Chen

**Affiliations:** ^1^Department of Gastroenterology, Sir Run Run Shaw Hospital, Zhejiang University School of Medicine, 3 East Qingchun Road, Hangzhou, Zhejiang 310016, China; ^2^Department of Clinical Laboratory, Sir Run Run Shaw Hospital, Zhejiang University School of Medicine, 3 East Qingchun Road, Hangzhou, Zhejiang 310016, China; ^3^Cancer Center, Department of Hematology, Zhejiang Provincial People's Hospital, Affiliated People's Hospital Hangzhou Medical College, 58 Shangtang Road, Hangzhou, Zhejiang 310014, China

## Abstract

A previous research study on differentiating gastric cancer (GC) into distinct subtypes or prognostic models was mostly based on GC tissues, which neglected the role of nontumour tissues in GC subtypes. The purpose of the research was to identify GC subtypes on the basis of tumour and adjacent nontumour tissues to assess the prognosis of GC patients. We characterized three GC subtypes on the basis of the immunologic and hallmark gene sets in GC and adjacent nontumour tissues: among them, the GC patients with subtype I had the longest survival time compared to patients with other subtypes. The classification was closely associated with *T* stage and pathological stage of GC patients. A prognostic model containing two gene sets was constructed by LASSO analysis. Kaplan–Meier analysis showed that patients in the high-risk group survived longer than those in the low-risk group and the two prognostic genes sets in the model were strongly correlated with survival status. Then, GO and KEGG analyses and PPI network show that nontumour and tumour tissues are influencing the prognosis of GC patients in separate manners. In summary, we emphasized the prognostic value of nontumour tissue in GC patients and proposed a novel insight that both changes in tumour and nontumour tissues should be taken into account when selecting a treatment strategy for GC.

## 1. Introduction

Gastric cancer (GC) is the second commonest cause of tumour death globally, with an estimated annual mortality rate of over 720,000 cases [1]. Although treatment schemes for GC have been greatly advanced, including systemic chemotherapy, radiation therapy, surgery, immunotherapy, and targeted therapy, the five-year survival rate for GC patients still has not improved significantly [2, 3]. Moreover, even in patients with similar clinical characteristics, the prognosis is completely different after almost the same treatment regimen [4–6]. Consequently, because of the highly invasive mortality and adverse prognosis of GC, recognition of GC subtypes and prognostic gene sets can improve patient prognosis through individualized drug therapy and precise assessment of prognosis.

Previous research on differentiating GC into distinct subtypes or prognostic models was mostly based on GC tissues, which neglected the role of nontumour tissues in GC subtypes. Distinct subtypes have been established on the basis of the integration of genes and transcriptional expression profiles, genetic polyomics genes, and metabolic genes in GC [7–10]. Moreover, research on changes in GC pathway activity tended to focus on single pathways or class of molecules instead of systematically analyzing various pathways in tumour and nontumour samples [11–13]. Nevertheless, these studies were based on the tumour tissue itself, and the significant effect of nontumour tissue on the prognosis of the tumour was completely ignored.

First of all, after surgical removal of GC patients, the residual GC cells might still exist in the neighboring nontumour tissues, which may lead to the recurrence of GC. Moreover, the tumour cells scattered in blood are likely to pass through the blood vessels and fix again in the residual stomach, leading to the recurrence of GC. All these circumstances indicate that immune and molecular changes in nontumour tissues have a great influence in the prognosis of GC patients [14, 15].

This study provides different novel perspectives to precisely assess the prognosis of GC patients—the immunologic and hallmark gene sets in tumour and adjacent nontumour tissues, suggesting that variations in tumour and nontumour tissues should be taken into account when making a decision about GC treatment approaches.

## 2. Materials and Methods

### 2.1. Data Preparation

The RNA-seq data for tumour and nontumour tissues and accompanying clinicopathological information (TCGA and GSE84437) were obtained from The Cancer Genome Atlas (TCGA: https://portal.gdc.cancer.gov) and Gene Expression Omnibus (GEO: https://www.ncbi.nlm.nih.gov/geo) databases [16]. A collection of 4922 immunologic and hallmark gene sets were downloaded from the Gene Set Enrichment Analysis (GSEA: https://www.gsea-msigdb.org/gsea/index.jsp) [17]. The 318 cases (TCGA database) and 433 cases (GEO database) were used for using in model construction and OS analysis.

### 2.2. Gastric Cancer Subtypes

We applied gene set variance analysis (GSVA) to evaluate the relative enrichment of gene sets in the sample population. Then, we selected the features using Cox regression analysis and divided samples into different groups by the nonnegative matrix factorization (NMF) method. Another expression profile dataset with a distinct platform was employed to verify our classification. Next, we evaluated the association between GC subtypes and clinical features using a chi-square test. Finally, we computed the differentiated enrichment scores of gene sets among the two subtypes, crossed them, and filtered them by the Cox analysis (*p* < 0.05).

### 2.3. Prognostic Gene Sets Model

Prognostic gene sets were identified and a prognostic risk assessment model was developed employing the least absolute shrinkage and selection operator (LASSO) analysis. We used the median risk score to divide samples into higher and lower groups. The Kaplan–Meier analysis was applied to demonstrate survival differences between high-risk and low-risk groups, and survival curves were mapped in the model for each prognostic gene set.

### 2.4. Functional Enrichment

To elucidate the mechanism of prognostic gene sets, we retrieved the genes included in each gene set and conducted gene ontology (GO) and Kyoto Encyclopedia of Genes and Genomes (KEGG) enrichment analyses in tumour and nontumour tissues, separately.

### 2.5. Protein-Protein Interaction

The genes in the N and *T* gene sets were placed in STRING (https://stringdb.org), respectively, and the interaction score of the protein-protein interaction (PPI) network was greater than 0.15 to obtain the data of gene interactions. The PPI network data were loaded into Cytoscape v3.9.1, and the MCODE inserter was applied to identify the first two clusters and the hub genes in each cluster.

## 3. Results

### 3.1. Identification of GC Different Subtypes

**Figure 1:**
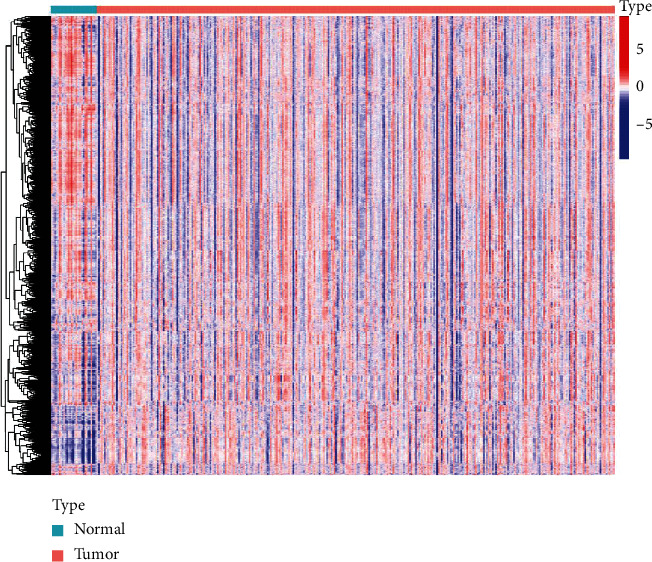
Heat map of enrichment scores from immunologic and hallmark gene sets. N, normal; T, tumour.

**Figure 2:**
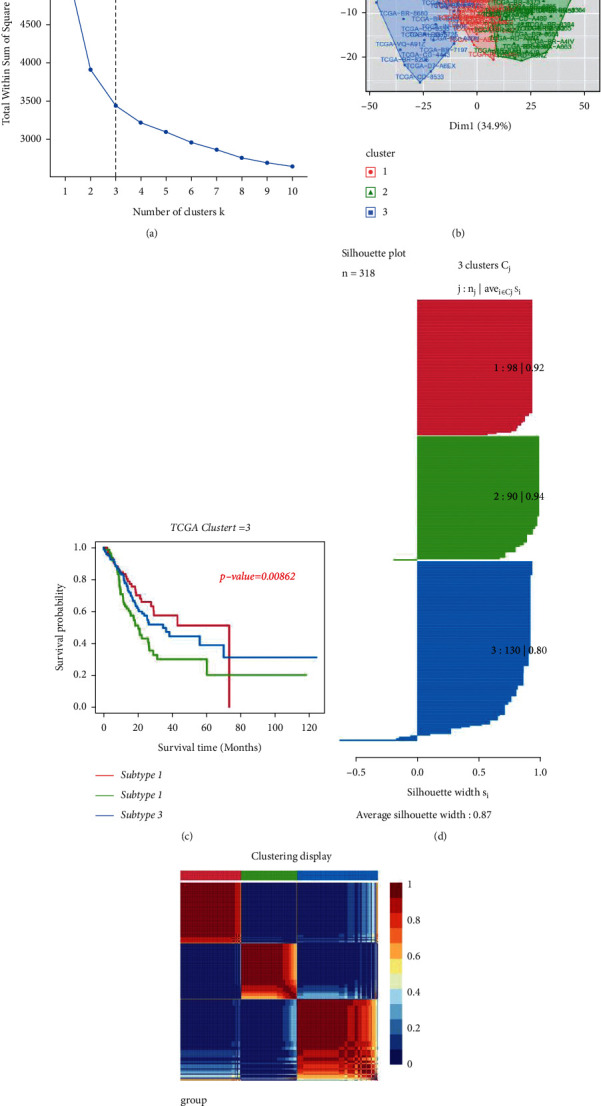
Identification of GC subtypes from TCGA. (a) Optimal number of clusters. (b) Visualization of cluster results. (c) NMF clustering results from GC samples. (d) Silhouette width plots with a value of 0.87. (e) Kaplan–Meier survival analysis.

**Figure 3:**
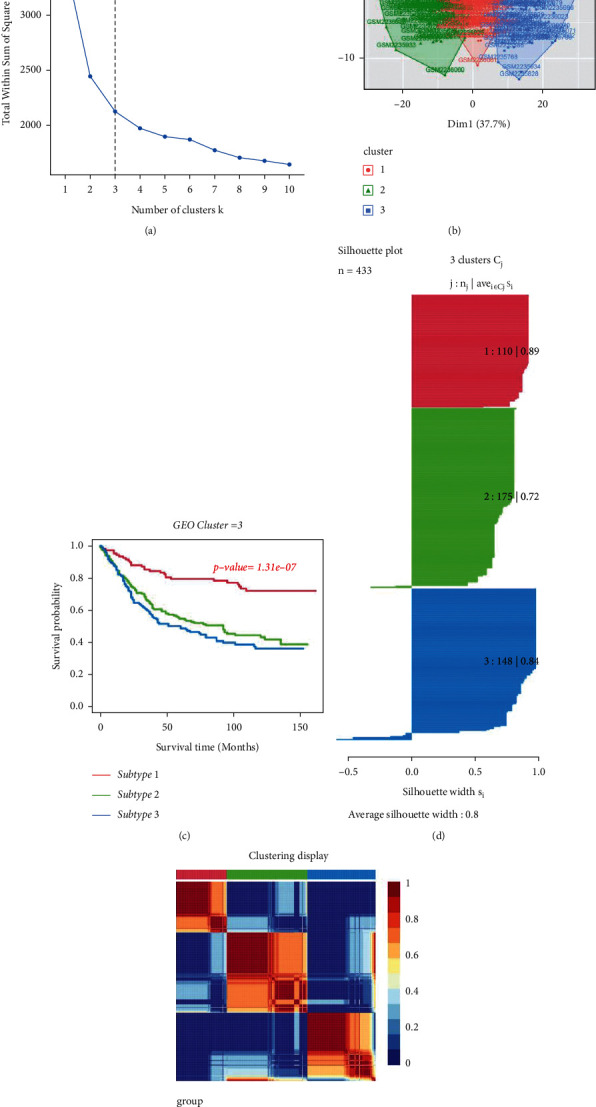
Identification of GC subtypes from GSE84437. (a) Optimal number of clusters. (b) Visualization of cluster results. (c) NMF clustering results from GC samples. (d) Silhouette width plots with a value of 0.80. (e) Kaplan–Meier survival analysis.

We counted the enrichment scores of 4922 gene sets by GSVA on the basis of TCGA in order to elucidate the integrative landscape of changes in immunologic and hallmark gene sets in GC and nontumour tissues (Figure 1). On the basis of the results of NMF, we classified the GC patients into 3 distinct subtypes with a silhouette width value of 0.87, and the GC patients with subtype 1 had longer overall survival compared to types 2 and 3 (*p* < 0.01; Figures 2(a)–2(e)). In the validation set, we also classified GC patients into 3 different subtypes with a silhouette width value of 0.80, and the GC patients with subtype I had the longest survival time compared to patients with other subtypes (*p* < 0.001; Figures 3(a)–3(e)). The clinical features of each subtype in TCGA are given in Supplementary Table 1. Overall, the classification was rational and valid for predicting the prognosis of GC patients from diverse datasets.

### 3.2. Assessment of GC Distinct Subtypes

**Figure 4:**
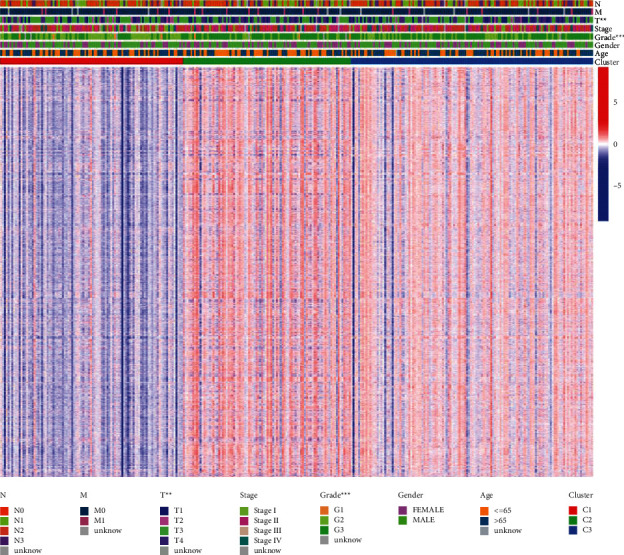
Assessment of the association between clinical features and GC subtypes.

**Figure 5:**
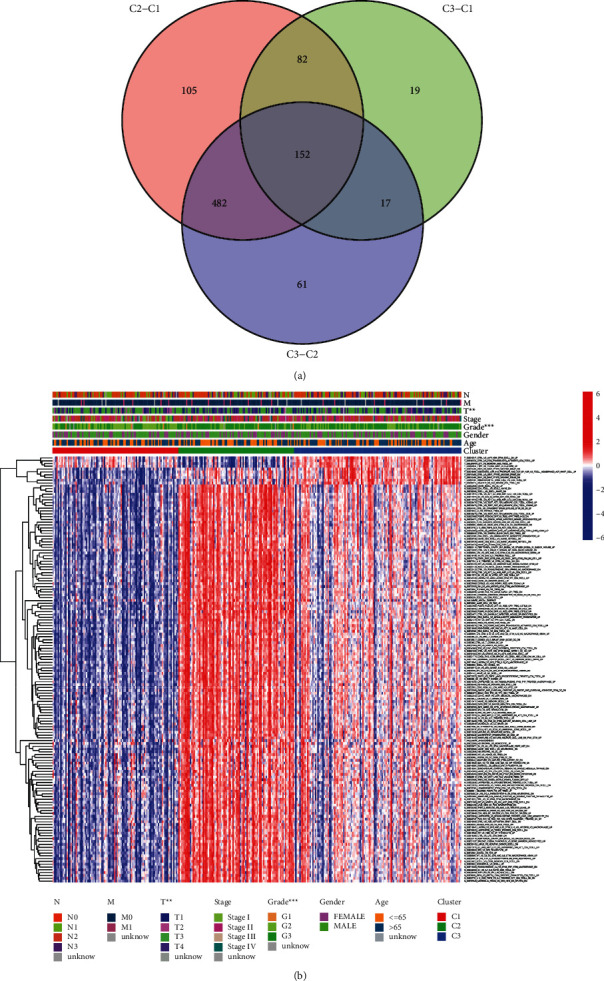
Characterization of prognostic gene sets from GC subtypes. (a) 152 differentially expressed gene sets identified. (b) The expression profile of the 19 prognosis-related gene sets in each sample and the relationship with clinical characteristics.

To explore the relationship between the classifications with clinical characteristics, a chi-square test indicated that the classification was closely related to *T* stage and pathological stage of GC patients (Figure 4). To recognize prognosis-related gene sets shared by different subtypes, we identified differentiated gene sets between each of the two subgroups, crossed them (Figure 5(a)), and further filtered for prognosis-related gene sets using univariate Cox analysis. Through the above steps, we finally acquired 152 differentially expressed gene sets (immunologic and hallmark gene set score from GSVA) and 19 prognosis-related gene sets existing in different subgroups.  Figure 5(b) shows the expression profile of the 19 prognosis-related gene sets in each sample and the relationship with clinical characteristics.

### 3.3. Construction of the Prognostic Risk Model

**Figure 6:**
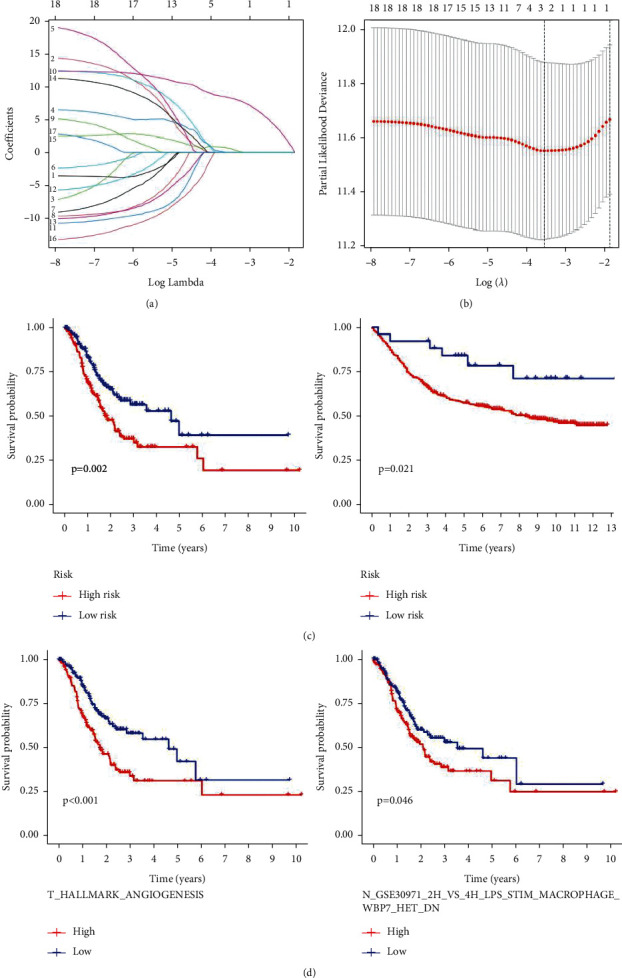
Construction and verification of a prognostic model. (a) Profiles of LASSO coefficients for 2 prognostic gene sets. (b) Coefficient profile plot generated against the log sequence. (c)-(d) Kaplan–Meier analysis showing that patients in the high-risk group survived longer than those in the low-risk group (TCGA and GSE84437). (e) Kaplan–Meier survival curves of 2 prognostic gene sets.

A prognostic model containing two gene sets was constructed by the LASSO analysis (Figures 6(a) and 6(b)). Among these gene sets, one was in nontumour tissues (N gene sets: N_GSE30971_2H_VS_4H_LPS_STIM_MACROPHAGE_WBP7_HET_DN) and one was in tumour tissues (T gene sets: T_HALLMARK_ANGIOGENESIS). The Kaplan–Meier analysis showed that patients in the high-risk group survived longer than those in the low-risk group (Figure 6(c)), and the two prognostic gene sets in the model were strongly correlated with survival status (Figure 6(d)).

### 3.4. Exploration of Functional Enrichment

**Figure 7:**
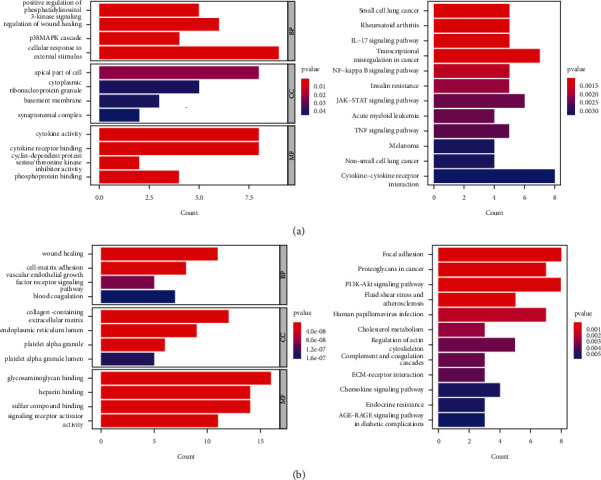
(a)-(b) The GO and KEGG enrichment analyses for N gene sets and T gene sets, separately.

To investigate the potential mechanisms of the 2 prognostic gene sets, we abstracted the genes contained in each gene set separately and performed GO and KEGG enrichment analyses in tumour and nontumour tissues. In nontumour tissues, the genes from N gene sets were primarily related to terms of cytokine activity, receptor activity, and various receptor binding by GO enrichment analysis and terms of different tumour-related pathways, inflammatory pathways, and immune-related disease by KEGG enrichment analysis, respectively (Figure 7(a)). In tumour tissues, the genes from *T* gene sets were primarily related to terms of cell adhesion, vascular growth, blood coagulation, and receptor activity and terms of focal adhesion, different tumour-related pathways, and metabolic disturbance by KEGG enrichment analysis (Figure 7(b)). The results of the GO and KEGG enrichment analyses from N and *T* gene sets are given in Supplementary Tables 2 and 3.

### 3.5. Construction of the Protein-Protein Interaction Network

**Figure 8:**
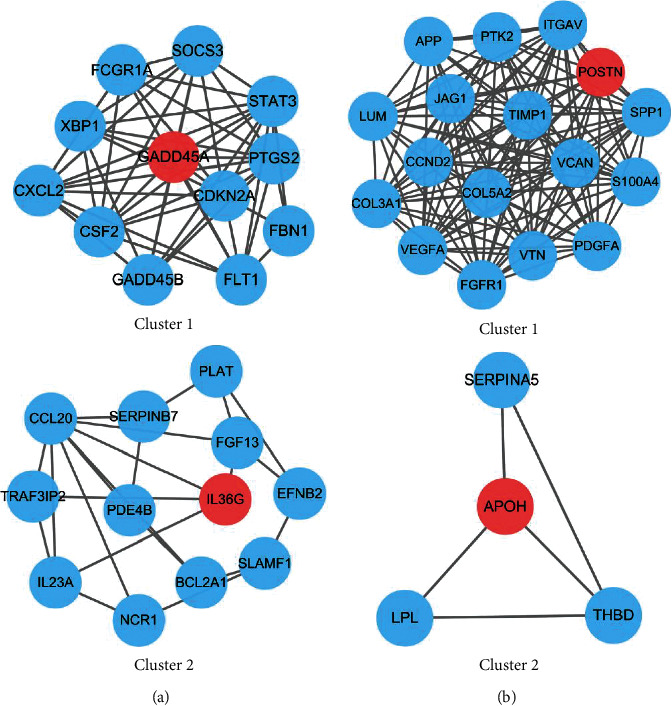
Establishment of protein-protein interaction networks. (a)-(b) The top 2 clusters and hub genes acquired by the MCODE.

We structured PPI networks for N and T gene sets from STRING (Supplementary Figure S1 and Supplementary Figure S2) and further screened hub genes by the MCODE plug-in in Cytoscape. For N gene sets, the hub gene from cluster 1 was GADD45A, mainly related to growth arrest and DNA damage, and cluster 2 was IL-36G, closely linked to immune and inflammatory response (Figure 8(a)). For T gene sets, the hub gene from cluster 1 was POSTN, mainly related to adhesion and migration, and cluster 2 was APOH, closely linked to metabolic disturbance (Figure 8(b)).

## 4. Discussion

Previous studies on GC subtype classification or prognostic models have been almost exclusively based on GC tissue itself, which ignores the role of adjacent nontumour tissues in GC. Li et al. revealed three GC subtypes on the basis of the immunogenomic profiles and distinguished different molecular characteristics at the genetic, transcriptomic, and epigenetic levels [18]. Zhang et al. identified three distinct advanced GC subtypes by immune signatures, which can predict the prognosis of advanced GC [19]. Zhu et al. recognized four GC metabolic subtypes and patients with cholesterogenic have better prognosis [20]. In the study, we offer a complete perspective of the changes of immunologic and hallmark gene sets in GC and neighboring nontumour tissues, which contributes to advancing the understanding of the prognostic role of nontumour tissues and emphasizes that greater attention should be paid to the changes of both nontumour and tumour tissues, not just tumour tissue itself.

Local recurrence of GC and distant metastasis to other organs are the major reasons for death of GC patients [21]. For one thing, after a patient with GC undergoes complete surgical treatment, GC cells may lurk in the adjacent nontumour tissues, leading to local recurrence of GC. For another thing, potential GC cells distributed in blood can reenter and colonize gastric tissues through blood circulation. Consequently, molecular changes in the neighboring nontumour tissues should be taken into account when choosing the treatment for GC and not only the tumour tissues. In the study, we characterized three GC subtypes on the basis of the immunologic and hallmark gene sets in GC and adjacent nontumour tissues; among them, the GC patients with subtype I had the longest survival time compared to patients with other subtypes.

The two prognosis-related gene sets were utilized to construct the model, one in nontumour tissues (N gene sets: N_GSE30971_2H_VS_4H_LPS_STIM_MACROPHAGE_WBP7_HET_DN), and one in tumour tissues (T gene sets: T_HALLMARK_ANGIOGENESIS). In nontumour tissues, the genes from N gene sets were primarily related to terms of different tumour-related pathways, inflammatory pathways, and immune-related disease by the KEGG enrichment analysis. A study showed that IL-17 promotes epithelial-mesenchymaltransition-like transformation of GC cells and demonstrated that STAT3 is its downstream signaling molecule [22]. A meta-analysis indicated that single nucleotide polymorphisms in IL-17 were significantly related to the risk of GC [23]. In tumour tissues, the genes from T gene sets were primarily related to terms of focal adhesion, different tumour-related pathways, and metabolic disturbance by the KEGG enrichment analysis, respectively.

This study shows that tumour and nontumour tissues influenced the prognosis of GC patients in separate manners. In nontumour tissues, the genes were primarily related to terms of cytokine activity, receptor activity, and various receptor bindings by GO enrichment analysis. In tumour tissues, the genes were primarily related to terms of cell adhesion, vascular growth, blood coagulation, and receptor activity. The hub genes in nontumour tissues are GADD45A and IL-36G, while the hub genes in tumour tissues are POSTN and APOH. GADD45A expression levels have been correlated with response and overall survival after neoadjuvant chemotherapy for GC [23]. A study demonstrated that POSTN is not only a risk factor for the development of GC but also promotes metastasis [25]. IL-36G and APOH have also been associated with different types of tumour development and progression [26–29].

Our study has several limitations. On the one hand, the number of tumour samples differed too much from nontumour samples. On the other hand, the prognostic gene set derived from the study was not validated by in vitro, in vivo, or clinical sample experiments. In future work, we will further expand the sample size to include more nontumour tissues and use various experiments to follow-up and validate our results, which is a very meaningful work. Moreover, it would be interesting to know whether the immune and hallmark-related genes were from intramural vs. transitional vs. peripheral tumour areas as they might have different immune gene signatures. Unfortunately, such information is not available as the RNA-seq data were produced from the entire tissue. Future studies with spatial transcriptomics based on single-cell RNA sequencing will answer such critical questions.

## 5. Conclusions

In summary, we emphasized the prognostic value of the nontumour tissue in GC patients and propose the conception that both changes in tumour and nontumour tissues should be taken into account when selecting a treatment strategy for GC.

## Data Availability

The data used to support this study are available at the TCGA (https://portal.gdc.cancer.gov/) and GEO (https://www.ncbi.nlm.nih.gov/geo/) databases.

## Supplementary Materials

Supplementary Materials Supplementary Table S1. The association between clinical features and subtypes. Supplementary Table S2. Detailed information on GO and KEGG enrichment analyses of N gene sets. Supplementary Table S3. Detailed information on GO and KEGG enrichment analyses of T gene sets. Figure S1. The protein-protein interaction network for N gene sets. Figure S2. The protein-protein interaction network for T gene sets.
