# Supplementary material for: Gastric Cancer Subtypes in Tumour and Nontumour Tissues by Immunologic and Hallmark Gene Sets
Source: J Oncol. 2022 Aug 27;2022:7887711. doi: 10.1155/2022/7887711 (PMC9440817; doi:10.1155/2022/7887711)
Supplement: Supplementary Materials — Supplementary Table S1. The association between clinical features and subtypes. Supplementary Table S2. Detailed information on GO and KEGG enrichment analyses of N gene sets. Supplementary Table S3. Detailed information on GO and KEGG enrichment analyses of T gene sets. Figure S1. The protein-protein interaction network for N gene sets. Figure S2. The protein-protein interaction network for T gene sets. [file 7887711.f1.zip › TableS1.docx]

**Supplementary TABLE S1 |** The association between clinical features and subtypes.

| Covariates | Cluster | Total | C1 | C2 | C3 | Pvalue |
| --- | --- | --- | --- | --- | --- | --- |
| Age | <=65 | 144(45.28%) | 42(42.86%) | 48(53.33%) | 54(41.54%) | 0.2027 |
| Age | >65 | 173(54.4%) | 56(57.14%) | 42(46.67%) | 75(57.69%) |  |
| Age | unknow | 1(0.31%) | 0(0%) | 0(0%) | 1(0.77%) |  |
| Gender | FEMALE | 117(36.79%) | 34(34.69%) | 37(41.11%) | 46(35.38%) | 0.601 |
| Gender | MALE | 201(63.21%) | 64(65.31%) | 53(58.89%) | 84(64.62%) |  |
| Grade | G1 | 7(2.2%) | 4(4.08%) | 3(3.33%) | 0(0%) | 0 |
| Grade | G2 | 116(36.48%) | 53(54.08%) | 19(21.11%) | 44(33.85%) |  |
| Grade | G3 | 188(59.12%) | 38(38.78%) | 65(72.22%) | 85(65.38%) |  |
| Grade | unknow | 7(2.2%) | 3(3.06%) | 3(3.33%) | 1(0.77%) |  |
| Stage | Stage I | 43(13.52%) | 22(22.45%) | 7(7.78%) | 14(10.77%) | 0.082 |
| Stage | Stage II | 101(31.76%) | 26(26.53%) | 30(33.33%) | 45(34.62%) |  |
| Stage | Stage III | 130(40.88%) | 37(37.76%) | 37(41.11%) | 56(43.08%) |  |
| Stage | Stage IV | 31(9.75%) | 8(8.16%) | 11(12.22%) | 12(9.23%) |  |
| Stage | unknow | 13(4.09%) | 5(5.1%) | 5(5.56%) | 3(2.31%) |  |
| T | T1 | 16(5.03%) | 11(11.22%) | 1(1.11%) | 4(3.08%) | 0.0073 |
| T | T2 | 68(21.38%) | 18(18.37%) | 21(23.33%) | 29(22.31%) |  |
| T | T3 | 150(47.17%) | 53(54.08%) | 39(43.33%) | 58(44.62%) |  |
| T | T4 | 80(25.16%) | 16(16.33%) | 27(30%) | 37(28.46%) |  |
| T | unknow | 4(1.26%) | 0(0%) | 2(2.22%) | 2(1.54%) |  |
| M | M0 | 286(89.94%) | 88(89.8%) | 80(88.89%) | 118(90.77%) | 0.5969 |
| M | M1 | 21(6.6%) | 6(6.12%) | 8(8.89%) | 7(5.38%) |  |
| M | unknow | 11(3.46%) | 4(4.08%) | 2(2.22%) | 5(3.85%) |  |
| N | N0 | 94(29.56%) | 34(34.69%) | 21(23.33%) | 39(30%) | 0.3979 |
| N | N1 | 85(26.73%) | 22(22.45%) | 29(32.22%) | 34(26.15%) |  |
| N | N2 | 68(21.38%) | 24(24.49%) | 19(21.11%) | 25(19.23%) |  |
| N | N3 | 62(19.5%) | 15(15.31%) | 17(18.89%) | 30(23.08%) |  |
| N | unknow | 9(2.83%) | 3(3.06%) | 4(4.44%) | 2(1.54%) |  |
