# Supplementary material for: Gastric Cancer Subtypes in Tumour and Nontumour Tissues by Immunologic and Hallmark Gene Sets
Source: J Oncol. 2022 Aug 27;2022:7887711. doi: 10.1155/2022/7887711 (PMC9440817; doi:10.1155/2022/7887711)
Supplement: Supplementary Materials — Supplementary Table S1. The association between clinical features and subtypes. Supplementary Table S2. Detailed information on GO and KEGG enrichment analyses of N gene sets. Supplementary Table S3. Detailed information on GO and KEGG enrichment analyses of T gene sets. Figure S1. The protein-protein interaction network for N gene sets. Figure S2. The protein-protein interaction network for T gene sets. [file 7887711.f1.zip › TableS2.docx]

**Supplementary TABLE S2 |** Detailed information on GO and KEGG enrichment analysis of N gene sets.

| ONTOLOGY | ID | Description | p.adjust | qvalue | geneID |
| --- | --- | --- | --- | --- | --- |
| BP | GO:0014068 | positive regulation of phosphatidylinositol 3-kinase signaling | 0.340041526 | 0.318039475 | NKX3-1/FLT1/NEDD4/MYDGF/PDGFRA |
| BP | GO:0061041 | regulation of wound healing | 0.340041526 | 0.318039475 | PLAT/KANK1/XBP1/FERMT2/PDGFRA/SMOC2 |
| BP | GO:0038066 | p38MAPK cascade | 0.340041526 | 0.318039475 | GADD45A/MAP3K4/MAP3K20/GADD45B |
| BP | GO:0071496 | cellular response to external stimulus | 0.340041526 | 0.318039475 | GADD45A/SH3GLB1/PTGS2/MTPN/XBP1/ELAPOR1/CDKN2B/STK26/PRKAA2 |
| BP | GO:0090303 | positive regulation of wound healing | 0.340041526 | 0.318039475 | KANK1/XBP1/FERMT2/SMOC2 |
| BP | GO:0072330 | monocarboxylic acid biosynthetic process | 0.340041526 | 0.318039475 | PTGS2/XBP1/RGN/ELOVL7/PNPLA8/PRKAA2/CYP7B1 |
| BP | GO:0006633 | fatty acid biosynthetic process | 0.340041526 | 0.318039475 | PTGS2/XBP1/RGN/ELOVL7/PNPLA8/PRKAA2 |
| BP | GO:0014066 | regulation of phosphatidylinositol 3-kinase signaling | 0.340041526 | 0.318039475 | NKX3-1/FLT1/NEDD4/MYDGF/PDGFRA |
| BP | GO:1900745 | positive regulation of p38MAPK cascade | 0.340041526 | 0.318039475 | GADD45A/MAP3K4/GADD45B |
| BP | GO:1903034 | regulation of response to wounding | 0.340041526 | 0.318039475 | PLAT/KANK1/XBP1/FERMT2/PDGFRA/SMOC2 |
| BP | GO:0007259 | receptor signaling pathway via JAK-STAT | 0.340041526 | 0.318039475 | GADD45A/SOCS3/CSF2/IFNA16/IL23A/STAT3 |
| BP | GO:1903036 | positive regulation of response to wounding | 0.340041526 | 0.318039475 | KANK1/XBP1/FERMT2/SMOC2 |
| BP | GO:0009750 | response to fructose | 0.340041526 | 0.318039475 | PTGS2/XBP1 |
| BP | GO:0045766 | positive regulation of angiogenesis | 0.340041526 | 0.318039475 | AGO2/FLT1/XBP1/STAT3/MYDGF/SMOC2 |
| BP | GO:0097696 | receptor signaling pathway via STAT | 0.340041526 | 0.318039475 | GADD45A/SOCS3/CSF2/IFNA16/IL23A/STAT3 |
| BP | GO:1904018 | positive regulation of vasculature development | 0.340041526 | 0.318039475 | AGO2/FLT1/XBP1/STAT3/MYDGF/SMOC2 |
| BP | GO:0060326 | cell chemotaxis | 0.340041526 | 0.318039475 | FLT1/PDE4B/CCL20/CXCL2/SLAMF1/PDGFRA/SMOC2/CYP7B1 |
| BP | GO:0050678 | regulation of epithelial cell proliferation | 0.340041526 | 0.318039475 | NKX3-1/FLT1/XBP1/CDKN2B/STAT3/RGN/MYDGF/MCC/CYP7B1 |
| BP | GO:0030048 | actin filament-based movement | 0.340041526 | 0.318039475 | PDE4B/EMP2/GJC1/FGF13/MYO5B |
| BP | GO:0001787 | natural killer cell proliferation | 0.340041526 | 0.318039475 | IL23A/SLAMF1 |
| BP | GO:2000786 | positive regulation of autophagosome assembly | 0.340041526 | 0.318039475 | SH3GLB1/ELAPOR1 |
| BP | GO:0008210 | estrogen metabolic process | 0.340041526 | 0.318039475 | PDGFRA/UGT2B15/HSD3B1 |
| BP | GO:0072538 | T-helper 17 type immune response | 0.340041526 | 0.318039475 | IL23A/STAT3/TRAF3IP2 |
| BP | GO:2001204 | regulation of osteoclast development | 0.374048511 | 0.349846071 | SIGLEC15/FBN1 |
| BP | GO:0044090 | positive regulation of vacuole organization | 0.374048511 | 0.349846071 | SH3GLB1/ELAPOR1 |
| BP | GO:0071801 | regulation of podosome assembly | 0.374048511 | 0.349846071 | CSF2/KIF9 |
| BP | GO:0030101 | natural killer cell activation | 0.374048511 | 0.349846071 | IFNA16/IL23A/SLAMF1/NCR1 |
| BP | GO:0061043 | regulation of vascular wound healing | 0.374048511 | 0.349846071 | XBP1/SMOC2 |
| BP | GO:0072540 | T-helper 17 cell lineage commitment | 0.374048511 | 0.349846071 | IL23A/STAT3 |
| BP | GO:0062013 | positive regulation of small molecule metabolic process | 0.374048511 | 0.349846071 | PTGS2/STAT3/RGN/EPM2AIP1/PRKAA2 |
| BP | GO:0045765 | regulation of angiogenesis | 0.374048511 | 0.349846071 | GADD45A/AGO2/FLT1/XBP1/EMP2/STAT3/MYDGF/SMOC2 |
| BP | GO:0014065 | phosphatidylinositol 3-kinase signaling | 0.374048511 | 0.349846071 | NKX3-1/FLT1/NEDD4/MYDGF/PDGFRA |
| BP | GO:0001754 | eye photoreceptor cell differentiation | 0.374048511 | 0.349846071 | RPGRIP1/OLFM3/STAT3 |
| BP | GO:1900744 | regulation of p38MAPK cascade | 0.374048511 | 0.349846071 | GADD45A/MAP3K4/GADD45B |
| BP | GO:1901342 | regulation of vasculature development | 0.374048511 | 0.349846071 | GADD45A/AGO2/FLT1/XBP1/EMP2/STAT3/MYDGF/SMOC2 |
| BP | GO:0023035 | CD40 signaling pathway | 0.374048511 | 0.349846071 | SLAMF1/TRAF3IP2 |
| BP | GO:0071380 | cellular response to prostaglandin E stimulus | 0.374048511 | 0.349846071 | GNG2/PRKAA2 |
| BP | GO:0042149 | cellular response to glucose starvation | 0.374048511 | 0.349846071 | SH3GLB1/XBP1/PRKAA2 |
| BP | GO:0038084 | vascular endothelial growth factor signaling pathway | 0.374048511 | 0.349846071 | FLT1/PDGFRA/SMOC2 |
| BP | GO:0031669 | cellular response to nutrient levels | 0.374048511 | 0.349846071 | SH3GLB1/XBP1/ELAPOR1/CDKN2B/STK26/PRKAA2 |
| BP | GO:0002827 | positive regulation of T-helper 1 type immune response | 0.374048511 | 0.349846071 | IL23A/SLAMF1 |
| BP | GO:0055089 | fatty acid homeostasis | 0.374048511 | 0.349846071 | XBP1/PRKAA2 |
| BP | GO:0070252 | actin-mediated cell contraction | 0.374048511 | 0.349846071 | PDE4B/EMP2/GJC1/FGF13 |
| BP | GO:0050673 | epithelial cell proliferation | 0.374048511 | 0.349846071 | NKX3-1/FLT1/XBP1/CDKN2B/STAT3/RGN/MYDGF/MCC/CYP7B1 |
| BP | GO:0006636 | unsaturated fatty acid biosynthetic process | 0.374048511 | 0.349846071 | PTGS2/ELOVL7/PNPLA8 |
| BP | GO:0009267 | cellular response to starvation | 0.374048511 | 0.349846071 | SH3GLB1/XBP1/ELAPOR1/STK26/PRKAA2 |
| BP | GO:0036035 | osteoclast development | 0.374048511 | 0.349846071 | SIGLEC15/FBN1 |
| BP | GO:0007566 | embryo implantation | 0.374048511 | 0.349846071 | PTGS2/AGO2/EMP2 |
| BP | GO:0002295 | T-helper cell lineage commitment | 0.374048511 | 0.349846071 | IL23A/STAT3 |
| BP | GO:0071800 | podosome assembly | 0.374048511 | 0.349846071 | CSF2/KIF9 |
| BP | GO:1901317 | regulation of flagellated sperm motility | 0.374048511 | 0.349846071 | TACR3/RGN |
| BP | GO:0046425 | regulation of receptor signaling pathway via JAK-STAT | 0.374048511 | 0.349846071 | GADD45A/SOCS3/IFNA16/IL23A |
| BP | GO:0050680 | negative regulation of epithelial cell proliferation | 0.374048511 | 0.349846071 | NKX3-1/FLT1/CDKN2B/RGN/MCC |
| BP | GO:0030595 | leukocyte chemotaxis | 0.374048511 | 0.349846071 | FLT1/PDE4B/CCL20/CXCL2/SLAMF1/CYP7B1 |
| BP | GO:2000278 | regulation of DNA biosynthetic process | 0.374048511 | 0.349846071 | MAP3K4/RGN/KCNK2/SMOC2 |
| BP | GO:0035313 | wound healing, spreading of epidermal cells | 0.374048511 | 0.349846071 | ARHGAP24/FERMT2 |
| BP | GO:0036498 | IRE1-mediated unfolded protein response | 0.374048511 | 0.349846071 | DNAJB9/XBP1 |
| BP | GO:0050878 | regulation of body fluid levels | 0.374048511 | 0.349846071 | PLAT/DGKI/XBP1/EMP2/MYO5B/TRAF3IP2/PDGFRA/GRHL1 |
| BP | GO:2000241 | regulation of reproductive process | 0.374048511 | 0.349846071 | NKX3-1/PLAT/AGO2/TACR3/RGN |
| BP | GO:2000736 | regulation of stem cell differentiation | 0.374048511 | 0.349846071 | STAT3/PDGFRA/NUDT21 |
| BP | GO:0071379 | cellular response to prostaglandin stimulus | 0.374048511 | 0.349846071 | GNG2/PRKAA2 |
| BP | GO:0045860 | positive regulation of protein kinase activity | 0.374048511 | 0.349846071 | FLT1/MAP3K4/EMP2/FCGR1A/IL23A/PRRC1/FGF13/FERMT2 |
| BP | GO:0033674 | positive regulation of kinase activity | 0.374048511 | 0.349846071 | FLT1/MAP3K4/EMP2/FCGR1A/IL23A/PRRC1/FGF13/FERMT2/PDGFRA |
| BP | GO:0043551 | regulation of phosphatidylinositol 3-kinase activity | 0.374048511 | 0.349846071 | SOCS3/FLT1/PDGFRA |
| BP | GO:0046394 | carboxylic acid biosynthetic process | 0.374048511 | 0.349846071 | PTGS2/XBP1/RGN/ELOVL7/PNPLA8/PRKAA2/CYP7B1 |
| BP | GO:0023019 | signal transduction involved in regulation of gene expression | 0.374048511 | 0.349846071 | TRAF3IP2/PDGFRA |
| BP | GO:0043373 | CD4-positive, alpha-beta T cell lineage commitment | 0.374048511 | 0.349846071 | IL23A/STAT3 |
| BP | GO:0016053 | organic acid biosynthetic process | 0.374048511 | 0.349846071 | PTGS2/XBP1/RGN/ELOVL7/PNPLA8/PRKAA2/CYP7B1 |
| BP | GO:0003341 | cilium movement | 0.374048511 | 0.349846071 | TTC29/TACR3/ADCY10/RGN/NME5 |
| BP | GO:0002244 | hematopoietic progenitor cell differentiation | 0.374048511 | 0.349846071 | FLT1/PDGFRA/PRRC2C/NUDT21 |
| BP | GO:1904892 | regulation of receptor signaling pathway via STAT | 0.374048511 | 0.349846071 | GADD45A/SOCS3/IFNA16/IL23A |
| BP | GO:0032613 | interleukin-10 production | 0.374048511 | 0.349846071 | IL23A/STAT3/TNFRSF21 |
| BP | GO:0032653 | regulation of interleukin-10 production | 0.374048511 | 0.349846071 | IL23A/STAT3/TNFRSF21 |
| BP | GO:0031668 | cellular response to extracellular stimulus | 0.374048511 | 0.349846071 | SH3GLB1/XBP1/ELAPOR1/CDKN2B/STK26/PRKAA2 |
| BP | GO:0006379 | mRNA cleavage | 0.374048511 | 0.349846071 | AGO2/NUDT21 |
| BP | GO:0032693 | negative regulation of interleukin-10 production | 0.374048511 | 0.349846071 | IL23A/TNFRSF21 |
| BP | GO:0046058 | cAMP metabolic process | 0.374048511 | 0.349846071 | PDE4B/ADCY10 |
| BP | GO:0061042 | vascular wound healing | 0.374048511 | 0.349846071 | XBP1/SMOC2 |
| BP | GO:0043410 | positive regulation of MAPK cascade | 0.374048511 | 0.349846071 | GADD45A/FLT1/MAP3K4/CCL20/FERMT2/MYDGF/SLAMF1/PDGFRA/GADD45B |
| BP | GO:0006109 | regulation of carbohydrate metabolic process | 0.374048511 | 0.349846071 | STAT3/RGN/EPM2AIP1/PPP1R3A/PRKAA2 |
| BP | GO:0048015 | phosphatidylinositol-mediated signaling | 0.374048511 | 0.349846071 | NKX3-1/FLT1/NEDD4/MYDGF/PDGFRA |
| BP | GO:0016239 | positive regulation of macroautophagy | 0.374048511 | 0.349846071 | SH3GLB1/ELAPOR1/PRKAA2 |
| BP | GO:2000242 | negative regulation of reproductive process | 0.374048511 | 0.349846071 | NKX3-1/PLAT/RGN |
| BP | GO:0046530 | photoreceptor cell differentiation | 0.374048511 | 0.349846071 | RPGRIP1/OLFM3/STAT3 |
| BP | GO:0002363 | alpha-beta T cell lineage commitment | 0.374048511 | 0.349846071 | IL23A/STAT3 |
| BP | GO:0033139 | regulation of peptidyl-serine phosphorylation of STAT protein | 0.374048511 | 0.349846071 | GADD45A/IFNA16 |
| BP | GO:0034695 | response to prostaglandin E | 0.374048511 | 0.349846071 | GNG2/PRKAA2 |
| BP | GO:0043369 | CD4-positive or CD8-positive, alpha-beta T cell lineage commitment | 0.374048511 | 0.349846071 | IL23A/STAT3 |
| BP | GO:0045723 | positive regulation of fatty acid biosynthetic process | 0.374048511 | 0.349846071 | PTGS2/RGN |
| BP | GO:0071636 | positive regulation of transforming growth factor beta production | 0.374048511 | 0.349846071 | PTGS2/SERPINB7 |
| BP | GO:0048017 | inositol lipid-mediated signaling | 0.375023399 | 0.35075788 | NKX3-1/FLT1/NEDD4/MYDGF/PDGFRA |
| BP | GO:0033692 | cellular polysaccharide biosynthetic process | 0.378313866 | 0.353835441 | HS2ST1/EPM2AIP1/PPP1R3A |
| BP | GO:0001783 | B cell apoptotic process | 0.379993711 | 0.355406593 | TRAF3IP2/TNFRSF21 |
| BP | GO:1902115 | regulation of organelle assembly | 0.379993711 | 0.355406593 | SH3GLB1/CSF2/ELAPOR1/KIF9/PRKAA2 |
| BP | GO:1902117 | positive regulation of organelle assembly | 0.379993711 | 0.355406593 | SH3GLB1/CSF2/ELAPOR1 |
| BP | GO:0060740 | prostate gland epithelium morphogenesis | 0.379993711 | 0.355406593 | NKX3-1/CYP7B1 |
| BP | GO:0072012 | glomerulus vasculature development | 0.379993711 | 0.355406593 | SERPINB7/PDGFRA |
| BP | GO:0071621 | granulocyte chemotaxis | 0.379993711 | 0.355406593 | PDE4B/CCL20/CXCL2/SLAMF1 |
| BP | GO:0001655 | urogenital system development | 0.379993711 | 0.355406593 | NKX3-1/SERPINB7/FBN1/RGN/TRAF3IP2/PDGFRA/CYP7B1 |
| BP | GO:0070192 | chromosome organization involved in meiotic cell cycle | 0.379993711 | 0.355406593 | HORMAD2/TEX15/MSH4 |
| BP | GO:0007568 | aging | 0.379993711 | 0.355406593 | PTGS2/TACR3/CDKN2A/CDKN2B/BCL2A1/STAT3/RGN |
| BP | GO:0032874 | positive regulation of stress-activated MAPK cascade | 0.379993711 | 0.355406593 | GADD45A/MAP3K4/SLAMF1/GADD45B |
| BP | GO:0042060 | wound healing | 0.379993711 | 0.355406593 | PLAT/KANK1/DGKI/XBP1/ARHGAP24/FERMT2/PDGFRA/SMOC2 |
| BP | GO:0034063 | stress granule assembly | 0.379993711 | 0.355406593 | PRRC2C/PRKAA2 |
| BP | GO:0060295 | regulation of cilium movement involved in cell motility | 0.379993711 | 0.355406593 | TACR3/RGN |
| BP | GO:1902019 | regulation of cilium-dependent cell motility | 0.379993711 | 0.355406593 | TACR3/RGN |
| BP | GO:0048608 | reproductive structure development | 0.379993711 | 0.355406593 | NKX3-1/PTGS2/SOCS3/CSF2/MAP3K4/PDGFRA/MSH4/CYP7B1 |
| BP | GO:0070304 | positive regulation of stress-activated protein kinase signaling cascade | 0.379993711 | 0.355406593 | GADD45A/MAP3K4/SLAMF1/GADD45B |
| BP | GO:0043550 | regulation of lipid kinase activity | 0.379993711 | 0.355406593 | SOCS3/FLT1/PDGFRA |
| BP | GO:0050795 | regulation of behavior | 0.379993711 | 0.355406593 | CSF2/TACR3/STAT3 |
| BP | GO:1903131 | mononuclear cell differentiation | 0.379993711 | 0.355406593 | DNAJB9/CSF2/XBP1/IFNA16/IL23A/STAT3/SLAMF1/TRAF3IP2 |
| BP | GO:0061458 | reproductive system development | 0.379993711 | 0.355406593 | NKX3-1/PTGS2/SOCS3/CSF2/MAP3K4/PDGFRA/MSH4/CYP7B1 |
| BP | GO:0000271 | polysaccharide biosynthetic process | 0.379993711 | 0.355406593 | HS2ST1/EPM2AIP1/PPP1R3A |
| BP | GO:0032729 | positive regulation of interferon-gamma production | 0.379993711 | 0.355406593 | PDE4B/IL23A/SLAMF1 |
| BP | GO:0002825 | regulation of T-helper 1 type immune response | 0.379993711 | 0.355406593 | IL23A/SLAMF1 |
| BP | GO:0042104 | positive regulation of activated T cell proliferation | 0.379993711 | 0.355406593 | IL23A/SLAMF1 |
| BP | GO:0042501 | serine phosphorylation of STAT protein | 0.379993711 | 0.355406593 | GADD45A/IFNA16 |
| BP | GO:0060512 | prostate gland morphogenesis | 0.379993711 | 0.355406593 | NKX3-1/CYP7B1 |
| BP | GO:0061437 | renal system vasculature development | 0.379993711 | 0.355406593 | SERPINB7/PDGFRA |
| BP | GO:0061440 | kidney vasculature development | 0.379993711 | 0.355406593 | SERPINB7/PDGFRA |
| BP | GO:0043467 | regulation of generation of precursor metabolites and energy | 0.379993711 | 0.355406593 | STAT3/EPM2AIP1/PPP1R3A/PRKAA2 |
| BP | GO:0042594 | response to starvation | 0.381090832 | 0.356432726 | SH3GLB1/XBP1/ELAPOR1/STK26/PRKAA2 |
| BP | GO:0035924 | cellular response to vascular endothelial growth factor stimulus | 0.382244011 | 0.357511289 | FLT1/PDGFRA/SMOC2 |
| BP | GO:0086003 | cardiac muscle cell contraction | 0.382244011 | 0.357511289 | PDE4B/GJC1/FGF13 |
| BP | GO:0032801 | receptor catabolic process | 0.389260712 | 0.364073982 | SH3GLB1/NEDD4 |
| BP | GO:0060259 | regulation of feeding behavior | 0.389260712 | 0.364073982 | TACR3/STAT3 |
| BP | GO:0032147 | activation of protein kinase activity | 0.391066895 | 0.365763298 | EMP2/IL23A/PRRC1/FGF13 |
| BP | GO:0045913 | positive regulation of carbohydrate metabolic process | 0.391066895 | 0.365763298 | RGN/EPM2AIP1/PRKAA2 |
| BP | GO:0016051 | carbohydrate biosynthetic process | 0.391066895 | 0.365763298 | HS2ST1/MGAT2/RGN/EPM2AIP1/PPP1R3A |
| BP | GO:0001773 | myeloid dendritic cell activation | 0.391066895 | 0.365763298 | CSF2/SLAMF1 |
| BP | GO:0002360 | T cell lineage commitment | 0.391066895 | 0.365763298 | IL23A/STAT3 |
| BP | GO:0005979 | regulation of glycogen biosynthetic process | 0.391066895 | 0.365763298 | EPM2AIP1/PPP1R3A |
| BP | GO:0010962 | regulation of glucan biosynthetic process | 0.391066895 | 0.365763298 | EPM2AIP1/PPP1R3A |
| BP | GO:0072539 | T-helper 17 cell differentiation | 0.391066895 | 0.365763298 | IL23A/STAT3 |
| BP | GO:0010631 | epithelial cell migration | 0.391066895 | 0.365763298 | GADD45A/PTGS2/KANK1/EMP2/EFNB2/MCC/SMOC2 |
| BP | GO:0043401 | steroid hormone mediated signaling pathway | 0.391066895 | 0.365763298 | NKX3-1/NEDD4/ESRRG/CYP7B1 |
| BP | GO:0090132 | epithelium migration | 0.391066895 | 0.365763298 | GADD45A/PTGS2/KANK1/EMP2/EFNB2/MCC/SMOC2 |
| BP | GO:0034694 | response to prostaglandin | 0.391066895 | 0.365763298 | GNG2/PRKAA2 |
| BP | GO:1900101 | regulation of endoplasmic reticulum unfolded protein response | 0.391066895 | 0.365763298 | DNAJB9/XBP1 |
| BP | GO:2000637 | positive regulation of gene silencing by miRNA | 0.391066895 | 0.365763298 | AGO2/STAT3 |
| BP | GO:0034637 | cellular carbohydrate biosynthetic process | 0.391066895 | 0.365763298 | HS2ST1/EPM2AIP1/PPP1R3A |
| BP | GO:0071222 | cellular response to lipopolysaccharide | 0.391066895 | 0.365763298 | CSF2/IL36G/PDE4B/XBP1/CXCL2 |
| BP | GO:0090130 | tissue migration | 0.391066895 | 0.365763298 | GADD45A/PTGS2/KANK1/EMP2/EFNB2/MCC/SMOC2 |
| BP | GO:0001516 | prostaglandin biosynthetic process | 0.391066895 | 0.365763298 | PTGS2/PNPLA8 |
| BP | GO:0002230 | positive regulation of defense response to virus by host | 0.391066895 | 0.365763298 | IL23A/TRAF3IP2 |
| BP | GO:0045987 | positive regulation of smooth muscle contraction | 0.391066895 | 0.365763298 | PTGS2/TACR3 |
| BP | GO:0046457 | prostanoid biosynthetic process | 0.391066895 | 0.365763298 | PTGS2/PNPLA8 |
| BP | GO:0060055 | angiogenesis involved in wound healing | 0.391066895 | 0.365763298 | XBP1/SMOC2 |
| BP | GO:0060148 | positive regulation of posttranscriptional gene silencing | 0.391066895 | 0.365763298 | AGO2/STAT3 |
| BP | GO:1900027 | regulation of ruffle assembly | 0.391066895 | 0.365763298 | KANK1/ARHGAP24 |
| BP | GO:0016241 | regulation of macroautophagy | 0.391066895 | 0.365763298 | SH3GLB1/ELAPOR1/NEDD4/PRKAA2 |
| BP | GO:0030183 | B cell differentiation | 0.391066895 | 0.365763298 | DNAJB9/XBP1/IFNA16/TRAF3IP2 |
| BP | GO:0032355 | response to estradiol | 0.391066895 | 0.365763298 | PTGS2/TACR3/STAT3/LCOR |
| BP | GO:0021766 | hippocampus development | 0.391066895 | 0.365763298 | FGF13/HSD3B1/NEUROD6 |
| BP | GO:0009150 | purine ribonucleotide metabolic process | 0.392720111 | 0.367309543 | ACOT12/PDE4B/ADCY10/STAT3/NME5/ELOVL7/PRKAA2 |
| BP | GO:0006469 | negative regulation of protein kinase activity | 0.392720111 | 0.367309543 | GADD45A/CDKN2A/CDKN2B/RGN/GADD45B |
| BP | GO:0097193 | intrinsic apoptotic signaling pathway | 0.392720111 | 0.367309543 | NKX3-1/PTGS2/XBP1/TP53BP2/BCL2A1/NME5 |
| BP | GO:0071260 | cellular response to mechanical stimulus | 0.393637291 | 0.368167378 | GADD45A/PTGS2/MTPN |
| BP | GO:0045736 | negative regulation of cyclin-dependent protein serine/threonine kinase activity | 0.394563338 | 0.369033506 | CDKN2A/CDKN2B |
| BP | GO:0001890 | placenta development | 0.394563338 | 0.369033506 | PTGS2/SOCS3/CSF2/MAP3K4 |
| BP | GO:0030098 | lymphocyte differentiation | 0.394563338 | 0.369033506 | DNAJB9/XBP1/IFNA16/IL23A/STAT3/SLAMF1/TRAF3IP2 |
| BP | GO:0001822 | kidney development | 0.394563338 | 0.369033506 | NKX3-1/SERPINB7/FBN1/RGN/TRAF3IP2/PDGFRA |
| BP | GO:0043552 | positive regulation of phosphatidylinositol 3-kinase activity | 0.394563338 | 0.369033506 | FLT1/PDGFRA |
| BP | GO:0120033 | negative regulation of plasma membrane bounded cell projection assembly | 0.394563338 | 0.369033506 | KANK1/ARHGAP24 |
| BP | GO:1904030 | negative regulation of cyclin-dependent protein kinase activity | 0.394563338 | 0.369033506 | CDKN2A/CDKN2B |
| BP | GO:0010675 | regulation of cellular carbohydrate metabolic process | 0.394563338 | 0.369033506 | STAT3/RGN/EPM2AIP1/PPP1R3A |
| BP | GO:0006112 | energy reserve metabolic process | 0.394563338 | 0.369033506 | GFPT2/EPM2AIP1/PPP1R3A |
| BP | GO:0006970 | response to osmotic stress | 0.394563338 | 0.369033506 | PTGS2/TACR3/NFAT5 |
| BP | GO:0097530 | granulocyte migration | 0.394563338 | 0.369033506 | PDE4B/CCL20/CXCL2/SLAMF1 |
| BP | GO:0001656 | metanephros development | 0.394563338 | 0.369033506 | NKX3-1/FBN1/PDGFRA |
| BP | GO:0042509 | regulation of tyrosine phosphorylation of STAT protein | 0.394563338 | 0.369033506 | SOCS3/CSF2/IL23A |
| BP | GO:0044319 | wound healing, spreading of cells | 0.394563338 | 0.369033506 | ARHGAP24/FERMT2 |
| BP | GO:0048536 | spleen development | 0.394563338 | 0.369033506 | CDKN2B/TRAF3IP2 |
| BP | GO:0086004 | regulation of cardiac muscle cell contraction | 0.394563338 | 0.369033506 | PDE4B/FGF13 |
| BP | GO:0090505 | epiboly involved in wound healing | 0.394563338 | 0.369033506 | ARHGAP24/FERMT2 |
| BP | GO:0098751 | bone cell development | 0.394563338 | 0.369033506 | SIGLEC15/FBN1 |
| BP | GO:0097529 | myeloid leukocyte migration | 0.394563338 | 0.369033506 | FLT1/PDE4B/CCL20/CXCL2/SLAMF1 |
| BP | GO:0045834 | positive regulation of lipid metabolic process | 0.394563338 | 0.369033506 | PTGS2/FLT1/RGN/PDGFRA |
| BP | GO:0010810 | regulation of cell-substrate adhesion | 0.394563338 | 0.369033506 | KANK1/EMP2/CDKN2A/ATXN3L/FERMT2 |
| BP | GO:0071219 | cellular response to molecule of bacterial origin | 0.394563338 | 0.369033506 | CSF2/IL36G/PDE4B/XBP1/CXCL2 |
| BP | GO:0001819 | positive regulation of cytokine production | 0.394563338 | 0.369033506 | PTGS2/CSF2/PDE4B/SERPINB7/XBP1/IL23A/STAT3/SLAMF1 |
| BP | GO:0048592 | eye morphogenesis | 0.394563338 | 0.369033506 | RPGRIP1/OLFM3/STAT3/FBN1 |
| BP | GO:0003351 | epithelial cilium movement involved in extracellular fluid movement | 0.394563338 | 0.369033506 | ADCY10/NME5 |
| BP | GO:0034405 | response to fluid shear stress | 0.394563338 | 0.369033506 | PTGS2/CSF2 |
| BP | GO:0035633 | maintenance of blood-brain barrier | 0.394563338 | 0.369033506 | PTGS2/LAMA2 |
| BP | GO:0042462 | eye photoreceptor cell development | 0.394563338 | 0.369033506 | RPGRIP1/OLFM3 |
| BP | GO:0046627 | negative regulation of insulin receptor signaling pathway | 0.394563338 | 0.369033506 | SOCS3/KANK1 |
| BP | GO:0090504 | epiboly | 0.394563338 | 0.369033506 | ARHGAP24/FERMT2 |
| BP | GO:0072001 | renal system development | 0.394563338 | 0.369033506 | NKX3-1/SERPINB7/FBN1/RGN/TRAF3IP2/PDGFRA |
| BP | GO:0009259 | ribonucleotide metabolic process | 0.394563338 | 0.369033506 | ACOT12/PDE4B/ADCY10/STAT3/NME5/ELOVL7/PRKAA2 |
| BP | GO:0042326 | negative regulation of phosphorylation | 0.394563338 | 0.369033506 | GADD45A/SOCS3/CDKN2A/CDKN2B/STAT3/RGN/GADD45B |
| BP | GO:0045786 | negative regulation of cell cycle | 0.394563338 | 0.369033506 | NKX3-1/PTGS2/TP53BP2/CDKN2A/CDKN2B/MAP3K20/NEK11 |
| BP | GO:0007260 | tyrosine phosphorylation of STAT protein | 0.397972854 | 0.372222413 | SOCS3/CSF2/IL23A |
| BP | GO:0003352 | regulation of cilium movement | 0.397972854 | 0.372222413 | TACR3/RGN |
| BP | GO:0070873 | regulation of glycogen metabolic process | 0.397972854 | 0.372222413 | EPM2AIP1/PPP1R3A |
| BP | GO:0097009 | energy homeostasis | 0.397972854 | 0.372222413 | STAT3/PRKAA2 |
| BP | GO:0001667 | ameboidal-type cell migration | 0.397972854 | 0.372222413 | GADD45A/PTGS2/AGO2/KANK1/EMP2/EFNB2/MCC/SMOC2 |
| BP | GO:0046330 | positive regulation of JNK cascade | 0.397972854 | 0.372222413 | GADD45A/SLAMF1/GADD45B |
| BP | GO:1900407 | regulation of cellular response to oxidative stress | 0.397972854 | 0.372222413 | STK26/NME5/PNPLA8 |
| BP | GO:0006631 | fatty acid metabolic process | 0.400994141 | 0.375048211 | ACOT12/PTGS2/XBP1/RGN/ELOVL7/PNPLA8/PRKAA2 |
| BP | GO:0045444 | fat cell differentiation | 0.400994141 | 0.375048211 | PTGS2/XBP1/PLAC8/FERMT2/PDGFRA |
| BP | GO:0032885 | regulation of polysaccharide biosynthetic process | 0.400994141 | 0.375048211 | EPM2AIP1/PPP1R3A |
| BP | GO:0040036 | regulation of fibroblast growth factor receptor signaling pathway | 0.400994141 | 0.375048211 | OTX2/SMOC2 |
| BP | GO:0043368 | positive T cell selection | 0.400994141 | 0.375048211 | IL23A/STAT3 |
| BP | GO:0090218 | positive regulation of lipid kinase activity | 0.400994141 | 0.375048211 | FLT1/PDGFRA |
| BP | GO:1900077 | negative regulation of cellular response to insulin stimulus | 0.400994141 | 0.375048211 | SOCS3/KANK1 |
| BP | GO:0006163 | purine nucleotide metabolic process | 0.411182231 | 0.384577091 | ACOT12/PDE4B/ADCY10/STAT3/NME5/ELOVL7/PRKAA2 |
| BP | GO:0019693 | ribose phosphate metabolic process | 0.411182231 | 0.384577091 | ACOT12/PDE4B/ADCY10/STAT3/NME5/ELOVL7/PRKAA2 |
| BP | GO:0006858 | extracellular transport | 0.411182231 | 0.384577091 | ADCY10/NME5 |
| BP | GO:1903580 | positive regulation of ATP metabolic process | 0.411182231 | 0.384577091 | STAT3/PRKAA2 |
| BP | GO:2000279 | negative regulation of DNA biosynthetic process | 0.411182231 | 0.384577091 | RGN/KCNK2 |
| BP | GO:0032970 | regulation of actin filament-based process | 0.411632846 | 0.384998549 | SIGLEC15/KANK1/MTPN/PDE4B/FGF13/FERMT2/PDGFRA |
| BP | GO:0045132 | meiotic chromosome segregation | 0.412952992 | 0.386233276 | HORMAD2/TEX15/MSH4 |
| BP | GO:0000079 | regulation of cyclin-dependent protein serine/threonine kinase activity | 0.412952992 | 0.386233276 | GADD45A/CDKN2A/CDKN2B |
| BP | GO:0030316 | osteoclast differentiation | 0.412952992 | 0.386233276 | SIGLEC15/IL23A/FBN1 |
| BP | GO:0016572 | histone phosphorylation | 0.412952992 | 0.386233276 | PRKAA2/NEK11 |
| BP | GO:0045923 | positive regulation of fatty acid metabolic process | 0.412952992 | 0.386233276 | PTGS2/RGN |
| BP | GO:1903115 | regulation of actin filament-based movement | 0.412952992 | 0.386233276 | PDE4B/FGF13 |
| BP | GO:2000785 | regulation of autophagosome assembly | 0.412952992 | 0.386233276 | SH3GLB1/ELAPOR1 |
| BP | GO:0033673 | negative regulation of kinase activity | 0.412952992 | 0.386233276 | GADD45A/CDKN2A/CDKN2B/RGN/GADD45B |
| BP | GO:0009187 | cyclic nucleotide metabolic process | 0.412952992 | 0.386233276 | PDE4B/ADCY10 |
| BP | GO:0010863 | positive regulation of phospholipase C activity | 0.412952992 | 0.386233276 | FLT1/PDGFRA |
| BP | GO:0051403 | stress-activated MAPK cascade | 0.412952992 | 0.386233276 | GADD45A/MAP3K4/MAP3K20/SLAMF1/GADD45B |
| BP | GO:0044264 | cellular polysaccharide metabolic process | 0.412952992 | 0.386233276 | HS2ST1/EPM2AIP1/PPP1R3A |
| BP | GO:2001242 | regulation of intrinsic apoptotic signaling pathway | 0.412952992 | 0.386233276 | NKX3-1/PTGS2/XBP1/NME5 |
| BP | GO:0010907 | positive regulation of glucose metabolic process | 0.412952992 | 0.386233276 | RGN/EPM2AIP1 |
| BP | GO:0032733 | positive regulation of interleukin-10 production | 0.412952992 | 0.386233276 | IL23A/STAT3 |
| BP | GO:0050691 | regulation of defense response to virus by host | 0.412952992 | 0.386233276 | IL23A/TRAF3IP2 |
| BP | GO:0071634 | regulation of transforming growth factor beta production | 0.412952992 | 0.386233276 | PTGS2/SERPINB7 |
| BP | GO:1902882 | regulation of response to oxidative stress | 0.412952992 | 0.386233276 | STK26/NME5/PNPLA8 |
| BP | GO:1904029 | regulation of cyclin-dependent protein kinase activity | 0.412952992 | 0.386233276 | GADD45A/CDKN2A/CDKN2B |
| BP | GO:2001243 | negative regulation of intrinsic apoptotic signaling pathway | 0.412952992 | 0.386233276 | PTGS2/XBP1/NME5 |
| BP | GO:0007254 | JNK cascade | 0.412952992 | 0.386233276 | GADD45A/MAP3K20/SLAMF1/GADD45B |
| BP | GO:0055088 | lipid homeostasis | 0.412952992 | 0.386233276 | XBP1/PNPLA8/PRKAA2/CYP7B1 |
| BP | GO:0006165 | nucleoside diphosphate phosphorylation | 0.412952992 | 0.386233276 | STAT3/NME5/PRKAA2 |
| BP | GO:0006040 | amino sugar metabolic process | 0.412952992 | 0.386233276 | GFPT2/CHI3L2 |
| BP | GO:0055090 | acylglycerol homeostasis | 0.412952992 | 0.386233276 | XBP1/PNPLA8 |
| BP | GO:0070328 | triglyceride homeostasis | 0.412952992 | 0.386233276 | XBP1/PNPLA8 |
| BP | GO:1900274 | regulation of phospholipase C activity | 0.412952992 | 0.386233276 | FLT1/PDGFRA |
| BP | GO:0043434 | response to peptide hormone | 0.412952992 | 0.386233276 | PTGS2/SOCS3/KANK1/XBP1/STAT3/FBN1/EPM2AIP1 |
| BP | GO:0006937 | regulation of muscle contraction | 0.412952992 | 0.386233276 | PTGS2/PDE4B/TACR3/FGF13 |
| BP | GO:0009152 | purine ribonucleotide biosynthetic process | 0.412952992 | 0.386233276 | ADCY10/STAT3/NME5/ELOVL7 |
| BP | GO:0021543 | pallium development | 0.412952992 | 0.386233276 | FGF13/BCL2A1/HSD3B1/NEUROD6 |
| BP | GO:0071216 | cellular response to biotic stimulus | 0.412952992 | 0.386233276 | CSF2/IL36G/PDE4B/XBP1/CXCL2 |
| BP | GO:0031098 | stress-activated protein kinase signaling cascade | 0.412952992 | 0.386233276 | GADD45A/MAP3K4/MAP3K20/SLAMF1/GADD45B |
| BP | GO:0072521 | purine-containing compound metabolic process | 0.412952992 | 0.386233276 | ACOT12/PDE4B/ADCY10/STAT3/NME5/ELOVL7/PRKAA2 |
| BP | GO:0042088 | T-helper 1 type immune response | 0.412952992 | 0.386233276 | IL23A/SLAMF1 |
| BP | GO:0045981 | positive regulation of nucleotide metabolic process | 0.412952992 | 0.386233276 | STAT3/PRKAA2 |
| BP | GO:0071604 | transforming growth factor beta production | 0.412952992 | 0.386233276 | PTGS2/SERPINB7 |
| BP | GO:1900544 | positive regulation of purine nucleotide metabolic process | 0.412952992 | 0.386233276 | STAT3/PRKAA2 |
| BP | GO:0006476 | protein deacetylation | 0.412952992 | 0.386233276 | SAP30BP/ATXN3L/PRKAA2 |
| BP | GO:0006576 | cellular biogenic amine metabolic process | 0.412952992 | 0.386233276 | MTPN/TACR3/OAZ3 |
| BP | GO:0046939 | nucleotide phosphorylation | 0.412952992 | 0.386233276 | STAT3/NME5/PRKAA2 |
| BP | GO:0045926 | negative regulation of growth | 0.412952992 | 0.386233276 | CDKN2A/PLAC8/FGF13/KCNK2/CGRRF1 |
| BP | GO:0005978 | glycogen biosynthetic process | 0.412952992 | 0.386233276 | EPM2AIP1/PPP1R3A |
| BP | GO:0009250 | glucan biosynthetic process | 0.412952992 | 0.386233276 | EPM2AIP1/PPP1R3A |
| BP | GO:0032881 | regulation of polysaccharide metabolic process | 0.412952992 | 0.386233276 | EPM2AIP1/PPP1R3A |
| BP | GO:0046006 | regulation of activated T cell proliferation | 0.412952992 | 0.386233276 | IL23A/SLAMF1 |
| BP | GO:0097178 | ruffle assembly | 0.412952992 | 0.386233276 | KANK1/ARHGAP24 |
| BP | GO:1903573 | negative regulation of response to endoplasmic reticulum stress | 0.412952992 | 0.386233276 | DNAJB9/XBP1 |
| BP | GO:0062012 | regulation of small molecule metabolic process | 0.412952992 | 0.386233276 | PTGS2/STAT3/RGN/EPM2AIP1/PPP1R3A/PRKAA2 |
| BP | GO:0030593 | neutrophil chemotaxis | 0.412952992 | 0.386233276 | PDE4B/CCL20/CXCL2 |
| BP | GO:0001659 | temperature homeostasis | 0.412952992 | 0.386233276 | PTGS2/PLAC8/ESRRG/STAT3 |
| BP | GO:0090257 | regulation of muscle system process | 0.412952992 | 0.386233276 | PTGS2/MTPN/PDE4B/TACR3/FGF13 |
| BP | GO:0034612 | response to tumor necrosis factor | 0.412952992 | 0.386233276 | NKX3-1/PTGS2/CCL20/TRAF3IP2/TNFRSF21 |
| BP | GO:0062197 | cellular response to chemical stress | 0.412952992 | 0.386233276 | PTGS2/STK26/NME5/PDGFRA/PNPLA8/PRKAA2 |
| BP | GO:0006110 | regulation of glycolytic process | 0.412952992 | 0.386233276 | STAT3/PRKAA2 |
| BP | GO:0030850 | prostate gland development | 0.412952992 | 0.386233276 | NKX3-1/CYP7B1 |
| BP | GO:0031670 | cellular response to nutrient | 0.412952992 | 0.386233276 | XBP1/CDKN2B |
| BP | GO:0044088 | regulation of vacuole organization | 0.412952992 | 0.386233276 | SH3GLB1/ELAPOR1 |
| BP | GO:0045933 | positive regulation of muscle contraction | 0.412952992 | 0.386233276 | PTGS2/TACR3 |
| BP | GO:0043534 | blood vessel endothelial cell migration | 0.412952992 | 0.386233276 | GADD45A/PTGS2/EMP2/EFNB2 |
| BP | GO:0043547 | positive regulation of GTPase activity | 0.412952992 | 0.386233276 | CCL20/ARHGAP24/RGN/FERMT2/RALGAPA1 |
| BP | GO:0048545 | response to steroid hormone | 0.412952992 | 0.386233276 | NKX3-1/PTGS2/NEDD4/ESRRG/HSD3B1/CYP7B1 |
| BP | GO:0090596 | sensory organ morphogenesis | 0.412952992 | 0.386233276 | RPGRIP1/CTHRC1/OLFM3/STAT3/FBN1 |
| BP | GO:1903522 | regulation of blood circulation | 0.412952992 | 0.386233276 | PTGS2/PDE4B/TACR3/GJC1/FGF13 |
| BP | GO:0071887 | leukocyte apoptotic process | 0.412952992 | 0.386233276 | CDKN2A/TRAF3IP2/TNFRSF21 |
| CC | GO:0045177 | apical part of cell | 0.726994642 | 0.726994642 | PLAT/EMP2/ADCY10/KCNK2/STK26/MYO5B/ADGRG2/SLC14A2 |
| CC | GO:0036464 | cytoplasmic ribonucleoprotein granule | 0.726994642 | 0.726994642 | AGO2/NYNRIN/MCC/PRRC2C/PRKAA2 |
| CC | GO:0005604 | basement membrane | 0.726994642 | 0.726994642 | FBN1/LAMA2/SMOC2 |
| CC | GO:0000795 | synaptonemal complex | 0.726994642 | 0.726994642 | HORMAD2/MSH4 |
| CC | GO:0099086 | synaptonemal structure | 0.726994642 | 0.726994642 | HORMAD2/MSH4 |
| CC | GO:0031234 | extrinsic component of cytoplasmic side of plasma membrane | 0.726994642 | 0.726994642 | FERMT2/KCNAB1/GNG2 |
| MF | GO:0005125 | cytokine activity | 0.257904338 | 0.253717991 | CSF2/IL36G/IFNA16/CCL20/IL23A/CXCL2/NDP/GDF11 |
| MF | GO:0005126 | cytokine receptor binding | 0.319165605 | 0.313984855 | CSF2/IL36G/IFNA16/CCL20/IL23A/CXCL2/STAT3/FERMT2 |
| MF | GO:0004861 | cyclin-dependent protein serine/threonine kinase inhibitor activity | 0.372934895 | 0.366881353 | CDKN2A/CDKN2B |
| MF | GO:0051219 | phosphoprotein binding | 0.372934895 | 0.366881353 | SOCS3/PLAT/NEDD4/LRP11 |
| MF | GO:0048018 | receptor ligand activity | 0.372934895 | 0.366881353 | CSF2/IL36G/IFNA16/CCL20/IL23A/CXCL2/FGF13/FBN1/NDP/GDF11 |
| MF | GO:0030546 | signaling receptor activator activity | 0.372934895 | 0.366881353 | CSF2/IL36G/IFNA16/CCL20/IL23A/CXCL2/FGF13/FBN1/NDP/GDF11 |
| MF | GO:0004879 | nuclear receptor activity | 0.372934895 | 0.366881353 | NKX3-1/ESRRG/STAT3 |
| MF | GO:0098531 | ligand-activated transcription factor activity | 0.372934895 | 0.366881353 | NKX3-1/ESRRG/STAT3 |
| MF | GO:0004860 | protein kinase inhibitor activity | 0.480953476 | 0.473146558 | SOCS3/CDKN2A/CDKN2B |
| MF | GO:0004709 | MAP kinase kinase kinase activity | 0.480953476 | 0.473146558 | MAP3K4/MAP3K20 |
| MF | GO:0042379 | chemokine receptor binding | 0.480953476 | 0.473146558 | CCL20/CXCL2/STAT3 |
| MF | GO:0019210 | kinase inhibitor activity | 0.480953476 | 0.473146558 | SOCS3/CDKN2A/CDKN2B |
| MF | GO:0051059 | NF-kappaB binding | 0.480953476 | 0.473146558 | TP53BP2/CDKN2A |
| MF | GO:0016922 | nuclear receptor binding | 0.480953476 | 0.473146558 | NKX3-1/MED14/STAT3/LCOR |
| MF | GO:0030291 | protein serine/threonine kinase inhibitor activity | 0.480953476 | 0.473146558 | CDKN2A/CDKN2B |
| MF | GO:0052689 | carboxylic ester hydrolase activity | 0.480953476 | 0.473146558 | ACOT12/RGN/AADACL2/PNPLA8 |
| MF | GO:0001664 | G protein-coupled receptor binding | 0.480953476 | 0.473146558 | CCL20/CTHRC1/NEDD4/CXCL2/STAT3/NDP |
| MF | GO:0005109 | frizzled binding | 0.480953476 | 0.473146558 | CTHRC1/NDP |
| MF | GO:0061629 | RNA polymerase II-specific DNA-binding transcription factor binding | 0.480953476 | 0.473146558 | NKX3-1/TP53BP2/CDKN2A/MED14/STAT3/LCOR |
| MF | GO:0017080 | sodium channel regulator activity | 0.480953476 | 0.473146558 | NEDD4/FGF13 |
| MF | GO:0030331 | estrogen receptor binding | 0.480953476 | 0.473146558 | NKX3-1/LCOR |
| KEGG | hsa05222 | Description | 0.04665687 |  | GADD45A/PTGS2/CDKN2B/LAMA2/GADD45B |
| KEGG | hsa05323 | Small cell lung cancer | 0.04665687 |  | CSF2/FLT1/CCL20/IL23A/CXCL2 |
| KEGG | hsa04657 | Rheumatoid arthritis | 0.04665687 |  | PTGS2/CSF2/CCL20/CXCL2/TRAF3IP2 |
| KEGG | hsa05202 | IL-17 signaling pathway | 0.04665687 |  | GADD45A/CSF2/PLAT/FLT1/FCGR1A/BCL2A1/GADD45B |
| KEGG | hsa04064 | Transcriptional misregulation in cancer | 0.04665687 |  | GADD45A/PTGS2/CXCL2/BCL2A1/GADD45B |
| KEGG | hsa04931 | NF-kappa B signaling pathway | 0.04665687 |  | GFPT2/SOCS3/STAT3/PPP1R3A/PRKAA2 |
| KEGG | hsa04630 | Insulin resistance | 0.04665687 |  | SOCS3/CSF2/IFNA16/IL23A/STAT3/PDGFRA |
| KEGG | hsa05221 | JAK-STAT signaling pathway | 0.04665687 |  | CSF2/FCGR1A/BCL2A1/STAT3 |
| KEGG | hsa04668 | Acute myeloid leukemia | 0.04665687 |  | PTGS2/SOCS3/CSF2/CCL20/CXCL2 |
| KEGG | hsa05218 | TNF signaling pathway | 0.04665687 |  | GADD45A/CDKN2A/PDGFRA/GADD45B |
| KEGG | hsa05223 | Melanoma | 0.04665687 |  | GADD45A/CDKN2A/STAT3/GADD45B |
| KEGG | hsa04060 | Non-small cell lung cancer | 0.04665687 |  | CSF2/IL36G/IFNA16/CCL20/IL23A/CXCL2/TNFRSF21/GDF11 |
| KEGG | hsa05214 | Cytokine-cytokine receptor interaction | 0.04665687 |  | GADD45A/CDKN2A/PDGFRA/GADD45B |
| KEGG | hsa05212 | Glioma | 0.04665687 |  | GADD45A/CDKN2A/STAT3/GADD45B |
| KEGG | hsa04068 | Pancreatic cancer | 0.055949148 |  | GADD45A/CDKN2B/STAT3/GADD45B/PRKAA2 |
| KEGG | hsa05167 | FoxO signaling pathway | 0.06212892 |  | PTGS2/CSF2/IFNA16/CXCL2/STAT3/GNG2 |
| KEGG | hsa04218 | Kaposi sarcoma-associated herpesvirus infection | 0.101841467 |  | GADD45A/CDKN2A/CDKN2B/TRAF3IP2/GADD45B |
| KEGG | hsa05163 | Cellular senescence | 0.110840681 |  | PTGS2/IFNA16/CDKN2A/STAT3/PDGFRA/GNG2 |
| KEGG | hsa00140 | Human cytomegalovirus infection | 0.132718029 |  | UGT2B15/HSD3B1/CYP7B1 |
| KEGG | hsa04920 | Steroid hormone biosynthesis | 0.174996871 |  | SOCS3/STAT3/PRKAA2 |
| KEGG | hsa04110 | Adipocytokine signaling pathway | 0.177264074 |  | GADD45A/CDKN2A/CDKN2B/GADD45B |
| KEGG | hsa04115 | Cell cycle | 0.18440799 |  | GADD45A/CDKN2A/GADD45B |
| KEGG | hsa05220 | p53 signaling pathway | 0.188300977 |  | GADD45A/CDKN2A/GADD45B |
| KEGG | hsa00053 | Chronic myeloid leukemia | 0.188300977 |  | RGN/UGT2B15 |
| KEGG | hsa05169 | Ascorbate and aldarate metabolism | 0.190327881 |  | GADD45A/IFNA16/NEDD4/STAT3/GADD45B |
| KEGG | hsa04371 | Epstein-Barr virus infection | 0.196429715 |  | PLAT/ADCY10/GNG2/PRKAA2 |
| KEGG | hsa05417 | Apelin signaling pathway | 0.222168474 |  | XBP1/IFNA16/CXCL2/POU2F3/STAT3 |
| KEGG | hsa04010 | Lipid and atherosclerosis | 0.229518443 |  | GADD45A/FLT1/MAP3K4/MAP3K20/PDGFRA/GADD45B |
| KEGG | hsa05216 | MAPK signaling pathway | 0.229758552 |  | GADD45A/GADD45B |
| KEGG | hsa04934 | Thyroid cancer | 0.239395967 |  | CDKN2A/CDKN2B/KCNK2/HSD3B1 |
| KEGG | hsa05215 | Cushing syndrome | 0.269145482 |  | NKX3-1/PLAT/PDGFRA |
