# Supplementary material for: Gastric Cancer Subtypes in Tumour and Nontumour Tissues by Immunologic and Hallmark Gene Sets
Source: J Oncol. 2022 Aug 27;2022:7887711. doi: 10.1155/2022/7887711 (PMC9440817; doi:10.1155/2022/7887711)
Supplement: Supplementary Materials — Supplementary Table S1. The association between clinical features and subtypes. Supplementary Table S2. Detailed information on GO and KEGG enrichment analyses of N gene sets. Supplementary Table S3. Detailed information on GO and KEGG enrichment analyses of T gene sets. Figure S1. The protein-protein interaction network for N gene sets. Figure S2. The protein-protein interaction network for T gene sets. [file 7887711.f1.zip › TableS3.docx]

**Supplementary TABLE S3 |** Detailed information on GO and KEGG enrichment analysis of T gene sets.

| ONTOLOGY | ID | Description | p.adjust | qvalue | geneID |
| --- | --- | --- | --- | --- | --- |
| BP | GO:0042060 | wound healing | 3.74E-07 | 2.10E-07 | POSTN/VEGFA/PF4/THBD/COL3A1/PDGFA/PTK2/VAV2/VTN/TIMP1/APOH |
| BP | GO:0007160 | cell-matrix adhesion | 8.81E-06 | 4.95E-06 | POSTN/VEGFA/ITGAV/JAG1/COL3A1/NRP1/PTK2/VTN |
| BP | GO:0048010 | vascular endothelial growth factor receptor signaling pathway | 4.70E-05 | 2.64E-05 | VEGFA/NRP1/PTK2/VAV2/VTN |
| BP | GO:0007596 | blood coagulation | 4.80E-05 | 2.69E-05 | PF4/THBD/COL3A1/PDGFA/VAV2/VTN/APOH |
| BP | GO:0007599 | hemostasis | 4.80E-05 | 2.69E-05 | PF4/THBD/COL3A1/PDGFA/VAV2/VTN/APOH |
| BP | GO:0050817 | coagulation | 4.80E-05 | 2.69E-05 | PF4/THBD/COL3A1/PDGFA/VAV2/VTN/APOH |
| BP | GO:0031589 | cell-substrate adhesion | 7.66E-05 | 4.30E-05 | POSTN/VEGFA/ITGAV/JAG1/COL3A1/NRP1/PTK2/VTN |
| BP | GO:0050878 | regulation of body fluid levels | 9.30E-05 | 5.22E-05 | VEGFA/PF4/THBD/COL3A1/PDGFA/VAV2/VTN/APOH |
| BP | GO:1903034 | regulation of response to wounding | 0.000122968 | 6.90E-05 | THBD/SPP1/PDGFA/PTK2/VTN/APOH |
| BP | GO:0043542 | endothelial cell migration | 0.000133778 | 7.51E-05 | FSTL1/STC1/VEGFA/FGFR1/NRP1/PTK2/APOH |
| BP | GO:1903035 | negative regulation of response to wounding | 0.000133778 | 7.51E-05 | THBD/SPP1/PDGFA/VTN/APOH |
| BP | GO:0032103 | positive regulation of response to external stimulus | 0.000152121 | 8.54E-05 | LPL/VEGFA/THBD/FGFR1/APP/NRP1/PTK2/APOH |
| BP | GO:0007229 | integrin-mediated signaling pathway | 0.000215327 | 0.000120905 | ITGAV/COL3A1/NRP1/PTK2/TIMP1 |
| BP | GO:0030195 | negative regulation of blood coagulation | 0.000252359 | 0.000141698 | THBD/PDGFA/VTN/APOH |
| BP | GO:1900047 | negative regulation of hemostasis | 0.000252447 | 0.000141748 | THBD/PDGFA/VTN/APOH |
| BP | GO:0001667 | ameboidal-type cell migration | 0.000252447 | 0.000141748 | FSTL1/STC1/VEGFA/FGFR1/NRP1/PTK2/TIMP1/APOH |
| BP | GO:0045446 | endothelial cell differentiation | 0.00025559 | 0.000143513 | FSTL1/STC1/VEGFA/JAG1/NRP1 |
| BP | GO:0007566 | embryo implantation | 0.00025559 | 0.000143513 | STC1/VEGFA/SPP1/TIMP1 |
| BP | GO:0050819 | negative regulation of coagulation | 0.00025559 | 0.000143513 | THBD/PDGFA/VTN/APOH |
| BP | GO:0030168 | platelet activation | 0.000278252 | 0.000156237 | PF4/THBD/COL3A1/PDGFA/VAV2 |
| BP | GO:0001952 | regulation of cell-matrix adhesion | 0.000299445 | 0.000168137 | POSTN/VEGFA/JAG1/NRP1/PTK2 |
| BP | GO:0010631 | epithelial cell migration | 0.000299445 | 0.000168137 | FSTL1/STC1/VEGFA/FGFR1/NRP1/PTK2/APOH |
| BP | GO:0090132 | epithelium migration | 0.000299445 | 0.000168137 | FSTL1/STC1/VEGFA/FGFR1/NRP1/PTK2/APOH |
| BP | GO:0010594 | regulation of endothelial cell migration | 0.000299445 | 0.000168137 | STC1/VEGFA/FGFR1/NRP1/PTK2/APOH |
| BP | GO:0090303 | positive regulation of wound healing | 0.000299445 | 0.000168137 | THBD/PTK2/VTN/APOH |
| BP | GO:0090130 | tissue migration | 0.000307101 | 0.000172435 | FSTL1/STC1/VEGFA/FGFR1/NRP1/PTK2/APOH |
| BP | GO:0061041 | regulation of wound healing | 0.000313667 | 0.000176122 | THBD/PDGFA/PTK2/VTN/APOH |
| BP | GO:0003158 | endothelium development | 0.000325186 | 0.00018259 | FSTL1/STC1/VEGFA/JAG1/NRP1 |
| BP | GO:0018108 | peptidyl-tyrosine phosphorylation | 0.000328666 | 0.000184544 | VEGFA/FGFR1/APP/NRP1/PDGFA/PTK2/VTN |
| BP | GO:0018212 | peptidyl-tyrosine modification | 0.000334708 | 0.000187937 | VEGFA/FGFR1/APP/NRP1/PDGFA/PTK2/VTN |
| BP | GO:0030193 | regulation of blood coagulation | 0.000378768 | 0.000212676 | THBD/PDGFA/VTN/APOH |
| BP | GO:1900046 | regulation of hemostasis | 0.000413486 | 0.00023217 | THBD/PDGFA/VTN/APOH |
| BP | GO:0050818 | regulation of coagulation | 0.000476419 | 0.000267507 | THBD/PDGFA/VTN/APOH |
| BP | GO:1903036 | positive regulation of response to wounding | 0.000488938 | 0.000274536 | THBD/PTK2/VTN/APOH |
| BP | GO:0032102 | negative regulation of response to external stimulus | 0.000569476 | 0.000319758 | THBD/SPP1/NRP1/PDGFA/PGLYRP1/VTN/APOH |
| BP | GO:0061045 | negative regulation of wound healing | 0.000635023 | 0.000356562 | THBD/PDGFA/VTN/APOH |
| BP | GO:0010632 | regulation of epithelial cell migration | 0.000748012 | 0.000420005 | STC1/VEGFA/FGFR1/NRP1/PTK2/APOH |
| BP | GO:0014910 | regulation of smooth muscle cell migration | 0.000994101 | 0.000558182 | POSTN/NRP1/PDGFA/VTN |
| BP | GO:0060326 | cell chemotaxis | 0.000994101 | 0.000558182 | VEGFA/PF4/FGFR1/NRP1/PTK2/CXCL6 |
| BP | GO:0010743 | regulation of macrophage derived foam cell differentiation | 0.001171228 | 0.000657638 | LPL/PF4/ITGAV |
| BP | GO:0035767 | endothelial cell chemotaxis | 0.001171228 | 0.000657638 | VEGFA/FGFR1/NRP1 |
| BP | GO:0007565 | female pregnancy | 0.001179044 | 0.000662027 | STC1/VEGFA/THBD/SPP1/TIMP1 |
| BP | GO:0014909 | smooth muscle cell migration | 0.001258282 | 0.000706519 | POSTN/NRP1/PDGFA/VTN |
| BP | GO:0032642 | regulation of chemokine production | 0.001280261 | 0.00071886 | POSTN/LPL/APP/CXCL6 |
| BP | GO:0032602 | chemokine production | 0.001302737 | 0.00073148 | POSTN/LPL/APP/CXCL6 |
| BP | GO:0010742 | macrophage derived foam cell differentiation | 0.001717452 | 0.00096434 | LPL/PF4/ITGAV |
| BP | GO:0090077 | foam cell differentiation | 0.001717452 | 0.00096434 | LPL/PF4/ITGAV |
| BP | GO:0034446 | substrate adhesion-dependent cell spreading | 0.001717452 | 0.00096434 | POSTN/ITGAV/NRP1/PTK2 |
| BP | GO:0014812 | muscle cell migration | 0.001771303 | 0.000994577 | POSTN/NRP1/PDGFA/VTN |
| BP | GO:0048259 | regulation of receptor-mediated endocytosis | 0.001771303 | 0.000994577 | LRPAP1/VEGFA/ITGAV/VTN |
| BP | GO:0044706 | multi-multicellular organism process | 0.001811878 | 0.00101736 | STC1/VEGFA/THBD/SPP1/TIMP1 |
| BP | GO:0010810 | regulation of cell-substrate adhesion | 0.001815657 | 0.001019482 | POSTN/VEGFA/JAG1/NRP1/PTK2 |
| BP | GO:0048588 | developmental cell growth | 0.002291198 | 0.001286495 | POSTN/VEGFA/APP/SPP1/NRP1 |
| BP | GO:0060560 | developmental growth involved in morphogenesis | 0.002291198 | 0.001286495 | POSTN/VEGFA/APP/SPP1/NRP1 |
| BP | GO:0048762 | mesenchymal cell differentiation | 0.002341709 | 0.001314857 | FGFR1/JAG1/NRP1/S100A4/MSX1 |
| BP | GO:0035987 | endodermal cell differentiation | 0.002411464 | 0.001354024 | COL5A2/ITGAV/VTN |
| BP | GO:0040013 | negative regulation of locomotion | 0.002473309 | 0.00138875 | STC1/JAG1/COL3A1/NRP1/TIMP1/APOH |
| BP | GO:0014911 | positive regulation of smooth muscle cell migration | 0.002653557 | 0.001489958 | POSTN/NRP1/VTN |
| BP | GO:0003007 | heart morphogenesis | 0.002654071 | 0.001490247 | VEGFA/JAG1/NRP1/PTK2/MSX1 |
| BP | GO:0008038 | neuron recognition | 0.002826181 | 0.001586885 | TNFRSF21/APP/NRP1 |
| BP | GO:0030225 | macrophage differentiation | 0.002826181 | 0.001586885 | VEGFA/PF4/APP |
| BP | GO:0010951 | negative regulation of endopeptidase activity | 0.002826181 | 0.001586885 | VEGFA/SERPINA5/APP/VTN/TIMP1 |
| BP | GO:0001503 | ossification | 0.002826181 | 0.001586885 | VCAN/STC1/COL5A2/JAG1/SPP1/PTK2 |
| BP | GO:0010466 | negative regulation of peptidase activity | 0.003287758 | 0.001846058 | VEGFA/SERPINA5/APP/VTN/TIMP1 |
| BP | GO:0050730 | regulation of peptidyl-tyrosine phosphorylation | 0.00335433 | 0.001883438 | VEGFA/APP/NRP1/PDGFA/VTN |
| BP | GO:0050921 | positive regulation of chemotaxis | 0.003481555 | 0.001954874 | VEGFA/FGFR1/NRP1/PTK2 |
| BP | GO:0001706 | endoderm formation | 0.003481555 | 0.001954874 | COL5A2/ITGAV/VTN |
| BP | GO:0051918 | negative regulation of fibrinolysis | 0.003630292 | 0.002038389 | THBD/APOH |
| BP | GO:0048008 | platelet-derived growth factor receptor signaling pathway | 0.003768114 | 0.002115776 | VEGFA/NRP1/PDGFA |
| BP | GO:1900024 | regulation of substrate adhesion-dependent cell spreading | 0.003915463 | 0.002198511 | POSTN/NRP1/PTK2 |
| BP | GO:0010876 | lipid localization | 0.004025007 | 0.00226002 | LPL/ITGAV/SERPINA5/SPP1/SLCO2A1/APOH |
| BP | GO:0071679 | commissural neuron axon guidance | 0.004025007 | 0.00226002 | VEGFA/NRP1 |
| BP | GO:1902667 | regulation of axon guidance | 0.004025007 | 0.00226002 | VEGFA/NRP1 |
| BP | GO:2000343 | positive regulation of chemokine (C-X-C motif) ligand 2 production | 0.004025007 | 0.00226002 | POSTN/LPL |
| BP | GO:2001236 | regulation of extrinsic apoptotic signaling pathway | 0.004025007 | 0.00226002 | PF4/FGFR1/ITGAV/NRP1 |
| BP | GO:0030199 | collagen fibril organization | 0.004341784 | 0.002437888 | COL5A2/COL3A1/LUM |
| BP | GO:0044403 | biological process involved in symbiotic interaction | 0.004341784 | 0.002437888 | PF4/ITGAV/NRP1/PGLYRP1/CXCL6 |
| BP | GO:0001764 | neuron migration | 0.004341784 | 0.002437888 | VEGFA/FGFR1/COL3A1/NRP1 |
| BP | GO:0060485 | mesenchyme development | 0.004341784 | 0.002437888 | FGFR1/JAG1/NRP1/S100A4/MSX1 |
| BP | GO:0001837 | epithelial to mesenchymal transition | 0.004371047 | 0.002454319 | FGFR1/JAG1/S100A4/MSX1 |
| BP | GO:0001822 | kidney development | 0.004371047 | 0.002454319 | VEGFA/KCNJ8/JAG1/NRP1/PDGFA |
| BP | GO:0045602 | negative regulation of endothelial cell differentiation | 0.004404704 | 0.002473218 | VEGFA/JAG1 |
| BP | GO:0033674 | positive regulation of kinase activity | 0.004413369 | 0.002478083 | VEGFA/FGFR1/CCND2/PDGFA/PTK2/VAV2 |
| BP | GO:0061564 | axon development | 0.004413369 | 0.002478083 | VEGFA/TNFRSF21/APP/SPP1/NRP1/PTK2 |
| BP | GO:0030198 | extracellular matrix organization | 0.004554627 | 0.002557398 | POSTN/COL5A2/APP/COL3A1/LUM |
| BP | GO:0038034 | signal transduction in absence of ligand | 0.004554627 | 0.002557398 | PF4/FGFR1/ITGAV |
| BP | GO:0097192 | extrinsic apoptotic signaling pathway in absence of ligand | 0.004554627 | 0.002557398 | PF4/FGFR1/ITGAV |
| BP | GO:0043062 | extracellular structure organization | 0.004554627 | 0.002557398 | POSTN/COL5A2/APP/COL3A1/LUM |
| BP | GO:0072001 | renal system development | 0.004554627 | 0.002557398 | VEGFA/KCNJ8/JAG1/NRP1/PDGFA |
| BP | GO:0007162 | negative regulation of cell adhesion | 0.004554627 | 0.002557398 | POSTN/VEGFA/TNFRSF21/JAG1/PTK2 |
| BP | GO:0045229 | external encapsulating structure organization | 0.004554627 | 0.002557398 | POSTN/COL5A2/APP/COL3A1/LUM |
| BP | GO:0051893 | regulation of focal adhesion assembly | 0.004554627 | 0.002557398 | VEGFA/NRP1/PTK2 |
| BP | GO:0090109 | regulation of cell-substrate junction assembly | 0.004554627 | 0.002557398 | VEGFA/NRP1/PTK2 |
| BP | GO:0016049 | cell growth | 0.004674328 | 0.00262461 | POSTN/VEGFA/APP/SPP1/NRP1/MSX1 |
| BP | GO:0031640 | killing of cells of other organism | 0.004870312 | 0.002734654 | PF4/PGLYRP1/CXCL6 |
| BP | GO:0007219 | Notch signaling pathway | 0.005121596 | 0.002875748 | POSTN/APP/JAG1/JAG2 |
| BP | GO:0051917 | regulation of fibrinolysis | 0.005121596 | 0.002875748 | THBD/APOH |
| BP | GO:0007584 | response to nutrient | 0.005255812 | 0.00295111 | POSTN/STC1/LPL/SPP1 |
| BP | GO:0032722 | positive regulation of chemokine production | 0.005255812 | 0.00295111 | POSTN/LPL/APP |
| BP | GO:0150116 | regulation of cell-substrate junction organization | 0.005255812 | 0.00295111 | VEGFA/NRP1/PTK2 |
| BP | GO:0033627 | cell adhesion mediated by integrin | 0.00542287 | 0.003044912 | ITGAV/PTK2/VTN |
| BP | GO:2001028 | positive regulation of endothelial cell chemotaxis | 0.005601412 | 0.003145162 | VEGFA/FGFR1 |
| BP | GO:0001936 | regulation of endothelial cell proliferation | 0.005601412 | 0.003145162 | VEGFA/FGFR1/NRP1/APOH |
| BP | GO:0010721 | negative regulation of cell development | 0.005601412 | 0.003145162 | POSTN/VEGFA/SPP1/NRP1 |
| BP | GO:0003151 | outflow tract morphogenesis | 0.005601412 | 0.003145162 | VEGFA/JAG1/NRP1 |
| BP | GO:0048844 | artery morphogenesis | 0.005601412 | 0.003145162 | VEGFA/JAG1/NRP1 |
| BP | GO:0008361 | regulation of cell size | 0.005628128 | 0.003160163 | VEGFA/SPP1/NRP1/VAV2 |
| BP | GO:0043536 | positive regulation of blood vessel endothelial cell migration | 0.005941189 | 0.003335946 | VEGFA/FGFR1/NRP1 |
| BP | GO:0072148 | epithelial cell fate commitment | 0.005941189 | 0.003335946 | NRP1/JAG2 |
| BP | GO:1901163 | regulation of trophoblast cell migration | 0.005941189 | 0.003335946 | VEGFA/TIMP1 |
| BP | GO:0007492 | endoderm development | 0.006012305 | 0.003375877 | COL5A2/ITGAV/VTN |
| BP | GO:0001655 | urogenital system development | 0.006096335 | 0.003423059 | VEGFA/KCNJ8/JAG1/NRP1/PDGFA |
| BP | GO:0014068 | positive regulation of phosphatidylinositol 3-kinase signaling | 0.006269555 | 0.003520321 | FGFR1/PDGFA/PTK2 |
| BP | GO:0061844 | antimicrobial humoral immune response mediated by antimicrobial peptide | 0.006269555 | 0.003520321 | PF4/PGLYRP1/CXCL6 |
| BP | GO:0051216 | cartilage development | 0.006269555 | 0.003520321 | STC1/MSX1/TIMP1/LUM |
| BP | GO:0060977 | coronary vasculature morphogenesis | 0.006269555 | 0.003520321 | VEGFA/NRP1 |
| BP | GO:0061450 | trophoblast cell migration | 0.006269555 | 0.003520321 | VEGFA/TIMP1 |
| BP | GO:0030336 | negative regulation of cell migration | 0.006269555 | 0.003520321 | STC1/JAG1/COL3A1/TIMP1/APOH |
| BP | GO:0001935 | endothelial cell proliferation | 0.006449255 | 0.003621222 | VEGFA/FGFR1/NRP1/APOH |
| BP | GO:0045861 | negative regulation of proteolysis | 0.006666885 | 0.00374342 | VEGFA/SERPINA5/APP/VTN/TIMP1 |
| BP | GO:0022408 | negative regulation of cell-cell adhesion | 0.006666885 | 0.00374342 | VEGFA/TNFRSF21/JAG1/PTK2 |
| BP | GO:0043393 | regulation of protein binding | 0.006666885 | 0.00374342 | LRPAP1/APP/NRP1/VTN |
| BP | GO:2001233 | regulation of apoptotic signaling pathway | 0.007029019 | 0.003946756 | PF4/FGFR1/ITGAV/NRP1/MSX1 |
| BP | GO:0071902 | positive regulation of protein serine/threonine kinase activity | 0.007077101 | 0.003973754 | VEGFA/FGFR1/CCND2/PDGFA |
| BP | GO:0032092 | positive regulation of protein binding | 0.007127757 | 0.004002197 | APP/NRP1/VTN |
| BP | GO:2000146 | negative regulation of cell motility | 0.007127757 | 0.004002197 | STC1/JAG1/COL3A1/TIMP1/APOH |
| BP | GO:0010544 | negative regulation of platelet activation | 0.007193089 | 0.004038881 | THBD/PDGFA |
| BP | GO:0010744 | positive regulation of macrophage derived foam cell differentiation | 0.007193089 | 0.004038881 | LPL/PF4 |
| BP | GO:0031290 | retinal ganglion cell axon guidance | 0.007193089 | 0.004038881 | VEGFA/NRP1 |
| BP | GO:0051098 | regulation of binding | 0.00721597 | 0.004051728 | LRPAP1/APP/NRP1/MSX1/VTN |
| BP | GO:1901888 | regulation of cell junction assembly | 0.00721597 | 0.004051728 | VEGFA/APP/NRP1/PTK2 |
| BP | GO:0048041 | focal adhesion assembly | 0.007229974 | 0.004059591 | VEGFA/NRP1/PTK2 |
| BP | GO:0051271 | negative regulation of cellular component movement | 0.007460911 | 0.004189261 | STC1/JAG1/COL3A1/TIMP1/APOH |
| BP | GO:0050900 | leukocyte migration | 0.00757179 | 0.004251519 | VEGFA/PF4/APP/PTK2/CXCL6 |
| BP | GO:0072567 | chemokine (C-X-C motif) ligand 2 production | 0.00757179 | 0.004251519 | POSTN/LPL |
| BP | GO:2000341 | regulation of chemokine (C-X-C motif) ligand 2 production | 0.00757179 | 0.004251519 | POSTN/LPL |
| BP | GO:0050866 | negative regulation of cell activation | 0.007691588 | 0.004318785 | THBD/TNFRSF21/PDGFA/PGLYRP1 |
| BP | GO:0030100 | regulation of endocytosis | 0.007772793 | 0.004364381 | LRPAP1/VEGFA/ITGAV/VTN |
| BP | GO:0060445 | branching involved in salivary gland morphogenesis | 0.008163277 | 0.004583636 | NRP1/PDGFA |
| BP | GO:0051346 | negative regulation of hydrolase activity | 0.008163277 | 0.004583636 | VEGFA/SERPINA5/APP/VTN/TIMP1 |
| BP | GO:0033273 | response to vitamin | 0.008163277 | 0.004583636 | POSTN/STC1/SPP1 |
| BP | GO:0051591 | response to cAMP | 0.008163277 | 0.004583636 | STC1/THBD/APP |
| BP | GO:0097191 | extrinsic apoptotic signaling pathway | 0.008414141 | 0.004724495 | PF4/FGFR1/ITGAV/NRP1 |
| BP | GO:0007044 | cell-substrate junction assembly | 0.008414141 | 0.004724495 | VEGFA/NRP1/PTK2 |
| BP | GO:0008593 | regulation of Notch signaling pathway | 0.008414141 | 0.004724495 | POSTN/JAG1/JAG2 |
| BP | GO:0045860 | positive regulation of protein kinase activity | 0.008414141 | 0.004724495 | VEGFA/FGFR1/CCND2/PDGFA/PTK2 |
| BP | GO:0007413 | axonal fasciculation | 0.008414141 | 0.004724495 | TNFRSF21/NRP1 |
| BP | GO:0106030 | neuron projection fasciculation | 0.008414141 | 0.004724495 | TNFRSF21/NRP1 |
| BP | GO:0097529 | myeloid leukocyte migration | 0.008414141 | 0.004724495 | VEGFA/PF4/PTK2/CXCL6 |
| BP | GO:0010769 | regulation of cell morphogenesis involved in differentiation | 0.008475316 | 0.004758844 | POSTN/NRP1/PTK2 |
| BP | GO:2001237 | negative regulation of extrinsic apoptotic signaling pathway | 0.008674914 | 0.004870917 | PF4/ITGAV/NRP1 |
| BP | GO:0050920 | regulation of chemotaxis | 0.008674914 | 0.004870917 | VEGFA/FGFR1/NRP1/PTK2 |
| BP | GO:0008037 | cell recognition | 0.008814499 | 0.004949294 | VCAN/TNFRSF21/APP/NRP1 |
| BP | GO:0030194 | positive regulation of blood coagulation | 0.008814499 | 0.004949294 | THBD/APOH |
| BP | GO:1900048 | positive regulation of hemostasis | 0.008814499 | 0.004949294 | THBD/APOH |
| BP | GO:0007411 | axon guidance | 0.008949651 | 0.005025181 | VEGFA/APP/NRP1/PTK2 |
| BP | GO:0046777 | protein autophosphorylation | 0.008949651 | 0.005025181 | VEGFA/FGFR1/PDGFA/PTK2 |
| BP | GO:0006869 | lipid transport | 0.008949651 | 0.005025181 | ITGAV/SERPINA5/SPP1/SLCO2A1/APOH |
| BP | GO:0060840 | artery development | 0.008949651 | 0.005025181 | VEGFA/JAG1/NRP1 |
| BP | GO:0097485 | neuron projection guidance | 0.008949651 | 0.005025181 | VEGFA/APP/NRP1/PTK2 |
| BP | GO:0001649 | osteoblast differentiation | 0.009022129 | 0.005065877 | VCAN/JAG1/SPP1/PTK2 |
| BP | GO:0150115 | cell-substrate junction organization | 0.009022129 | 0.005065877 | VEGFA/NRP1/PTK2 |
| BP | GO:0030595 | leukocyte chemotaxis | 0.009022129 | 0.005065877 | VEGFA/PF4/PTK2/CXCL6 |
| BP | GO:0050820 | positive regulation of coagulation | 0.009022129 | 0.005065877 | THBD/APOH |
| BP | GO:2001026 | regulation of endothelial cell chemotaxis | 0.009022129 | 0.005065877 | VEGFA/FGFR1 |
| BP | GO:0032760 | positive regulation of tumor necrosis factor production | 0.009392291 | 0.00527372 | LPL/PF4/APP |
| BP | GO:0042730 | fibrinolysis | 0.009677515 | 0.005433872 | THBD/APOH |
| BP | GO:1903557 | positive regulation of tumor necrosis factor superfamily cytokine production | 0.010286526 | 0.005775829 | LPL/PF4/APP |
| BP | GO:0046697 | decidualization | 0.010286526 | 0.005775829 | STC1/SPP1 |
| BP | GO:0048668 | collateral sprouting | 0.010286526 | 0.005775829 | APP/SPP1 |
| BP | GO:0007409 | axonogenesis | 0.010399891 | 0.005839482 | VEGFA/APP/SPP1/NRP1/PTK2 |
| BP | GO:0006898 | receptor-mediated endocytosis | 0.010702849 | 0.006009592 | LRPAP1/VEGFA/ITGAV/VTN |
| BP | GO:0061387 | regulation of extent of cell growth | 0.010703967 | 0.00601022 | VEGFA/SPP1/NRP1 |
| BP | GO:0002433 | immune response-regulating cell surface receptor signaling pathway involved in phagocytosis | 0.010703967 | 0.00601022 | PTK2/VAV2 |
| BP | GO:0031954 | positive regulation of protein autophosphorylation | 0.010703967 | 0.00601022 | VEGFA/PDGFA |
| BP | GO:0038096 | Fc-gamma receptor signaling pathway involved in phagocytosis | 0.010703967 | 0.00601022 | PTK2/VAV2 |
| BP | GO:0048608 | reproductive structure development | 0.010703967 | 0.00601022 | STC1/VEGFA/SERPINA5/SPP1/PTK2 |
| BP | GO:0001938 | positive regulation of endothelial cell proliferation | 0.01081026 | 0.006069902 | VEGFA/FGFR1/NRP1 |
| BP | GO:0014066 | regulation of phosphatidylinositol 3-kinase signaling | 0.01081026 | 0.006069902 | FGFR1/PDGFA/PTK2 |
| BP | GO:0061458 | reproductive system development | 0.010859922 | 0.006097787 | STC1/VEGFA/SERPINA5/SPP1/PTK2 |
| BP | GO:0043406 | positive regulation of MAP kinase activity | 0.010970603 | 0.006159934 | VEGFA/FGFR1/PDGFA |
| BP | GO:0038094 | Fc-gamma receptor signaling pathway | 0.011087295 | 0.006225456 | PTK2/VAV2 |
| BP | GO:0051894 | positive regulation of focal adhesion assembly | 0.011087295 | 0.006225456 | VEGFA/NRP1 |
| BP | GO:0052548 | regulation of endopeptidase activity | 0.011186056 | 0.00628091 | VEGFA/SERPINA5/APP/VTN/TIMP1 |
| BP | GO:0061448 | connective tissue development | 0.011207397 | 0.006292893 | STC1/MSX1/TIMP1/LUM |
| BP | GO:0048732 | gland development | 0.011526212 | 0.006471906 | VEGFA/SERPINA5/NRP1/PDGFA/MSX1 |
| BP | GO:0051873 | killing by host of symbiont cells | 0.011577864 | 0.006500908 | PF4/CXCL6 |
| BP | GO:0051883 | killing of cells in other organism involved in symbiotic interaction | 0.011577864 | 0.006500908 | PF4/CXCL6 |
| BP | GO:0090596 | sensory organ morphogenesis | 0.011624319 | 0.006526992 | VEGFA/COL5A2/JAG1/MSX1 |
| BP | GO:0061311 | cell surface receptor signaling pathway involved in heart development | 0.012259461 | 0.006883621 | JAG1/MSX1 |
| BP | GO:0051897 | positive regulation of protein kinase B signaling | 0.012633425 | 0.0070936 | FGFR1/PDGFA/PTK2 |
| BP | GO:0043588 | skin development | 0.012633425 | 0.0070936 | COL5A2/JAG1/COL3A1/PDGFA |
| BP | GO:0001704 | formation of primary germ layer | 0.01284661 | 0.007213302 | COL5A2/ITGAV/VTN |
| BP | GO:0019730 | antimicrobial humoral response | 0.01308536 | 0.007347359 | PF4/PGLYRP1/CXCL6 |
| BP | GO:0009314 | response to radiation | 0.013381453 | 0.007513614 | THBD/CCND2/APP/COL3A1/TIMP1 |
| BP | GO:0007435 | salivary gland morphogenesis | 0.013381453 | 0.007513614 | NRP1/PDGFA |
| BP | GO:0048841 | regulation of axon extension involved in axon guidance | 0.013381453 | 0.007513614 | VEGFA/NRP1 |
| BP | GO:0060317 | cardiac epithelial to mesenchymal transition | 0.013381453 | 0.007513614 | JAG1/MSX1 |
| BP | GO:0071621 | granulocyte chemotaxis | 0.013606738 | 0.00764011 | PF4/PTK2/CXCL6 |
| BP | GO:2000027 | regulation of animal organ morphogenesis | 0.013606738 | 0.00764011 | VEGFA/PDGFA/MSX1 |
| BP | GO:0052547 | regulation of peptidase activity | 0.013633532 | 0.007655154 | VEGFA/SERPINA5/APP/VTN/TIMP1 |
| BP | GO:0002431 | Fc receptor mediated stimulatory signaling pathway | 0.013741764 | 0.007715926 | PTK2/VAV2 |
| BP | GO:0010165 | response to X-ray | 0.013741764 | 0.007715926 | THBD/CCND2 |
| BP | GO:0043552 | positive regulation of phosphatidylinositol 3-kinase activity | 0.013741764 | 0.007715926 | PTK2/VAV2 |
| BP | GO:0150117 | positive regulation of cell-substrate junction organization | 0.013741764 | 0.007715926 | VEGFA/NRP1 |
| BP | GO:0001819 | positive regulation of cytokine production | 0.014077847 | 0.007904635 | POSTN/LPL/PF4/APP/LUM |
| BP | GO:0007431 | salivary gland development | 0.014372676 | 0.00807018 | NRP1/PDGFA |
| BP | GO:0048261 | negative regulation of receptor-mediated endocytosis | 0.014372676 | 0.00807018 | LRPAP1/ITGAV |
| BP | GO:0046683 | response to organophosphorus | 0.014867955 | 0.008348276 | STC1/THBD/APP |
| BP | GO:0001569 | branching involved in blood vessel morphogenesis | 0.014867955 | 0.008348276 | VEGFA/NRP1 |
| BP | GO:0001893 | maternal placenta development | 0.014867955 | 0.008348276 | STC1/SPP1 |
| BP | GO:0033280 | response to vitamin D | 0.014867955 | 0.008348276 | STC1/SPP1 |
| BP | GO:0090050 | positive regulation of cell migration involved in sprouting angiogenesis | 0.014867955 | 0.008348276 | VEGFA/NRP1 |
| BP | GO:0010595 | positive regulation of endothelial cell migration | 0.015179642 | 0.008523287 | VEGFA/FGFR1/NRP1 |
| BP | GO:0043410 | positive regulation of MAPK cascade | 0.015206696 | 0.008538478 | VEGFA/FGFR1/APP/NRP1/PDGFA |
| BP | GO:0002687 | positive regulation of leukocyte migration | 0.015691809 | 0.008810866 | VEGFA/APP/PTK2 |
| BP | GO:0048846 | axon extension involved in axon guidance | 0.016147142 | 0.009066533 | VEGFA/NRP1 |
| BP | GO:0090218 | positive regulation of lipid kinase activity | 0.016147142 | 0.009066533 | PTK2/VAV2 |
| BP | GO:1902284 | neuron projection extension involved in neuron projection guidance | 0.016147142 | 0.009066533 | VEGFA/NRP1 |
| BP | GO:0042491 | inner ear auditory receptor cell differentiation | 0.01694627 | 0.009515239 | JAG1/JAG2 |
| BP | GO:0010463 | mesenchymal cell proliferation | 0.017760018 | 0.009972154 | PDGFA/MSX1 |
| BP | GO:0051056 | regulation of small GTPase mediated signal transduction | 0.018067159 | 0.010144612 | ITGAV/COL3A1/NRP1/VAV2 |
| BP | GO:0001890 | placenta development | 0.018173489 | 0.010204316 | STC1/SPP1/PTK2 |
| BP | GO:0014065 | phosphatidylinositol 3-kinase signaling | 0.018173489 | 0.010204316 | FGFR1/PDGFA/PTK2 |
| BP | GO:0090287 | regulation of cellular response to growth factor stimulus | 0.018255818 | 0.010250543 | FSTL1/FGFR1/MSX1/VTN |
| BP | GO:0010543 | regulation of platelet activation | 0.018259113 | 0.010252393 | THBD/PDGFA |
| BP | GO:0001953 | negative regulation of cell-matrix adhesion | 0.019004158 | 0.010670732 | POSTN/JAG1 |
| BP | GO:0071634 | regulation of transforming growth factor beta production | 0.019004158 | 0.010670732 | ITGAV/LUM |
| BP | GO:0022604 | regulation of cell morphogenesis | 0.019022643 | 0.010681111 | POSTN/VEGFA/NRP1/PTK2 |
| BP | GO:0014074 | response to purine-containing compound | 0.019043072 | 0.010692582 | STC1/THBD/APP |
| BP | GO:0097530 | granulocyte migration | 0.019043072 | 0.010692582 | PF4/PTK2/CXCL6 |
| BP | GO:0045773 | positive regulation of axon extension | 0.019586686 | 0.010997818 | VEGFA/NRP1 |
| BP | GO:0043535 | regulation of blood vessel endothelial cell migration | 0.019895013 | 0.011170942 | VEGFA/FGFR1/NRP1 |
| BP | GO:0048592 | eye morphogenesis | 0.019895013 | 0.011170942 | VEGFA/COL5A2/JAG1 |
| BP | GO:0031952 | regulation of protein autophosphorylation | 0.020063962 | 0.011265806 | VEGFA/PDGFA |
| BP | GO:0071604 | transforming growth factor beta production | 0.020063962 | 0.011265806 | ITGAV/LUM |
| BP | GO:2001239 | regulation of extrinsic apoptotic signaling pathway in absence of ligand | 0.020063962 | 0.011265806 | PF4/FGFR1 |
| BP | GO:0006959 | humoral immune response | 0.020063962 | 0.011265806 | PF4/TNFRSF21/PGLYRP1/CXCL6 |
| BP | GO:0050770 | regulation of axonogenesis | 0.020589805 | 0.011561064 | VEGFA/SPP1/NRP1 |
| BP | GO:0003197 | endocardial cushion development | 0.021512678 | 0.012079252 | JAG1/MSX1 |
| BP | GO:0031670 | cellular response to nutrient | 0.021512678 | 0.012079252 | POSTN/LPL |
| BP | GO:0035272 | exocrine system development | 0.021512678 | 0.012079252 | NRP1/PDGFA |
| BP | GO:0050919 | negative chemotaxis | 0.022279302 | 0.012509707 | ITGAV/PDGFA |
| BP | GO:0060976 | coronary vasculature development | 0.022279302 | 0.012509707 | VEGFA/NRP1 |
| BP | GO:0048638 | regulation of developmental growth | 0.022413956 | 0.012585315 | VEGFA/APP/SPP1/NRP1 |
| BP | GO:0070371 | ERK1 and ERK2 cascade | 0.022413956 | 0.012585315 | ITGAV/APP/NRP1/PDGFA |
| BP | GO:0035315 | hair cell differentiation | 0.022774763 | 0.012787906 | JAG1/JAG2 |
| BP | GO:0045601 | regulation of endothelial cell differentiation | 0.022774763 | 0.012787906 | VEGFA/JAG1 |
| BP | GO:0045747 | positive regulation of Notch signaling pathway | 0.022774763 | 0.012787906 | JAG1/JAG2 |
| BP | GO:0030857 | negative regulation of epithelial cell differentiation | 0.024519649 | 0.01376765 | VEGFA/JAG1 |
| BP | GO:0038084 | vascular endothelial growth factor signaling pathway | 0.024519649 | 0.01376765 | VEGFA/NRP1 |
| BP | GO:0045765 | regulation of angiogenesis | 0.024832798 | 0.013943481 | VEGFA/PF4/NRP1/APOH |
| BP | GO:2000241 | regulation of reproductive process | 0.024832798 | 0.013943481 | VEGFA/MSX1/TIMP1 |
| BP | GO:0032496 | response to lipopolysaccharide | 0.024909168 | 0.013986363 | PF4/THBD/KCNJ8/CXCL6 |
| BP | GO:0038093 | Fc receptor signaling pathway | 0.025012288 | 0.014044264 | PTK2/VAV2 |
| BP | GO:0060688 | regulation of morphogenesis of a branching structure | 0.025012288 | 0.014044264 | VEGFA/PDGFA |
| BP | GO:1901342 | regulation of vasculature development | 0.025915279 | 0.014551289 | VEGFA/PF4/NRP1/APOH |
| BP | GO:1990138 | neuron projection extension | 0.026006781 | 0.014602666 | POSTN/VEGFA/NRP1 |
| BP | GO:0010469 | regulation of signaling receptor activity | 0.026227707 | 0.014726716 | LRPAP1/APP/NRP1 |
| BP | GO:0051099 | positive regulation of binding | 0.026227707 | 0.014726716 | APP/NRP1/VTN |
| BP | GO:0048260 | positive regulation of receptor-mediated endocytosis | 0.026491941 | 0.014875082 | VEGFA/VTN |
| BP | GO:0048771 | tissue remodeling | 0.026875442 | 0.015090415 | JAG1/SPP1/TIMP1 |
| BP | GO:0007178 | transmembrane receptor protein serine/threonine kinase signaling pathway | 0.02687977 | 0.015092846 | FSTL1/COL3A1/PTK2/MSX1 |
| BP | GO:0010634 | positive regulation of epithelial cell migration | 0.02687977 | 0.015092846 | VEGFA/FGFR1/NRP1 |
| BP | GO:0043534 | blood vessel endothelial cell migration | 0.02687977 | 0.015092846 | VEGFA/FGFR1/NRP1 |
| BP | GO:0045806 | negative regulation of endocytosis | 0.02687977 | 0.015092846 | LRPAP1/ITGAV |
| BP | GO:0071320 | cellular response to cAMP | 0.02687977 | 0.015092846 | STC1/APP |
| BP | GO:0043405 | regulation of MAP kinase activity | 0.027118977 | 0.015227159 | VEGFA/FGFR1/PDGFA |
| BP | GO:0048015 | phosphatidylinositol-mediated signaling | 0.027445309 | 0.015410392 | FGFR1/PDGFA/PTK2 |
| BP | GO:0071900 | regulation of protein serine/threonine kinase activity | 0.02753713 | 0.01546195 | VEGFA/FGFR1/CCND2/PDGFA |
| BP | GO:0002237 | response to molecule of bacterial origin | 0.028226644 | 0.015849108 | PF4/THBD/KCNJ8/CXCL6 |
| BP | GO:0032640 | tumor necrosis factor production | 0.028226644 | 0.015849108 | LPL/PF4/APP |
| BP | GO:0032680 | regulation of tumor necrosis factor production | 0.028226644 | 0.015849108 | LPL/PF4/APP |
| BP | GO:1905952 | regulation of lipid localization | 0.028226644 | 0.015849108 | LPL/ITGAV/SPP1 |
| BP | GO:0048017 | inositol lipid-mediated signaling | 0.028454408 | 0.015976996 | FGFR1/PDGFA/PTK2 |
| BP | GO:0061138 | morphogenesis of a branching epithelium | 0.028454408 | 0.015976996 | VEGFA/NRP1/PDGFA |
| BP | GO:0002090 | regulation of receptor internalization | 0.028850902 | 0.016199625 | LRPAP1/VEGFA |
| BP | GO:0007369 | gastrulation | 0.029455351 | 0.01653902 | COL5A2/ITGAV/VTN |
| BP | GO:0051896 | regulation of protein kinase B signaling | 0.029455351 | 0.01653902 | FGFR1/PDGFA/PTK2 |
| BP | GO:0010883 | regulation of lipid storage | 0.029495983 | 0.016561835 | LPL/ITGAV |
| BP | GO:0071706 | tumor necrosis factor superfamily cytokine production | 0.029495983 | 0.016561835 | LPL/PF4/APP |
| BP | GO:1903555 | regulation of tumor necrosis factor superfamily cytokine production | 0.029495983 | 0.016561835 | LPL/PF4/APP |
| BP | GO:0001654 | eye development | 0.029495983 | 0.016561835 | VEGFA/COL5A2/JAG1/NRP1 |
| BP | GO:0001954 | positive regulation of cell-matrix adhesion | 0.030044571 | 0.016869864 | VEGFA/NRP1 |
| BP | GO:0001906 | cell killing | 0.030044571 | 0.016869864 | PF4/PGLYRP1/CXCL6 |
| BP | GO:0030308 | negative regulation of cell growth | 0.030044571 | 0.016869864 | SPP1/NRP1/MSX1 |
| BP | GO:0150063 | visual system development | 0.030200229 | 0.016957265 | VEGFA/COL5A2/JAG1/NRP1 |
| BP | GO:0043551 | regulation of phosphatidylinositol 3-kinase activity | 0.030719021 | 0.017248564 | PTK2/VAV2 |
| BP | GO:0030900 | forebrain development | 0.031127059 | 0.017477675 | APP/COL3A1/NRP1/MSX1 |
| BP | GO:0030099 | myeloid cell differentiation | 0.031379907 | 0.017619648 | VEGFA/PF4/APP/JAG1 |
| BP | GO:0048880 | sensory system development | 0.031379907 | 0.017619648 | VEGFA/COL5A2/JAG1/NRP1 |
| BP | GO:0050678 | regulation of epithelial cell proliferation | 0.031379907 | 0.017619648 | VEGFA/FGFR1/NRP1/APOH |
| BP | GO:0050731 | positive regulation of peptidyl-tyrosine phosphorylation | 0.03153535 | 0.017706928 | VEGFA/NRP1/VTN |
| BP | GO:0032535 | regulation of cellular component size | 0.031742239 | 0.017823095 | VEGFA/SPP1/NRP1/VAV2 |
| BP | GO:0001763 | morphogenesis of a branching structure | 0.032573669 | 0.018289939 | VEGFA/NRP1/PDGFA |
| BP | GO:0045664 | regulation of neuron differentiation | 0.032573669 | 0.018289939 | FGFR1/APP/JAG1 |
| BP | GO:0032731 | positive regulation of interleukin-1 beta production | 0.032583829 | 0.018295644 | LPL/APP |
| BP | GO:0060113 | inner ear receptor cell differentiation | 0.032583829 | 0.018295644 | JAG1/JAG2 |
| BP | GO:0060135 | maternal process involved in female pregnancy | 0.032583829 | 0.018295644 | STC1/SPP1 |
| BP | GO:0001885 | endothelial cell development | 0.034423953 | 0.019328863 | STC1/VEGFA |
| BP | GO:0050771 | negative regulation of axonogenesis | 0.034423953 | 0.019328863 | SPP1/NRP1 |
| BP | GO:0051701 | biological process involved in interaction with host | 0.035165733 | 0.019745369 | ITGAV/NRP1/PGLYRP1 |
| BP | GO:1905330 | regulation of morphogenesis of an epithelium | 0.035239925 | 0.019787027 | VEGFA/PDGFA |
| BP | GO:0050918 | positive chemotaxis | 0.036178087 | 0.0203138 | VEGFA/NRP1 |
| BP | GO:0048863 | stem cell differentiation | 0.036251677 | 0.02035512 | JAG1/NRP1/MSX1 |
| BP | GO:0050679 | positive regulation of epithelial cell proliferation | 0.036616593 | 0.020560019 | VEGFA/FGFR1/NRP1 |
| BP | GO:0042490 | mechanoreceptor differentiation | 0.036862891 | 0.020698313 | JAG1/JAG2 |
| BP | GO:0002573 | myeloid leukocyte differentiation | 0.036862891 | 0.020698313 | VEGFA/PF4/APP |
| BP | GO:0010812 | negative regulation of cell-substrate adhesion | 0.037477479 | 0.021043401 | POSTN/JAG1 |
| BP | GO:0060411 | cardiac septum morphogenesis | 0.037477479 | 0.021043401 | JAG1/NRP1 |
| BP | GO:0002685 | regulation of leukocyte migration | 0.037477479 | 0.021043401 | VEGFA/APP/PTK2 |
| BP | GO:0043491 | protein kinase B signaling | 0.037846581 | 0.02125065 | FGFR1/PDGFA/PTK2 |
| BP | GO:0045665 | negative regulation of neuron differentiation | 0.038170114 | 0.021432312 | APP/JAG1 |
| BP | GO:0060193 | positive regulation of lipase activity | 0.038170114 | 0.021432312 | FGFR1/APOH |
| BP | GO:0043583 | ear development | 0.038466564 | 0.021598768 | JAG1/MSX1/JAG2 |
| BP | GO:0001558 | regulation of cell growth | 0.038700987 | 0.021730395 | VEGFA/SPP1/NRP1/MSX1 |
| BP | GO:0009612 | response to mechanical stimulus | 0.039702384 | 0.022292674 | POSTN/COL3A1/PTK2 |
| BP | GO:0008344 | adult locomotory behavior | 0.039702384 | 0.022292674 | CCND2/APP |
| BP | GO:0043550 | regulation of lipid kinase activity | 0.039702384 | 0.022292674 | PTK2/VAV2 |
| BP | GO:0070374 | positive regulation of ERK1 and ERK2 cascade | 0.039840431 | 0.022370186 | APP/NRP1/PDGFA |
| BP | GO:0034329 | cell junction assembly | 0.040036031 | 0.022480015 | VEGFA/APP/NRP1/PTK2 |
| BP | GO:0043903 | regulation of biological process involved in symbiotic interaction | 0.040282571 | 0.022618446 | ITGAV/CXCL6 |
| BP | GO:0051057 | positive regulation of small GTPase mediated signal transduction | 0.040282571 | 0.022618446 | ITGAV/COL3A1 |
| BP | GO:0002064 | epithelial cell development | 0.040724601 | 0.022866643 | STC1/VEGFA/JAG1 |
| BP | GO:0048705 | skeletal system morphogenesis | 0.040724601 | 0.022866643 | STC1/FGFR1/MSX1 |
| BP | GO:0032732 | positive regulation of interleukin-1 production | 0.040736941 | 0.022873572 | LPL/APP |
| BP | GO:0035924 | cellular response to vascular endothelial growth factor stimulus | 0.040736941 | 0.022873572 | VEGFA/NRP1 |
| BP | GO:0086003 | cardiac muscle cell contraction | 0.040736941 | 0.022873572 | STC1/KCNJ8 |
| BP | GO:2001234 | negative regulation of apoptotic signaling pathway | 0.042252632 | 0.023724624 | PF4/ITGAV/NRP1 |
| BP | GO:0021536 | diencephalon development | 0.043752443 | 0.02456676 | NRP1/MSX1 |
| BP | GO:0050673 | epithelial cell proliferation | 0.044491204 | 0.02498157 | VEGFA/FGFR1/NRP1/APOH |
| BP | GO:0090049 | regulation of cell migration involved in sprouting angiogenesis | 0.045713584 | 0.02566793 | VEGFA/NRP1 |
| BP | GO:0051960 | regulation of nervous system development | 0.04633306 | 0.026015763 | VEGFA/TNFRSF21/SPP1/NRP1 |
| BP | GO:0050772 | positive regulation of axonogenesis | 0.046564148 | 0.026145517 | VEGFA/NRP1 |
| BP | GO:0001570 | vasculogenesis | 0.047416851 | 0.026624305 | VEGFA/ITGAV |
| BP | GO:0071229 | cellular response to acid chemical | 0.047416851 | 0.026624305 | VEGFA/COL5A2 |
| BP | GO:0090068 | positive regulation of cell cycle process | 0.047427949 | 0.026630536 | CCND2/APP/MSX1 |
| BP | GO:0022407 | regulation of cell-cell adhesion | 0.047427949 | 0.026630536 | VEGFA/TNFRSF21/JAG1/PTK2 |
| BP | GO:0014032 | neural crest cell development | 0.047987655 | 0.026944809 | JAG1/NRP1 |
| BP | GO:2000243 | positive regulation of reproductive process | 0.047987655 | 0.026944809 | VEGFA/MSX1 |
| BP | GO:0060021 | roof of mouth development | 0.05222131 | 0.029321983 | MSX1/JAG2 |
| BP | GO:0097006 | regulation of plasma lipoprotein particle levels | 0.05222131 | 0.029321983 | LRPAP1/LPL |
| BP | GO:0051402 | neuron apoptotic process | 0.05222131 | 0.029321983 | TNFRSF21/APP/NRP1 |
| BP | GO:0014031 | mesenchymal cell development | 0.052877181 | 0.029690251 | JAG1/NRP1 |
| BP | GO:0035023 | regulation of Rho protein signal transduction | 0.052877181 | 0.029690251 | COL3A1/NRP1 |
| BP | GO:0048864 | stem cell development | 0.052877181 | 0.029690251 | JAG1/NRP1 |
| BP | GO:0045926 | negative regulation of growth | 0.053329435 | 0.029944189 | SPP1/NRP1/MSX1 |
| BP | GO:0019915 | lipid storage | 0.053597766 | 0.030094855 | LPL/ITGAV |
| BP | GO:0071560 | cellular response to transforming growth factor beta stimulus | 0.053597766 | 0.030094855 | POSTN/COL3A1/PTK2 |
| BP | GO:0070098 | chemokine-mediated signaling pathway | 0.054615857 | 0.030666508 | PF4/CXCL6 |
| BP | GO:0031667 | response to nutrient levels | 0.055289912 | 0.031044986 | POSTN/STC1/LPL/SPP1 |
| BP | GO:0051781 | positive regulation of cell division | 0.055487341 | 0.031155841 | VEGFA/PDGFA |
| BP | GO:0071559 | response to transforming growth factor beta | 0.056360162 | 0.031645926 | POSTN/COL3A1/PTK2 |
| BP | GO:0042475 | odontogenesis of dentin-containing tooth | 0.056360162 | 0.031645926 | MSX1/JAG2 |
| BP | GO:0045165 | cell fate commitment | 0.056755974 | 0.031868172 | JAG1/NRP1/JAG2 |
| BP | GO:0014033 | neural crest cell differentiation | 0.056755974 | 0.031868172 | JAG1/NRP1 |
| BP | GO:0055006 | cardiac cell development | 0.056755974 | 0.031868172 | VEGFA/JAG1 |
| BP | GO:0060191 | regulation of lipase activity | 0.056755974 | 0.031868172 | FGFR1/APOH |
| BP | GO:0072080 | nephron tubule development | 0.056755974 | 0.031868172 | VEGFA/JAG1 |
| BP | GO:0032755 | positive regulation of interleukin-6 production | 0.058660101 | 0.032937329 | LPL/APP |
| BP | GO:0060349 | bone morphogenesis | 0.058660101 | 0.032937329 | STC1/MSX1 |
| BP | GO:1901992 | positive regulation of mitotic cell cycle phase transition | 0.058660101 | 0.032937329 | CCND2/APP |
| BP | GO:0002042 | cell migration involved in sprouting angiogenesis | 0.059209262 | 0.03324568 | VEGFA/NRP1 |
| BP | GO:0002690 | positive regulation of leukocyte chemotaxis | 0.059209262 | 0.03324568 | VEGFA/PTK2 |
| BP | GO:0051702 | biological process involved in interaction with symbiont | 0.059209262 | 0.03324568 | PF4/CXCL6 |
| BP | GO:0061326 | renal tubule development | 0.059209262 | 0.03324568 | VEGFA/JAG1 |
| BP | GO:0030516 | regulation of axon extension | 0.060245514 | 0.03382753 | VEGFA/NRP1 |
| BP | GO:0030510 | regulation of BMP signaling pathway | 0.061120549 | 0.034318857 | FSTL1/MSX1 |
| BP | GO:1901655 | cellular response to ketone | 0.061120549 | 0.034318857 | POSTN/SPP1 |
| BP | GO:0010596 | negative regulation of endothelial cell migration | 0.061664104 | 0.034624061 | STC1/APOH |
| BP | GO:0070252 | actin-mediated cell contraction | 0.061664104 | 0.034624061 | STC1/KCNJ8 |
| BP | GO:1990868 | response to chemokine | 0.061664104 | 0.034624061 | PF4/CXCL6 |
| BP | GO:1990869 | cellular response to chemokine | 0.061664104 | 0.034624061 | PF4/CXCL6 |
| BP | GO:0048872 | homeostasis of number of cells | 0.062652279 | 0.035178916 | FSTL1/VEGFA/CXCL6 |
| BP | GO:0006641 | triglyceride metabolic process | 0.064801425 | 0.036385649 | LPL/APOH |
| BP | GO:0045807 | positive regulation of endocytosis | 0.064801425 | 0.036385649 | VEGFA/VTN |
| BP | GO:0003279 | cardiac septum development | 0.067979015 | 0.038169849 | JAG1/NRP1 |
| BP | GO:0030593 | neutrophil chemotaxis | 0.067979015 | 0.038169849 | PF4/CXCL6 |
| BP | GO:0045639 | positive regulation of myeloid cell differentiation | 0.067979015 | 0.038169849 | PF4/JAG1 |
| BP | GO:1901890 | positive regulation of cell junction assembly | 0.069046806 | 0.038769408 | VEGFA/NRP1 |
| BP | GO:0002429 | immune response-activating cell surface receptor signaling pathway | 0.072014234 | 0.040435602 | TNFRSF21/PTK2/VAV2 |
| BP | GO:0002757 | immune response-activating signal transduction | 0.072014234 | 0.040435602 | TNFRSF21/PTK2/VAV2 |
| BP | GO:0033138 | positive regulation of peptidyl-serine phosphorylation | 0.072014234 | 0.040435602 | VEGFA/APP |
| BP | GO:0072009 | nephron epithelium development | 0.072014234 | 0.040435602 | VEGFA/JAG1 |
| BP | GO:0032611 | interleukin-1 beta production | 0.072014234 | 0.040435602 | LPL/APP |
| BP | GO:0032651 | regulation of interleukin-1 beta production | 0.072014234 | 0.040435602 | LPL/APP |
| BP | GO:1905954 | positive regulation of lipid localization | 0.072014234 | 0.040435602 | LPL/SPP1 |
| BP | GO:0021562 | vestibulocochlear nerve development | 0.072014234 | 0.040435602 | NRP1 |
| BP | GO:0031642 | negative regulation of myelination | 0.072014234 | 0.040435602 | TNFRSF21 |
| BP | GO:0035768 | endothelial cell chemotaxis to fibroblast growth factor | 0.072014234 | 0.040435602 | FGFR1 |
| BP | GO:0036363 | transforming growth factor beta activation | 0.072014234 | 0.040435602 | ITGAV |
| BP | GO:0038007 | netrin-activated signaling pathway | 0.072014234 | 0.040435602 | PTK2 |
| BP | GO:0048671 | negative regulation of collateral sprouting | 0.072014234 | 0.040435602 | SPP1 |
| BP | GO:0051006 | positive regulation of lipoprotein lipase activity | 0.072014234 | 0.040435602 | APOH |
| BP | GO:0060947 | cardiac vascular smooth muscle cell differentiation | 0.072014234 | 0.040435602 | VEGFA |
| BP | GO:0061299 | retina vasculature morphogenesis in camera-type eye | 0.072014234 | 0.040435602 | NRP1 |
| BP | GO:0072070 | loop of Henle development | 0.072014234 | 0.040435602 | JAG1 |
| BP | GO:0072378 | blood coagulation, fibrin clot formation | 0.072014234 | 0.040435602 | APOH |
| BP | GO:0090037 | positive regulation of protein kinase C signaling | 0.072014234 | 0.040435602 | VEGFA |
| BP | GO:0090647 | modulation of age-related behavioral decline | 0.072014234 | 0.040435602 | APP |
| BP | GO:0097084 | vascular associated smooth muscle cell development | 0.072014234 | 0.040435602 | VEGFA |
| BP | GO:0140059 | dendrite arborization | 0.072014234 | 0.040435602 | NRP1 |
| BP | GO:1901725 | regulation of histone deacetylase activity | 0.072014234 | 0.040435602 | VEGFA |
| BP | GO:1902946 | protein localization to early endosome | 0.072014234 | 0.040435602 | NRP1 |
| BP | GO:1990535 | neuron projection maintenance | 0.072014234 | 0.040435602 | APP |
| BP | GO:2000048 | negative regulation of cell-cell adhesion mediated by cadherin | 0.072014234 | 0.040435602 | VEGFA |
| BP | GO:2000544 | regulation of endothelial cell chemotaxis to fibroblast growth factor | 0.072014234 | 0.040435602 | FGFR1 |
| BP | GO:2001223 | negative regulation of neuron migration | 0.072014234 | 0.040435602 | COL3A1 |
| BP | GO:0010633 | negative regulation of epithelial cell migration | 0.073562349 | 0.041304861 | STC1/APOH |
| BP | GO:0048640 | negative regulation of developmental growth | 0.073562349 | 0.041304861 | SPP1/NRP1 |
| BP | GO:0031623 | receptor internalization | 0.074435173 | 0.041794947 | LRPAP1/VEGFA |
| BP | GO:0044344 | cellular response to fibroblast growth factor stimulus | 0.074435173 | 0.041794947 | POSTN/FGFR1 |
| BP | GO:0071675 | regulation of mononuclear cell migration | 0.07474503 | 0.04196893 | APP/PTK2 |
| BP | GO:1901989 | positive regulation of cell cycle phase transition | 0.07474503 | 0.04196893 | CCND2/APP |
| BP | GO:0001666 | response to hypoxia | 0.07474503 | 0.04196893 | POSTN/STC1/VEGFA |
| BP | GO:0001967 | suckling behavior | 0.07474503 | 0.04196893 | APP |
| BP | GO:0021561 | facial nerve development | 0.07474503 | 0.04196893 | NRP1 |
| BP | GO:0021604 | cranial nerve structural organization | 0.07474503 | 0.04196893 | NRP1 |
| BP | GO:0021610 | facial nerve morphogenesis | 0.07474503 | 0.04196893 | NRP1 |
| BP | GO:0033033 | negative regulation of myeloid cell apoptotic process | 0.07474503 | 0.04196893 | APOH |
| BP | GO:0033690 | positive regulation of osteoblast proliferation | 0.07474503 | 0.04196893 | ITGAV |
| BP | GO:0035766 | cell chemotaxis to fibroblast growth factor | 0.07474503 | 0.04196893 | FGFR1 |
| BP | GO:0042447 | hormone catabolic process | 0.07474503 | 0.04196893 | SPP1 |
| BP | GO:0060837 | blood vessel endothelial cell differentiation | 0.07474503 | 0.04196893 | NRP1 |
| BP | GO:0061314 | Notch signaling involved in heart development | 0.07474503 | 0.04196893 | JAG1 |
| BP | GO:0061365 | positive regulation of triglyceride lipase activity | 0.07474503 | 0.04196893 | APOH |
| BP | GO:0070944 | neutrophil-mediated killing of bacterium | 0.07474503 | 0.04196893 | CXCL6 |
| BP | GO:0071492 | cellular response to UV-A | 0.07474503 | 0.04196893 | TIMP1 |
| BP | GO:0072017 | distal tubule development | 0.07474503 | 0.04196893 | JAG1 |
| BP | GO:1900222 | negative regulation of amyloid-beta clearance | 0.07474503 | 0.04196893 | LRPAP1 |
| BP | GO:1904847 | regulation of cell chemotaxis to fibroblast growth factor | 0.07474503 | 0.04196893 | FGFR1 |
| BP | GO:2001053 | regulation of mesenchymal cell apoptotic process | 0.07474503 | 0.04196893 | MSX1 |
| BP | GO:0006909 | phagocytosis | 0.075067257 | 0.042149858 | ITGAV/PTK2/VAV2 |
| BP | GO:0070372 | regulation of ERK1 and ERK2 cascade | 0.075530144 | 0.042409767 | APP/NRP1/PDGFA |
| BP | GO:0022612 | gland morphogenesis | 0.076447104 | 0.042924635 | NRP1/PDGFA |
| BP | GO:0045787 | positive regulation of cell cycle | 0.076672024 | 0.043050926 | CCND2/APP/MSX1 |
| BP | GO:0048593 | camera-type eye morphogenesis | 0.076672024 | 0.043050926 | VEGFA/JAG1 |
| BP | GO:0048675 | axon extension | 0.076672024 | 0.043050926 | VEGFA/NRP1 |
| BP | GO:0071774 | response to fibroblast growth factor | 0.076672024 | 0.043050926 | POSTN/FGFR1 |
| BP | GO:0002768 | immune response-regulating cell surface receptor signaling pathway | 0.076672024 | 0.043050926 | TNFRSF21/PTK2/VAV2 |
| BP | GO:0003206 | cardiac chamber morphogenesis | 0.076672024 | 0.043050926 | JAG1/NRP1 |
| BP | GO:0045931 | positive regulation of mitotic cell cycle | 0.076672024 | 0.043050926 | CCND2/APP |
| BP | GO:0021559 | trigeminal nerve development | 0.076672024 | 0.043050926 | NRP1 |
| BP | GO:0032823 | regulation of natural killer cell differentiation | 0.076672024 | 0.043050926 | PGLYRP1 |
| BP | GO:0032908 | regulation of transforming growth factor beta1 production | 0.076672024 | 0.043050926 | LUM |
| BP | GO:0034370 | triglyceride-rich lipoprotein particle remodeling | 0.076672024 | 0.043050926 | LPL |
| BP | GO:0034372 | very-low-density lipoprotein particle remodeling | 0.076672024 | 0.043050926 | LPL |
| BP | GO:0034392 | negative regulation of smooth muscle cell apoptotic process | 0.076672024 | 0.043050926 | APOH |
| BP | GO:0043129 | surfactant homeostasis | 0.076672024 | 0.043050926 | VEGFA |
| BP | GO:0061307 | cardiac neural crest cell differentiation involved in heart development | 0.076672024 | 0.043050926 | JAG1 |
| BP | GO:0061308 | cardiac neural crest cell development involved in heart development | 0.076672024 | 0.043050926 | JAG1 |
| BP | GO:0061517 | macrophage proliferation | 0.076672024 | 0.043050926 | PTK2 |
| BP | GO:0070943 | neutrophil-mediated killing of symbiont cell | 0.076672024 | 0.043050926 | CXCL6 |
| BP | GO:0071287 | cellular response to manganese ion | 0.076672024 | 0.043050926 | APP |
| BP | GO:0072015 | glomerular visceral epithelial cell development | 0.076672024 | 0.043050926 | JAG1 |
| BP | GO:0072376 | protein activation cascade | 0.076672024 | 0.043050926 | APOH |
| BP | GO:0097152 | mesenchymal cell apoptotic process | 0.076672024 | 0.043050926 | MSX1 |
| BP | GO:0150065 | regulation of deacetylase activity | 0.076672024 | 0.043050926 | VEGFA |
| BP | GO:1902287 | semaphorin-plexin signaling pathway involved in axon guidance | 0.076672024 | 0.043050926 | NRP1 |
| BP | GO:0002688 | regulation of leukocyte chemotaxis | 0.076762764 | 0.043101876 | VEGFA/PTK2 |
| BP | GO:1990266 | neutrophil migration | 0.076762764 | 0.043101876 | PF4/CXCL6 |
| BP | GO:0010811 | positive regulation of cell-substrate adhesion | 0.0777697 | 0.043667266 | VEGFA/NRP1 |
| BP | GO:0036293 | response to decreased oxygen levels | 0.078633488 | 0.044152278 | POSTN/STC1/VEGFA |
| BP | GO:0043010 | camera-type eye development | 0.078633488 | 0.044152278 | VEGFA/JAG1/NRP1 |
| BP | GO:0008544 | epidermis development | 0.078947748 | 0.044328733 | JAG1/PDGFA/JAG2 |
| BP | GO:0032874 | positive regulation of stress-activated MAPK cascade | 0.078947748 | 0.044328733 | VEGFA/APP |
| BP | GO:0001886 | endothelial cell morphogenesis | 0.078947748 | 0.044328733 | STC1 |
| BP | GO:0002551 | mast cell chemotaxis | 0.078947748 | 0.044328733 | VEGFA |
| BP | GO:0010745 | negative regulation of macrophage derived foam cell differentiation | 0.078947748 | 0.044328733 | ITGAV |
| BP | GO:0016322 | neuron remodeling | 0.078947748 | 0.044328733 | APP |
| BP | GO:0032905 | transforming growth factor beta1 production | 0.078947748 | 0.044328733 | LUM |
| BP | GO:0042492 | gamma-delta T cell differentiation | 0.078947748 | 0.044328733 | JAG2 |
| BP | GO:0048012 | hepatocyte growth factor receptor signaling pathway | 0.078947748 | 0.044328733 | NRP1 |
| BP | GO:0051709 | regulation of killing of cells of other organism | 0.078947748 | 0.044328733 | CXCL6 |
| BP | GO:0061418 | regulation of transcription from RNA polymerase II promoter in response to hypoxia | 0.078947748 | 0.044328733 | VEGFA |
| BP | GO:0071696 | ectodermal placode development | 0.078947748 | 0.044328733 | NRP1 |
| BP | GO:0072310 | glomerular epithelial cell development | 0.078947748 | 0.044328733 | JAG1 |
| BP | GO:0098814 | spontaneous synaptic transmission | 0.078947748 | 0.044328733 | APP |
| BP | GO:1900115 | extracellular regulation of signal transduction | 0.078947748 | 0.044328733 | LRPAP1 |
| BP | GO:1900116 | extracellular negative regulation of signal transduction | 0.078947748 | 0.044328733 | LRPAP1 |
| BP | GO:1900272 | negative regulation of long-term synaptic potentiation | 0.078947748 | 0.044328733 | APP |
| BP | GO:1902285 | semaphorin-plexin signaling pathway involved in neuron projection guidance | 0.078947748 | 0.044328733 | NRP1 |
| BP | GO:0030048 | actin filament-based movement | 0.078947748 | 0.044328733 | STC1/KCNJ8 |
| BP | GO:0006639 | acylglycerol metabolic process | 0.079447235 | 0.044609192 | LPL/APOH |
| BP | GO:0032612 | interleukin-1 production | 0.079447235 | 0.044609192 | LPL/APP |
| BP | GO:0032652 | regulation of interleukin-1 production | 0.079447235 | 0.044609192 | LPL/APP |
| BP | GO:0070304 | positive regulation of stress-activated protein kinase signaling cascade | 0.079447235 | 0.044609192 | VEGFA/APP |
| BP | GO:0019216 | regulation of lipid metabolic process | 0.080272426 | 0.045072532 | PDGFA/PTK2/VAV2 |
| BP | GO:0006638 | neutral lipid metabolic process | 0.080272426 | 0.045072532 | LPL/APOH |
| BP | GO:0030534 | adult behavior | 0.080990894 | 0.045475949 | CCND2/APP |
| BP | GO:0042476 | odontogenesis | 0.080990894 | 0.045475949 | MSX1/JAG2 |
| BP | GO:0045667 | regulation of osteoblast differentiation | 0.080990894 | 0.045475949 | JAG1/PTK2 |
| BP | GO:0002091 | negative regulation of receptor internalization | 0.080990894 | 0.045475949 | LRPAP1 |
| BP | GO:0010771 | negative regulation of cell morphogenesis involved in differentiation | 0.080990894 | 0.045475949 | POSTN |
| BP | GO:0035791 | platelet-derived growth factor receptor-beta signaling pathway | 0.080990894 | 0.045475949 | PDGFA |
| BP | GO:0043517 | positive regulation of DNA damage response, signal transduction by p53 class mediator | 0.080990894 | 0.045475949 | MSX1 |
| BP | GO:0048681 | negative regulation of axon regeneration | 0.080990894 | 0.045475949 | SPP1 |
| BP | GO:0048875 | chemical homeostasis within a tissue | 0.080990894 | 0.045475949 | VEGFA |
| BP | GO:0050930 | induction of positive chemotaxis | 0.080990894 | 0.045475949 | VEGFA |
| BP | GO:0060841 | venous blood vessel development | 0.080990894 | 0.045475949 | VEGFA |
| BP | GO:0070141 | response to UV-A | 0.080990894 | 0.045475949 | TIMP1 |
| BP | GO:0070942 | neutrophil mediated cytotoxicity | 0.080990894 | 0.045475949 | CXCL6 |
| BP | GO:0071481 | cellular response to X-ray | 0.080990894 | 0.045475949 | CCND2 |
| BP | GO:0097531 | mast cell migration | 0.080990894 | 0.045475949 | VEGFA |
| BP | GO:1900025 | negative regulation of substrate adhesion-dependent cell spreading | 0.080990894 | 0.045475949 | POSTN |
| BP | GO:1905049 | negative regulation of metallopeptidase activity | 0.080990894 | 0.045475949 | TIMP1 |
| BP | GO:0001101 | response to acid chemical | 0.083885651 | 0.047101339 | VEGFA/COL5A2 |
| BP | GO:0042157 | lipoprotein metabolic process | 0.083885651 | 0.047101339 | ITGAV/OLR1 |
| BP | GO:0072073 | kidney epithelium development | 0.083885651 | 0.047101339 | VEGFA/JAG1 |
| BP | GO:0006878 | cellular copper ion homeostasis | 0.083885651 | 0.047101339 | APP |
| BP | GO:0010763 | positive regulation of fibroblast migration | 0.083885651 | 0.047101339 | PTK2 |
| BP | GO:0010889 | regulation of sequestering of triglyceride | 0.083885651 | 0.047101339 | LPL |
| BP | GO:0032488 | Cdc42 protein signal transduction | 0.083885651 | 0.047101339 | NRP1 |
| BP | GO:0043252 | sodium-independent organic anion transport | 0.083885651 | 0.047101339 | SLCO2A1 |
| BP | GO:0045346 | regulation of MHC class II biosynthetic process | 0.083885651 | 0.047101339 | PF4 |
| BP | GO:0045836 | positive regulation of meiotic nuclear division | 0.083885651 | 0.047101339 | MSX1 |
| BP | GO:0048569 | post-embryonic animal organ development | 0.083885651 | 0.047101339 | VEGFA |
| BP | GO:0050746 | regulation of lipoprotein metabolic process | 0.083885651 | 0.047101339 | ITGAV |
| BP | GO:0090594 | inflammatory response to wounding | 0.083885651 | 0.047101339 | TIMP1 |
| BP | GO:0007266 | Rho protein signal transduction | 0.083885651 | 0.047101339 | COL3A1/NRP1 |
| BP | GO:0010977 | negative regulation of neuron projection development | 0.083885651 | 0.047101339 | SPP1/NRP1 |
| BP | GO:0030879 | mammary gland development | 0.083885651 | 0.047101339 | VEGFA/MSX1 |
| BP | GO:0060048 | cardiac muscle contraction | 0.083885651 | 0.047101339 | STC1/KCNJ8 |
| BP | GO:0070482 | response to oxygen levels | 0.084409978 | 0.047395746 | POSTN/STC1/VEGFA |
| BP | GO:0045598 | regulation of fat cell differentiation | 0.08582794 | 0.048191924 | LPL/JAG1 |
| BP | GO:0042742 | defense response to bacterium | 0.085959745 | 0.048265931 | PGLYRP1/PRG2/CXCL6 |
| BP | GO:0050768 | negative regulation of neurogenesis | 0.086638022 | 0.04864678 | SPP1/NRP1 |
| BP | GO:0003198 | epithelial to mesenchymal transition involved in endocardial cushion formation | 0.086786984 | 0.048730422 | MSX1 |
| BP | GO:0021783 | preganglionic parasympathetic fiber development | 0.086786984 | 0.048730422 | NRP1 |
| BP | GO:0030949 | positive regulation of vascular endothelial growth factor receptor signaling pathway | 0.086786984 | 0.048730422 | VTN |
| BP | GO:0042481 | regulation of odontogenesis | 0.086786984 | 0.048730422 | MSX1 |
| BP | GO:0045342 | MHC class II biosynthetic process | 0.086786984 | 0.048730422 | PF4 |
| BP | GO:0045651 | positive regulation of macrophage differentiation | 0.086786984 | 0.048730422 | PF4 |
| BP | GO:0061548 | ganglion development | 0.086786984 | 0.048730422 | NRP1 |
| BP | GO:0070571 | negative regulation of neuron projection regeneration | 0.086786984 | 0.048730422 | SPP1 |
| BP | GO:0098915 | membrane repolarization during ventricular cardiac muscle cell action potential | 0.086786984 | 0.048730422 | KCNJ8 |
| BP | GO:1905906 | regulation of amyloid fibril formation | 0.086786984 | 0.048730422 | APP |
| BP | GO:2001044 | regulation of integrin-mediated signaling pathway | 0.086786984 | 0.048730422 | TIMP1 |
| BP | GO:0050729 | positive regulation of inflammatory response | 0.086786984 | 0.048730422 | LPL/APP |
| BP | GO:0072006 | nephron development | 0.086786984 | 0.048730422 | VEGFA/JAG1 |
| BP | GO:0031644 | regulation of nervous system process | 0.088553237 | 0.049722163 | TNFRSF21/APP |
| BP | GO:0033135 | regulation of peptidyl-serine phosphorylation | 0.088553237 | 0.049722163 | VEGFA/APP |
| BP | GO:0046718 | viral entry into host cell | 0.088553237 | 0.049722163 | ITGAV/NRP1 |
| BP | GO:1901653 | cellular response to peptide | 0.088670681 | 0.049788107 | LPL/APP/PTK2 |
| BP | GO:0051961 | negative regulation of nervous system development | 0.089092 | 0.050024676 | SPP1/NRP1 |
| BP | GO:0070997 | neuron death | 0.089092 | 0.050024676 | TNFRSF21/APP/NRP1 |
| BP | GO:0003184 | pulmonary valve morphogenesis | 0.089092 | 0.050024676 | JAG1 |
| BP | GO:0016045 | detection of bacterium | 0.089092 | 0.050024676 | PGLYRP1 |
| BP | GO:0034349 | glial cell apoptotic process | 0.089092 | 0.050024676 | TNFRSF21 |
| BP | GO:0035729 | cellular response to hepatocyte growth factor stimulus | 0.089092 | 0.050024676 | NRP1 |
| BP | GO:0043117 | positive regulation of vascular permeability | 0.089092 | 0.050024676 | VEGFA |
| BP | GO:0048532 | anatomical structure arrangement | 0.089092 | 0.050024676 | NRP1 |
| BP | GO:0061298 | retina vasculature development in camera-type eye | 0.089092 | 0.050024676 | NRP1 |
| BP | GO:0090036 | regulation of protein kinase C signaling | 0.089092 | 0.050024676 | VEGFA |
| BP | GO:1901550 | regulation of endothelial cell development | 0.089092 | 0.050024676 | VEGFA |
| BP | GO:1903140 | regulation of establishment of endothelial barrier | 0.089092 | 0.050024676 | VEGFA |
| BP | GO:2000811 | negative regulation of anoikis | 0.089092 | 0.050024676 | PTK2 |
| BP | GO:0050767 | regulation of neurogenesis | 0.089535954 | 0.050273954 | VEGFA/SPP1/NRP1 |
| BP | GO:0010212 | response to ionizing radiation | 0.089734053 | 0.050385185 | THBD/CCND2 |
| BP | GO:0001701 | in utero embryonic development | 0.089734053 | 0.050385185 | VEGFA/MSX1/JAG2 |
| BP | GO:1903706 | regulation of hemopoiesis | 0.089734053 | 0.050385185 | PF4/JAG1/PGLYRP1 |
| BP | GO:0016525 | negative regulation of angiogenesis | 0.089734053 | 0.050385185 | PF4/APOH |
| BP | GO:0032368 | regulation of lipid transport | 0.089734053 | 0.050385185 | ITGAV/SPP1 |
| BP | GO:0045834 | positive regulation of lipid metabolic process | 0.089734053 | 0.050385185 | PTK2/VAV2 |
| BP | GO:0044409 | entry into host | 0.089734053 | 0.050385185 | ITGAV/NRP1 |
| BP | GO:0048754 | branching morphogenesis of an epithelial tube | 0.089734053 | 0.050385185 | VEGFA/NRP1 |
| BP | GO:0071456 | cellular response to hypoxia | 0.089734053 | 0.050385185 | STC1/VEGFA |
| BP | GO:2000181 | negative regulation of blood vessel morphogenesis | 0.089734053 | 0.050385185 | PF4/APOH |
| BP | GO:0003417 | growth plate cartilage development | 0.089734053 | 0.050385185 | STC1 |
| BP | GO:0010985 | negative regulation of lipoprotein particle clearance | 0.089734053 | 0.050385185 | LRPAP1 |
| BP | GO:0015732 | prostaglandin transport | 0.089734053 | 0.050385185 | SLCO2A1 |
| BP | GO:0030730 | sequestering of triglyceride | 0.089734053 | 0.050385185 | LPL |
| BP | GO:0030889 | negative regulation of B cell proliferation | 0.089734053 | 0.050385185 | TNFRSF21 |
| BP | GO:0032634 | interleukin-5 production | 0.089734053 | 0.050385185 | TNFRSF21 |
| BP | GO:0032674 | regulation of interleukin-5 production | 0.089734053 | 0.050385185 | TNFRSF21 |
| BP | GO:0043116 | negative regulation of vascular permeability | 0.089734053 | 0.050385185 | VEGFA |
| BP | GO:0045653 | negative regulation of megakaryocyte differentiation | 0.089734053 | 0.050385185 | PF4 |
| BP | GO:0048486 | parasympathetic nervous system development | 0.089734053 | 0.050385185 | NRP1 |
| BP | GO:0055070 | copper ion homeostasis | 0.089734053 | 0.050385185 | APP |
| BP | GO:0060749 | mammary gland alveolus development | 0.089734053 | 0.050385185 | VEGFA |
| BP | GO:0061377 | mammary gland lobule development | 0.089734053 | 0.050385185 | VEGFA |
| BP | GO:0061484 | hematopoietic stem cell homeostasis | 0.089734053 | 0.050385185 | FSTL1 |
| BP | GO:0071071 | regulation of phospholipid biosynthetic process | 0.089734053 | 0.050385185 | PDGFA |
| BP | GO:0090190 | positive regulation of branching involved in ureteric bud morphogenesis | 0.089734053 | 0.050385185 | VEGFA |
| BP | GO:0097709 | connective tissue replacement | 0.089734053 | 0.050385185 | TIMP1 |
| BP | GO:0030509 | BMP signaling pathway | 0.09043782 | 0.050780347 | FSTL1/MSX1 |
| BP | GO:1901343 | negative regulation of vasculature development | 0.09043782 | 0.050780347 | PF4/APOH |
| BP | GO:0002253 | activation of immune response | 0.091612049 | 0.05143967 | TNFRSF21/PTK2/VAV2 |
| BP | GO:0008360 | regulation of cell shape | 0.092111859 | 0.05172031 | VEGFA/PTK2 |
| BP | GO:0030856 | regulation of epithelial cell differentiation | 0.092111859 | 0.05172031 | VEGFA/JAG1 |
| BP | GO:0010759 | positive regulation of macrophage chemotaxis | 0.092111859 | 0.05172031 | PTK2 |
| BP | GO:0010885 | regulation of cholesterol storage | 0.092111859 | 0.05172031 | LPL |
| BP | GO:0031065 | positive regulation of histone deacetylation | 0.092111859 | 0.05172031 | VEGFA |
| BP | GO:0032616 | interleukin-13 production | 0.092111859 | 0.05172031 | TNFRSF21 |
| BP | GO:0032656 | regulation of interleukin-13 production | 0.092111859 | 0.05172031 | TNFRSF21 |
| BP | GO:0035728 | response to hepatocyte growth factor | 0.092111859 | 0.05172031 | NRP1 |
| BP | GO:0042474 | middle ear morphogenesis | 0.092111859 | 0.05172031 | MSX1 |
| BP | GO:0045780 | positive regulation of bone resorption | 0.092111859 | 0.05172031 | SPP1 |
| BP | GO:0045821 | positive regulation of glycolytic process | 0.092111859 | 0.05172031 | APP |
| BP | GO:1900221 | regulation of amyloid-beta clearance | 0.092111859 | 0.05172031 | LRPAP1 |
| BP | GO:2000251 | positive regulation of actin cytoskeleton reorganization | 0.092111859 | 0.05172031 | NRP1 |
| BP | GO:0035051 | cardiocyte differentiation | 0.092461288 | 0.051916513 | VEGFA/JAG1 |
| BP | GO:0051250 | negative regulation of lymphocyte activation | 0.093387844 | 0.052436769 | TNFRSF21/PGLYRP1 |
| BP | GO:0050727 | regulation of inflammatory response | 0.094357654 | 0.052981312 | LPL/APP/PGLYRP1 |
| BP | GO:0003215 | cardiac right ventricle morphogenesis | 0.094357654 | 0.052981312 | JAG1 |
| BP | GO:0010042 | response to manganese ion | 0.094357654 | 0.052981312 | APP |
| BP | GO:0031645 | negative regulation of nervous system process | 0.094357654 | 0.052981312 | TNFRSF21 |
| BP | GO:0032495 | response to muramyl dipeptide | 0.094357654 | 0.052981312 | JAG1 |
| BP | GO:0032793 | positive regulation of CREB transcription factor activity | 0.094357654 | 0.052981312 | VEGFA |
| BP | GO:0046629 | gamma-delta T cell activation | 0.094357654 | 0.052981312 | JAG2 |
| BP | GO:0048670 | regulation of collateral sprouting | 0.094357654 | 0.052981312 | SPP1 |
| BP | GO:0051004 | regulation of lipoprotein lipase activity | 0.094357654 | 0.052981312 | APOH |
| BP | GO:0061318 | renal filtration cell differentiation | 0.094357654 | 0.052981312 | JAG1 |
| BP | GO:0072112 | glomerular visceral epithelial cell differentiation | 0.094357654 | 0.052981312 | JAG1 |
| BP | GO:0097150 | neuronal stem cell population maintenance | 0.094357654 | 0.052981312 | JAG1 |
| BP | GO:0098543 | detection of other organism | 0.094357654 | 0.052981312 | PGLYRP1 |
| BP | GO:2000678 | negative regulation of transcription regulatory region DNA binding | 0.094357654 | 0.052981312 | MSX1 |
| BP | GO:0003205 | cardiac chamber development | 0.095229691 | 0.053470956 | JAG1/NRP1 |
| BP | GO:0036294 | cellular response to decreased oxygen levels | 0.095229691 | 0.053470956 | STC1/VEGFA |
| BP | GO:0051100 | negative regulation of binding | 0.09615015 | 0.053987789 | LRPAP1/MSX1 |
| BP | GO:0046486 | glycerolipid metabolic process | 0.096634125 | 0.054259538 | LPL/PDGFA/APOH |
| BP | GO:0003177 | pulmonary valve development | 0.096634125 | 0.054259538 | JAG1 |
| BP | GO:0007350 | blastoderm segmentation | 0.096634125 | 0.054259538 | NRP1 |
| BP | GO:0007617 | mating behavior | 0.096634125 | 0.054259538 | APP |
| BP | GO:0010878 | cholesterol storage | 0.096634125 | 0.054259538 | LPL |
| BP | GO:0023019 | signal transduction involved in regulation of gene expression | 0.096634125 | 0.054259538 | MSX1 |
| BP | GO:0038083 | peptidyl-tyrosine autophosphorylation | 0.096634125 | 0.054259538 | VEGFA |
| BP | GO:0045056 | transcytosis | 0.096634125 | 0.054259538 | LRPAP1 |
| BP | GO:0048485 | sympathetic nervous system development | 0.096634125 | 0.054259538 | NRP1 |
| BP | GO:0072311 | glomerular epithelial cell differentiation | 0.096634125 | 0.054259538 | JAG1 |
| BP | GO:0090189 | regulation of branching involved in ureteric bud morphogenesis | 0.096634125 | 0.054259538 | VEGFA |
| BP | GO:2000047 | regulation of cell-cell adhesion mediated by cadherin | 0.096634125 | 0.054259538 | VEGFA |
| BP | GO:0032635 | interleukin-6 production | 0.096899742 | 0.05440868 | LPL/APP |
| BP | GO:0032675 | regulation of interleukin-6 production | 0.096899742 | 0.05440868 | LPL/APP |
| BP | GO:0071772 | response to BMP | 0.096899742 | 0.05440868 | FSTL1/MSX1 |
| BP | GO:0071773 | cellular response to BMP stimulus | 0.096899742 | 0.05440868 | FSTL1/MSX1 |
| BP | GO:0032970 | regulation of actin filament-based process | 0.097505019 | 0.05474854 | STC1/NRP1/PDGFA |
| BP | GO:0030307 | positive regulation of cell growth | 0.097505019 | 0.05474854 | VEGFA/NRP1 |
| BP | GO:0043112 | receptor metabolic process | 0.097505019 | 0.05474854 | LRPAP1/VEGFA |
| BP | GO:0002053 | positive regulation of mesenchymal cell proliferation | 0.098189739 | 0.055133007 | PDGFA |
| BP | GO:0007342 | fusion of sperm to egg plasma membrane involved in single fertilization | 0.098189739 | 0.055133007 | SERPINA5 |
| BP | GO:0021854 | hypothalamus development | 0.098189739 | 0.055133007 | NRP1 |
| BP | GO:0032693 | negative regulation of interleukin-10 production | 0.098189739 | 0.055133007 | TNFRSF21 |
| BP | GO:0045061 | thymic T cell selection | 0.098189739 | 0.055133007 | JAG2 |
| BP | GO:0048169 | regulation of long-term neuronal synaptic plasticity | 0.098189739 | 0.055133007 | APP |
| BP | GO:0061042 | vascular wound healing | 0.098189739 | 0.055133007 | VEGFA |
| BP | GO:0071404 | cellular response to low-density lipoprotein particle stimulus | 0.098189739 | 0.055133007 | LPL |
| BP | GO:0090280 | positive regulation of calcium ion import | 0.098189739 | 0.055133007 | STC1 |
| BP | GO:1900120 | regulation of receptor binding | 0.098189739 | 0.055133007 | NRP1 |
| BP | GO:1901522 | positive regulation of transcription from RNA polymerase II promoter involved in cellular response to chemical stimulus | 0.098189739 | 0.055133007 | VEGFA |
| BP | GO:1903589 | positive regulation of blood vessel endothelial cell proliferation involved in sprouting angiogenesis | 0.098189739 | 0.055133007 | VEGFA |
| BP | GO:0048469 | cell maturation | 0.10024766 | 0.056288518 | VEGFA/APP |
| BP | GO:0002052 | positive regulation of neuroblast proliferation | 0.10024766 | 0.056288518 | VEGFA |
| BP | GO:0006929 | substrate-dependent cell migration | 0.10024766 | 0.056288518 | NRP1 |
| BP | GO:0010888 | negative regulation of lipid storage | 0.10024766 | 0.056288518 | ITGAV |
| BP | GO:0021884 | forebrain neuron development | 0.10024766 | 0.056288518 | NRP1 |
| BP | GO:0035162 | embryonic hemopoiesis | 0.10024766 | 0.056288518 | VEGFA |
| BP | GO:0035988 | chondrocyte proliferation | 0.10024766 | 0.056288518 | STC1 |
| BP | GO:0035994 | response to muscle stretch | 0.10024766 | 0.056288518 | PTK2 |
| BP | GO:0036303 | lymph vessel morphogenesis | 0.10024766 | 0.056288518 | VEGFA |
| BP | GO:0051446 | positive regulation of meiotic cell cycle | 0.10024766 | 0.056288518 | MSX1 |
| BP | GO:0060384 | innervation | 0.10024766 | 0.056288518 | NRP1 |
| BP | GO:0060396 | growth hormone receptor signaling pathway | 0.10024766 | 0.056288518 | PTK2 |
| BP | GO:0071636 | positive regulation of transforming growth factor beta production | 0.10024766 | 0.056288518 | LUM |
| BP | GO:1900273 | positive regulation of long-term synaptic potentiation | 0.10024766 | 0.056288518 | APP |
| BP | GO:2000737 | negative regulation of stem cell differentiation | 0.10024766 | 0.056288518 | JAG1 |
| BP | GO:0048639 | positive regulation of developmental growth | 0.101462159 | 0.056970453 | VEGFA/NRP1 |
| BP | GO:0052126 | movement in host environment | 0.101462159 | 0.056970453 | ITGAV/NRP1 |
| BP | GO:0043434 | response to peptide hormone | 0.101462159 | 0.056970453 | LPL/PTK2/TIMP1 |
| BP | GO:0001779 | natural killer cell differentiation | 0.101462159 | 0.056970453 | PGLYRP1 |
| BP | GO:0001783 | B cell apoptotic process | 0.101462159 | 0.056970453 | TNFRSF21 |
| BP | GO:0003148 | outflow tract septum morphogenesis | 0.101462159 | 0.056970453 | NRP1 |
| BP | GO:0010288 | response to lead ion | 0.101462159 | 0.056970453 | APP |
| BP | GO:0031639 | plasminogen activation | 0.101462159 | 0.056970453 | APOH |
| BP | GO:0033032 | regulation of myeloid cell apoptotic process | 0.101462159 | 0.056970453 | APOH |
| BP | GO:0036003 | positive regulation of transcription from RNA polymerase II promoter in response to stress | 0.101462159 | 0.056970453 | VEGFA |
| BP | GO:0036010 | protein localization to endosome | 0.101462159 | 0.056970453 | NRP1 |
| BP | GO:0038095 | Fc-epsilon receptor signaling pathway | 0.101462159 | 0.056970453 | VAV2 |
| BP | GO:0044062 | regulation of excretion | 0.101462159 | 0.056970453 | STC1 |
| BP | GO:0045649 | regulation of macrophage differentiation | 0.101462159 | 0.056970453 | PF4 |
| BP | GO:0051043 | regulation of membrane protein ectodomain proteolysis | 0.101462159 | 0.056970453 | TIMP1 |
| BP | GO:0060561 | apoptotic process involved in morphogenesis | 0.101462159 | 0.056970453 | JAG2 |
| BP | GO:0071378 | cellular response to growth hormone stimulus | 0.101462159 | 0.056970453 | PTK2 |
| BP | GO:0072010 | glomerular epithelium development | 0.101462159 | 0.056970453 | JAG1 |
| BP | GO:2000209 | regulation of anoikis | 0.101462159 | 0.056970453 | PTK2 |
| BP | GO:2000310 | regulation of NMDA receptor activity | 0.101462159 | 0.056970453 | APP |
| BP | GO:0030324 | lung development | 0.101514746 | 0.056999981 | VEGFA/PDGFA |
| BP | GO:0051302 | regulation of cell division | 0.101514746 | 0.056999981 | VEGFA/PDGFA |
| BP | GO:0071453 | cellular response to oxygen levels | 0.101514746 | 0.056999981 | STC1/VEGFA |
| BP | GO:0006941 | striated muscle contraction | 0.103184941 | 0.057937786 | STC1/KCNJ8 |
| BP | GO:0007626 | locomotory behavior | 0.103184941 | 0.057937786 | CCND2/APP |
| BP | GO:0032469 | endoplasmic reticulum calcium ion homeostasis | 0.103184941 | 0.057937786 | APP |
| BP | GO:0042832 | defense response to protozoan | 0.103184941 | 0.057937786 | PF4 |
| BP | GO:0048143 | astrocyte activation | 0.103184941 | 0.057937786 | APP |
| BP | GO:0050927 | positive regulation of positive chemotaxis | 0.103184941 | 0.057937786 | VEGFA |
| BP | GO:0062009 | secondary palate development | 0.103184941 | 0.057937786 | JAG2 |
| BP | GO:0086013 | membrane repolarization during cardiac muscle cell action potential | 0.103184941 | 0.057937786 | KCNJ8 |
| BP | GO:0090312 | positive regulation of protein deacetylation | 0.103184941 | 0.057937786 | VEGFA |
| BP | GO:0098581 | detection of external biotic stimulus | 0.103184941 | 0.057937786 | PGLYRP1 |
| BP | GO:1901798 | positive regulation of signal transduction by p53 class mediator | 0.103184941 | 0.057937786 | MSX1 |
| BP | GO:1905048 | regulation of metallopeptidase activity | 0.103184941 | 0.057937786 | TIMP1 |
| BP | GO:1905564 | positive regulation of vascular endothelial cell proliferation | 0.103184941 | 0.057937786 | FGFR1 |
| BP | GO:0030323 | respiratory tube development | 0.103267406 | 0.057984089 | VEGFA/PDGFA |
| BP | GO:0045766 | positive regulation of angiogenesis | 0.103267406 | 0.057984089 | VEGFA/NRP1 |
| BP | GO:1904018 | positive regulation of vasculature development | 0.103267406 | 0.057984089 | VEGFA/NRP1 |
| BP | GO:1903131 | mononuclear cell differentiation | 0.104810193 | 0.058850356 | VEGFA/PGLYRP1/JAG2 |
| BP | GO:0048568 | embryonic organ development | 0.105116359 | 0.059022267 | VEGFA/PDGFA/MSX1 |
| BP | GO:0001562 | response to protozoan | 0.105116359 | 0.059022267 | PF4 |
| BP | GO:0002092 | positive regulation of receptor internalization | 0.105116359 | 0.059022267 | VEGFA |
| BP | GO:0003416 | endochondral bone growth | 0.105116359 | 0.059022267 | STC1 |
| BP | GO:0006027 | glycosaminoglycan catabolic process | 0.105116359 | 0.059022267 | PGLYRP1 |
| BP | GO:0006706 | steroid catabolic process | 0.105116359 | 0.059022267 | SPP1 |
| BP | GO:0045026 | plasma membrane fusion | 0.105116359 | 0.059022267 | SERPINA5 |
| BP | GO:0048843 | negative regulation of axon extension involved in axon guidance | 0.105116359 | 0.059022267 | NRP1 |
| BP | GO:0050926 | regulation of positive chemotaxis | 0.105116359 | 0.059022267 | VEGFA |
| BP | GO:1905523 | positive regulation of macrophage migration | 0.105116359 | 0.059022267 | PTK2 |
| BP | GO:0002040 | sprouting angiogenesis | 0.105587283 | 0.059286688 | VEGFA/NRP1 |
| BP | GO:0031345 | negative regulation of cell projection organization | 0.106311964 | 0.059693592 | SPP1/NRP1 |
| BP | GO:0071478 | cellular response to radiation | 0.106311964 | 0.059693592 | CCND2/TIMP1 |
| CC | GO:0062023 | collagen-containing extracellular matrix | 1.07E-09 | 6.83E-10 | VCAN/POSTN/PF4/COL5A2/SERPINA5/COL3A1/S100A4/VTN/TIMP1/APOH/PRG2/LUM |
| CC | GO:0005788 | endoplasmic reticulum lumen | 2.86E-07 | 1.82E-07 | VCAN/FSTL1/LRPAP1/COL5A2/APP/COL3A1/SPP1/PDGFA/TIMP1 |
| CC | GO:0031091 | platelet alpha granule | 7.19E-07 | 4.57E-07 | VEGFA/PF4/SERPINA5/APP/PDGFA/TIMP1 |
| CC | GO:0031093 | platelet alpha granule lumen | 5.08E-06 | 3.23E-06 | VEGFA/PF4/APP/PDGFA/TIMP1 |
| CC | GO:0005583 | fibrillar collagen trimer | 2.63E-05 | 1.67E-05 | COL5A2/COL3A1/LUM |
| CC | GO:0098643 | banded collagen fibril | 2.63E-05 | 1.67E-05 | COL5A2/COL3A1/LUM |
| CC | GO:0005796 | Golgi lumen | 2.63E-05 | 1.67E-05 | VCAN/LRPAP1/APP/PDGFA/LUM |
| CC | GO:0034774 | secretory granule lumen | 2.72E-05 | 1.73E-05 | VEGFA/PF4/APP/PDGFA/PGLYRP1/TIMP1/APOH |
| CC | GO:0060205 | cytoplasmic vesicle lumen | 2.72E-05 | 1.73E-05 | VEGFA/PF4/APP/PDGFA/PGLYRP1/TIMP1/APOH |
| CC | GO:0031983 | vesicle lumen | 2.72E-05 | 1.73E-05 | VEGFA/PF4/APP/PDGFA/PGLYRP1/TIMP1/APOH |
| CC | GO:0098644 | complex of collagen trimers | 9.76E-05 | 6.21E-05 | COL5A2/COL3A1/LUM |
| CC | GO:0042627 | chylomicron | 0.00304674 | 0.00193762 | LPL/APOH |
| CC | GO:0005581 | collagen trimer | 0.005889285 | 0.003745379 | COL5A2/COL3A1/LUM |
| CC | GO:0034361 | very-low-density lipoprotein particle | 0.005889285 | 0.003745379 | LPL/APOH |
| CC | GO:0034385 | triglyceride-rich plasma lipoprotein particle | 0.005889285 | 0.003745379 | LPL/APOH |
| CC | GO:0031904 | endosome lumen | 0.015974543 | 0.010159249 | LRPAP1/APP |
| CC | GO:0034358 | plasma lipoprotein particle | 0.015974543 | 0.010159249 | LPL/APOH |
| CC | GO:1990777 | lipoprotein particle | 0.015974543 | 0.010159249 | LPL/APOH |
| CC | GO:0032994 | protein-lipid complex | 0.01773859 | 0.011281121 | LPL/APOH |
| CC | GO:0042581 | specific granule | 0.022685607 | 0.01442725 | ITGAV/OLR1/PGLYRP1 |
| CC | GO:0031594 | neuromuscular junction | 0.050468481 | 0.032096183 | POSTN/APP |
| CC | GO:0098858 | actin-based cell projection | 0.050602763 | 0.032181582 | ITGAV/APP/PDGFA |
| CC | GO:0005791 | rough endoplasmic reticulum | 0.062497889 | 0.039746464 | LRPAP1/APP |
| CC | GO:0005902 | microvillus | 0.070168944 | 0.044624986 | ITGAV/PDGFA |
| CC | GO:0035579 | specific granule membrane | 0.070168944 | 0.044624986 | ITGAV/OLR1 |
| CC | GO:0043202 | lysosomal lumen | 0.076186442 | 0.048451904 | VCAN/LUM |
| CC | GO:0030175 | filopodium | 0.088338534 | 0.056180208 | ITGAV/APP |
| CC | GO:0005641 | nuclear envelope lumen | 0.093253335 | 0.059305849 | APP |
| CC | GO:0030667 | secretory granule membrane | 0.093253335 | 0.059305849 | ITGAV/SERPINA5/OLR1 |
| CC | GO:0002116 | semaphorin receptor complex | 0.093253335 | 0.059305849 | NRP1 |
| CC | GO:0031094 | platelet dense tubular network | 0.093253335 | 0.059305849 | SERPINA5 |
| CC | GO:0018995 | host cellular component | 0.095480079 | 0.06072198 | PF4 |
| CC | GO:0043657 | host cell | 0.095480079 | 0.06072198 | PF4 |
| CC | GO:0035253 | ciliary rootlet | 0.100304884 | 0.063790387 | APP |
| CC | GO:0031089 | platelet dense granule lumen | 0.104840703 | 0.066675009 | APOH |
| CC | GO:0045335 | phagocytic vesicle | 0.105163297 | 0.066880167 | ITGAV/PGLYRP1 |
| CC | GO:0031527 | filopodium membrane | 0.119016319 | 0.075690203 | ITGAV |
| CC | GO:0090533 | cation-transporting ATPase complex | 0.119016319 | 0.075690203 | KCNJ8 |
| CC | GO:0097449 | astrocyte projection | 0.119016319 | 0.075690203 | APP |
| CC | GO:0070820 | tertiary granule | 0.119016319 | 0.075690203 | OLR1/PGLYRP1 |
| CC | GO:0016327 | apicolateral plasma membrane | 0.119016319 | 0.075690203 | THBD |
| CC | GO:0042827 | platelet dense granule | 0.119016319 | 0.075690203 | APOH |
| CC | GO:0005912 | adherens junction | 0.119016319 | 0.075690203 | VEGFA/JAG1 |
| CC | GO:0031258 | lamellipodium membrane | 0.119016319 | 0.075690203 | ITGAV |
| CC | GO:0005775 | vacuolar lumen | 0.119016319 | 0.075690203 | VCAN/LUM |
| CC | GO:0005925 | focal adhesion | 0.119016319 | 0.075690203 | ITGAV/NRP1/PTK2 |
| CC | GO:0071682 | endocytic vesicle lumen | 0.119016319 | 0.075690203 | PGLYRP1 |
| CC | GO:0009897 | external side of plasma membrane | 0.119016319 | 0.075690203 | THBD/ITGAV/SERPINA5 |
| CC | GO:0030426 | growth cone | 0.119016319 | 0.075690203 | APP/NRP1 |
| CC | GO:0030055 | cell-substrate junction | 0.119016319 | 0.075690203 | ITGAV/NRP1/PTK2 |
| CC | GO:0098533 | ATPase dependent transmembrane transport complex | 0.119016319 | 0.075690203 | KCNJ8 |
| CC | GO:0030427 | site of polarized growth | 0.119016319 | 0.075690203 | APP/NRP1 |
| CC | GO:0043197 | dendritic spine | 0.119016319 | 0.075690203 | APP/PTK2 |
| CC | GO:0044309 | neuron spine | 0.119016319 | 0.075690203 | APP/PTK2 |
| CC | GO:0045177 | apical part of cell | 0.119016319 | 0.075690203 | STC1/APP/JAG1 |
| CC | GO:0031528 | microvillus membrane | 0.12272357 | 0.078047884 | ITGAV |
| CC | GO:0034364 | high-density lipoprotein particle | 0.12272357 | 0.078047884 | APOH |
| MF | GO:0005539 | glycosaminoglycan binding | 1.93E-19 | 1.14E-19 | VCAN/POSTN/FSTL1/LRPAP1/LPL/VEGFA/PF4/FGFR1/SERPINA5/APP/NRP1/PGLYRP1/VTN/APOH/PRG2/CXCL6 |
| MF | GO:0008201 | heparin binding | 3.40E-18 | 2.01E-18 | POSTN/FSTL1/LRPAP1/LPL/VEGFA/PF4/FGFR1/SERPINA5/APP/NRP1/VTN/APOH/PRG2/CXCL6 |
| MF | GO:1901681 | sulfur compound binding | 1.75E-15 | 1.04E-15 | POSTN/FSTL1/LRPAP1/LPL/VEGFA/PF4/FGFR1/SERPINA5/APP/NRP1/VTN/APOH/PRG2/CXCL6 |
| MF | GO:0030546 | signaling receptor activator activity | 6.09E-08 | 3.61E-08 | LRPAP1/STC1/VEGFA/PF4/APP/JAG1/SPP1/PDGFA/TIMP1/JAG2/CXCL6 |
| MF | GO:0048018 | receptor ligand activity | 6.56E-07 | 3.88E-07 | LRPAP1/STC1/VEGFA/PF4/JAG1/SPP1/PDGFA/TIMP1/JAG2/CXCL6 |
| MF | GO:0005201 | extracellular matrix structural constituent | 9.46E-07 | 5.60E-07 | VCAN/POSTN/COL5A2/COL3A1/VTN/PRG2/LUM |
| MF | GO:0019838 | growth factor binding | 0.000163748 | 9.69E-05 | FGFR1/ITGAV/COL3A1/NRP1/PDGFA |
| MF | GO:0005178 | integrin binding | 0.000163748 | 9.69E-05 | ITGAV/COL3A1/SPP1/PTK2/VTN |
| MF | GO:0030021 | extracellular matrix structural constituent conferring compression resistance | 0.000176434 | 0.000104391 | VCAN/PRG2/LUM |
| MF | GO:0008083 | growth factor activity | 0.000231971 | 0.000137252 | VEGFA/JAG1/PDGFA/TIMP1/JAG2 |
| MF | GO:0005125 | cytokine activity | 0.001246215 | 0.000737355 | VEGFA/PF4/SPP1/TIMP1/CXCL6 |
| MF | GO:0002020 | protease binding | 0.001746335 | 0.001033263 | ITGAV/SERPINA5/COL3A1/TIMP1 |
| MF | GO:0070851 | growth factor receptor binding | 0.001905963 | 0.001127711 | VEGFA/APP/PDGFA/VAV2 |
| MF | GO:0050840 | extracellular matrix binding | 0.001950241 | 0.00115391 | VEGFA/ITGAV/SPP1 |
| MF | GO:0048407 | platelet-derived growth factor binding | 0.00207209 | 0.001226004 | COL3A1/PDGFA |
| MF | GO:0005161 | platelet-derived growth factor receptor binding | 0.003690318 | 0.00218347 | VEGFA/PDGFA |
| MF | GO:0045236 | CXCR chemokine receptor binding | 0.005042322 | 0.002983417 | PF4/CXCL6 |
| MF | GO:0017134 | fibroblast growth factor binding | 0.007826363 | 0.004630665 | FGFR1/ITGAV |
| MF | GO:0005112 | Notch binding | 0.008770203 | 0.005189112 | JAG1/JAG2 |
| MF | GO:0001968 | fibronectin binding | 0.010459218 | 0.006188461 | VEGFA/ITGAV |
| MF | GO:0004714 | transmembrane receptor protein tyrosine kinase activity | 0.013094939 | 0.007747951 | FGFR1/NRP1/PTK2 |
| MF | GO:0030246 | carbohydrate binding | 0.013094939 | 0.007747951 | VCAN/OLR1/VTN/PRG2 |
| MF | GO:0004713 | protein tyrosine kinase activity | 0.015771663 | 0.009331703 | FGFR1/NRP1/PTK2 |
| MF | GO:0019199 | transmembrane receptor protein kinase activity | 0.017425605 | 0.010310299 | FGFR1/NRP1/PTK2 |
| MF | GO:0030020 | extracellular matrix structural constituent conferring tensile strength | 0.017863423 | 0.010569346 | COL5A2/COL3A1 |
| MF | GO:0015026 | coreceptor activity | 0.023425587 | 0.01386034 | ITGAV/NRP1 |
| MF | GO:0008009 | chemokine activity | 0.023489015 | 0.013897869 | PF4/CXCL6 |
| MF | GO:0004866 | endopeptidase inhibitor activity | 0.028473684 | 0.016847175 | SERPINA5/APP/TIMP1 |
| MF | GO:0030414 | peptidase inhibitor activity | 0.030557781 | 0.018080283 | SERPINA5/APP/TIMP1 |
| MF | GO:0061135 | endopeptidase regulator activity | 0.032694259 | 0.019344384 | SERPINA5/APP/TIMP1 |
| MF | GO:0005518 | collagen binding | 0.03982274 | 0.02356213 | PDGFA/LUM |
| MF | GO:0042379 | chemokine receptor binding | 0.041877371 | 0.024777805 | PF4/CXCL6 |
| MF | GO:0038024 | cargo receptor activity | 0.04592946 | 0.027175326 | OLR1/VTN |
| MF | GO:0061134 | peptidase regulator activity | 0.04592946 | 0.027175326 | SERPINA5/APP/TIMP1 |
| MF | GO:0004867 | serine-type endopeptidase inhibitor activity | 0.067320837 | 0.039832074 | SERPINA5/APP |
| MF | GO:0005126 | cytokine receptor binding | 0.067320837 | 0.039832074 | VEGFA/PF4/CXCL6 |
| MF | GO:0004465 | lipoprotein lipase activity | 0.076233478 | 0.045105463 | LPL |
| MF | GO:0050786 | RAGE receptor binding | 0.076233478 | 0.045105463 | S100A4 |
| MF | GO:0052740 | 1-acyl-2-lysophosphatidylserine acylhydrolase activity | 0.076233478 | 0.045105463 | LPL |
| MF | GO:0052739 | phosphatidylserine 1-acylhydrolase activity | 0.081682702 | 0.048329634 | LPL |
| MF | GO:0017154 | semaphorin receptor activity | 0.084784513 | 0.0501649 | NRP1 |
| MF | GO:1902282 | voltage-gated potassium channel activity involved in ventricular cardiac muscle cell action potential repolarization | 0.084784513 | 0.0501649 | KCNJ8 |
| MF | GO:0031994 | insulin-like growth factor I binding | 0.087591617 | 0.051825794 | ITGAV |
| MF | GO:0061783 | peptidoglycan muralytic activity | 0.087591617 | 0.051825794 | PGLYRP1 |
| MF | GO:0005172 | vascular endothelial growth factor receptor binding | 0.090960134 | 0.053818861 | VEGFA |
| MF | GO:0001846 | opsonin binding | 0.090960134 | 0.053818861 | ITGAV |
| MF | GO:0005041 | low-density lipoprotein particle receptor activity | 0.090960134 | 0.053818861 | OLR1 |
| MF | GO:0086008 | voltage-gated potassium channel activity involved in cardiac muscle cell action potential repolarization | 0.090960134 | 0.053818861 | KCNJ8 |
| MF | GO:0019955 | cytokine binding | 0.090960134 | 0.053818861 | ITGAV/NRP1 |
| MF | GO:0008191 | metalloendopeptidase inhibitor activity | 0.090960134 | 0.053818861 | TIMP1 |
| MF | GO:0008970 | phospholipase A1 activity | 0.090960134 | 0.053818861 | LPL |
| MF | GO:0015347 | sodium-independent organic anion transmembrane transporter activity | 0.090960134 | 0.053818861 | SLCO2A1 |
| MF | GO:0022843 | voltage-gated cation channel activity | 0.091286897 | 0.054012199 | ITGAV/KCNJ8 |
| MF | GO:0034185 | apolipoprotein binding | 0.091286897 | 0.054012199 | LPL |
| MF | GO:0043395 | heparan sulfate proteoglycan binding | 0.091286897 | 0.054012199 | LPL |
| MF | GO:0030228 | lipoprotein particle receptor activity | 0.091570292 | 0.054179877 | OLR1 |
| MF | GO:0042834 | peptidoglycan binding | 0.091570292 | 0.054179877 | PGLYRP1 |
| MF | GO:0060229 | lipase activator activity | 0.091570292 | 0.054179877 | APOH |
| MF | GO:0001972 | retinoic acid binding | 0.099830714 | 0.059067365 | SERPINA5 |
| MF | GO:0005540 | hyaluronic acid binding | 0.107779012 | 0.063770176 | VCAN |
| MF | GO:0050750 | low-density lipoprotein particle receptor binding | 0.110725903 | 0.065513778 | LRPAP1 |
| MF | GO:0043495 | protein-membrane adaptor activity | 0.111766205 | 0.066129299 | LPL |
| MF | GO:0050431 | transforming growth factor beta binding | 0.111766205 | 0.066129299 | ITGAV |
| MF | GO:0005242 | inward rectifier potassium channel activity | 0.112734063 | 0.066701957 | KCNJ8 |
| MF | GO:0030247 | polysaccharide binding | 0.112734063 | 0.066701957 | VTN |
| MF | GO:0004806 | triglyceride lipase activity | 0.113635981 | 0.0672356 | LPL |
| MF | GO:0038187 | pattern recognition receptor activity | 0.113635981 | 0.0672356 | PGLYRP1 |
| KEGG | hsa04510 | Focal adhesion | 4.30E-05 | 3.29E-05 | VEGFA/CCND2/ITGAV/SPP1/PDGFA/PTK2/VAV2/VTN |
| KEGG | hsa05205 | Proteoglycans in cancer | 0.000352384 | 0.000269768 | VEGFA/FGFR1/ITGAV/PTK2/VAV2/VTN/LUM |
| KEGG | hsa04151 | PI3K-Akt signaling pathway | 0.000969549 | 0.000742238 | VEGFA/FGFR1/CCND2/ITGAV/SPP1/PDGFA/PTK2/VTN |
| KEGG | hsa05418 | Fluid shear stress and atherosclerosis | 0.003056058 | 0.002339566 | VEGFA/THBD/ITGAV/PDGFA/PTK2 |
| KEGG | hsa05165 | Human papillomavirus infection | 0.003056058 | 0.002339566 | VEGFA/CCND2/ITGAV/JAG1/SPP1/PTK2/VTN |
| KEGG | hsa04979 | Cholesterol metabolism | 0.012921504 | 0.00989206 | LRPAP1/LPL/APOH |
| KEGG | hsa04810 | Regulation of actin cytoskeleton | 0.015207825 | 0.011642354 | FGFR1/ITGAV/PDGFA/PTK2/VAV2 |
| KEGG | hsa04610 | Complement and coagulation cascades | 0.043901145 | 0.033608532 | THBD/SERPINA5/VTN |
| KEGG | hsa04512 | ECM-receptor interaction | 0.043901145 | 0.033608532 | ITGAV/SPP1/VTN |
| KEGG | hsa04062 | Chemokine signaling pathway | 0.047100699 | 0.036057952 | PF4/PTK2/VAV2/CXCL6 |
| KEGG | hsa01522 | Endocrine resistance | 0.047100699 | 0.036057952 | JAG1/PTK2/JAG2 |
| KEGG | hsa04933 | AGE-RAGE signaling pathway in diabetic complications | 0.047100699 | 0.036057952 | VEGFA/THBD/COL3A1 |
| KEGG | hsa04015 | Rap1 signaling pathway | 0.052703997 | 0.040347557 | VEGFA/FGFR1/PDGFA/VAV2 |
| KEGG | hsa05224 | Breast cancer | 0.115338676 | 0.088297551 | FGFR1/JAG1/JAG2 |
| KEGG | hsa04330 | Notch signaling pathway | 0.121580932 | 0.093076311 | JAG1/JAG2 |
| KEGG | hsa04370 | VEGF signaling pathway | 0.121580932 | 0.093076311 | VEGFA/PTK2 |
| KEGG | hsa05218 | Melanoma | 0.165987156 | 0.127071507 | FGFR1/PDGFA |
| KEGG | hsa03320 | PPAR signaling pathway | 0.16740224 | 0.128154825 | LPL/OLR1 |
| KEGG | hsa05202 | Transcriptional misregulation in cancer | 0.16740224 | 0.128154825 | CCND2/PDGFA/PTK2 |
| KEGG | hsa01521 | EGFR tyrosine kinase inhibitor resistance | 0.16740224 | 0.128154825 | VEGFA/PDGFA |
| KEGG | hsa05417 | Lipid and atherosclerosis | 0.174459454 | 0.133557477 | OLR1/PTK2/VAV2 |
| KEGG | hsa04658 | Th1 and Th2 cell differentiation | 0.174459454 | 0.133557477 | JAG1/JAG2 |
| KEGG | hsa05222 | Small cell lung cancer | 0.174459454 | 0.133557477 | ITGAV/PTK2 |
| KEGG | hsa05323 | Rheumatoid arthritis | 0.174459454 | 0.133557477 | VEGFA/CXCL6 |
| KEGG | hsa05166 | Human T-cell leukemia virus 1 infection | 0.174459454 | 0.133557477 | CCND2/NRP1/MSX1 |
| KEGG | hsa05163 | Human cytomegalovirus infection | 0.174459454 | 0.133557477 | VEGFA/ITGAV/PTK2 |
| KEGG | hsa05215 | Prostate cancer | 0.174459454 | 0.133557477 | FGFR1/PDGFA |
