# Supplementary figures and images for: Gastric Cancer Subtypes in Tumour and Nontumour Tissues by Immunologic and Hallmark Gene Sets
Source: J Oncol. 2022 Aug 27;2022:7887711. doi: 10.1155/2022/7887711 (PMC9440817; doi:10.1155/2022/7887711)

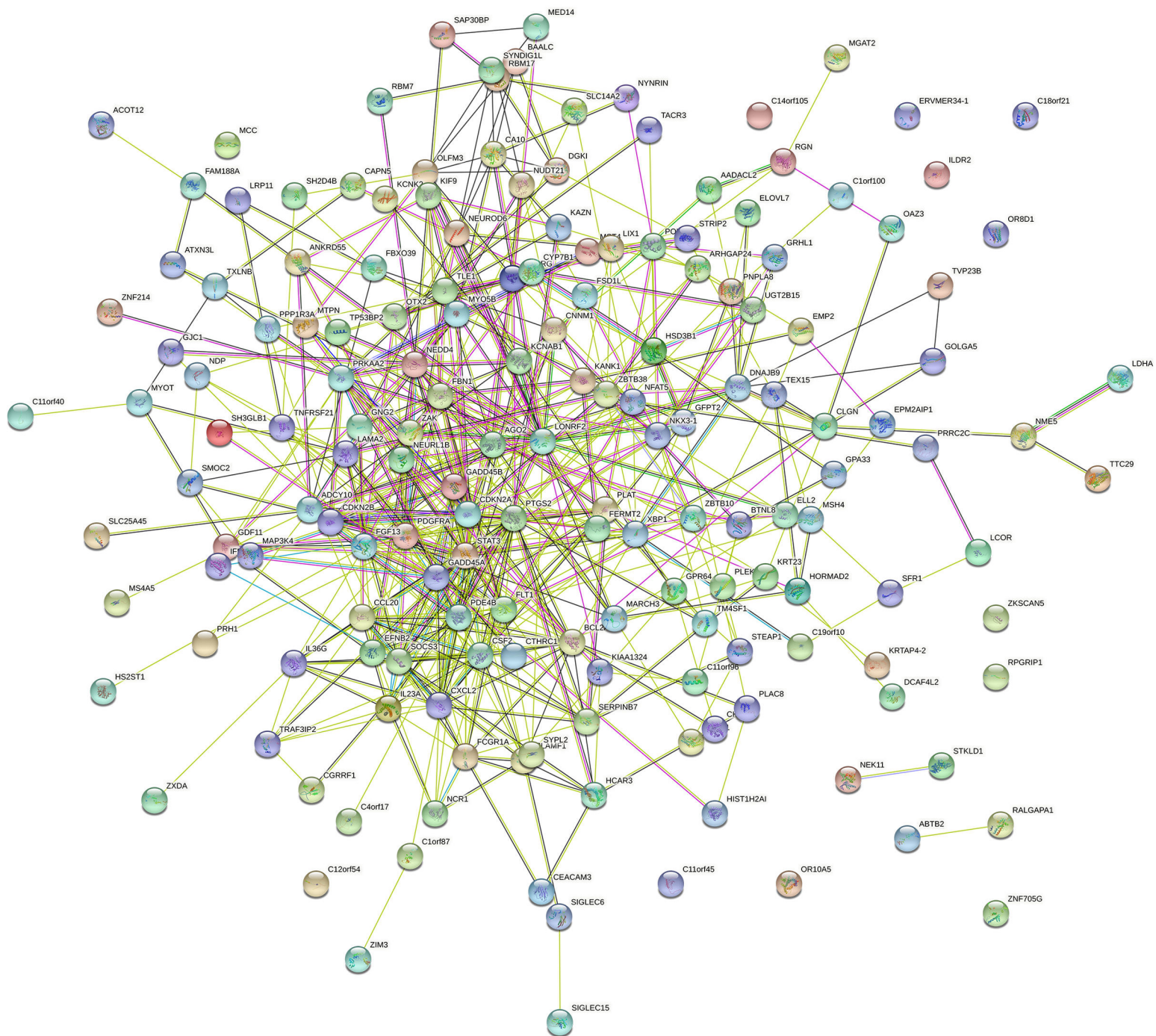

Supplement: Supplementary Materials — Supplementary Table S1. The association between clinical features and subtypes. Supplementary Table S2. Detailed information on GO and KEGG enrichment analyses of N gene sets. Supplementary Table S3. Detailed information on GO and KEGG enrichment analyses of T gene sets. Figure S1. The protein-protein interaction network for N gene sets. Figure S2. The protein-protein interaction network for T gene sets. [file 7887711.f1.zip › FigureS1.pdf]

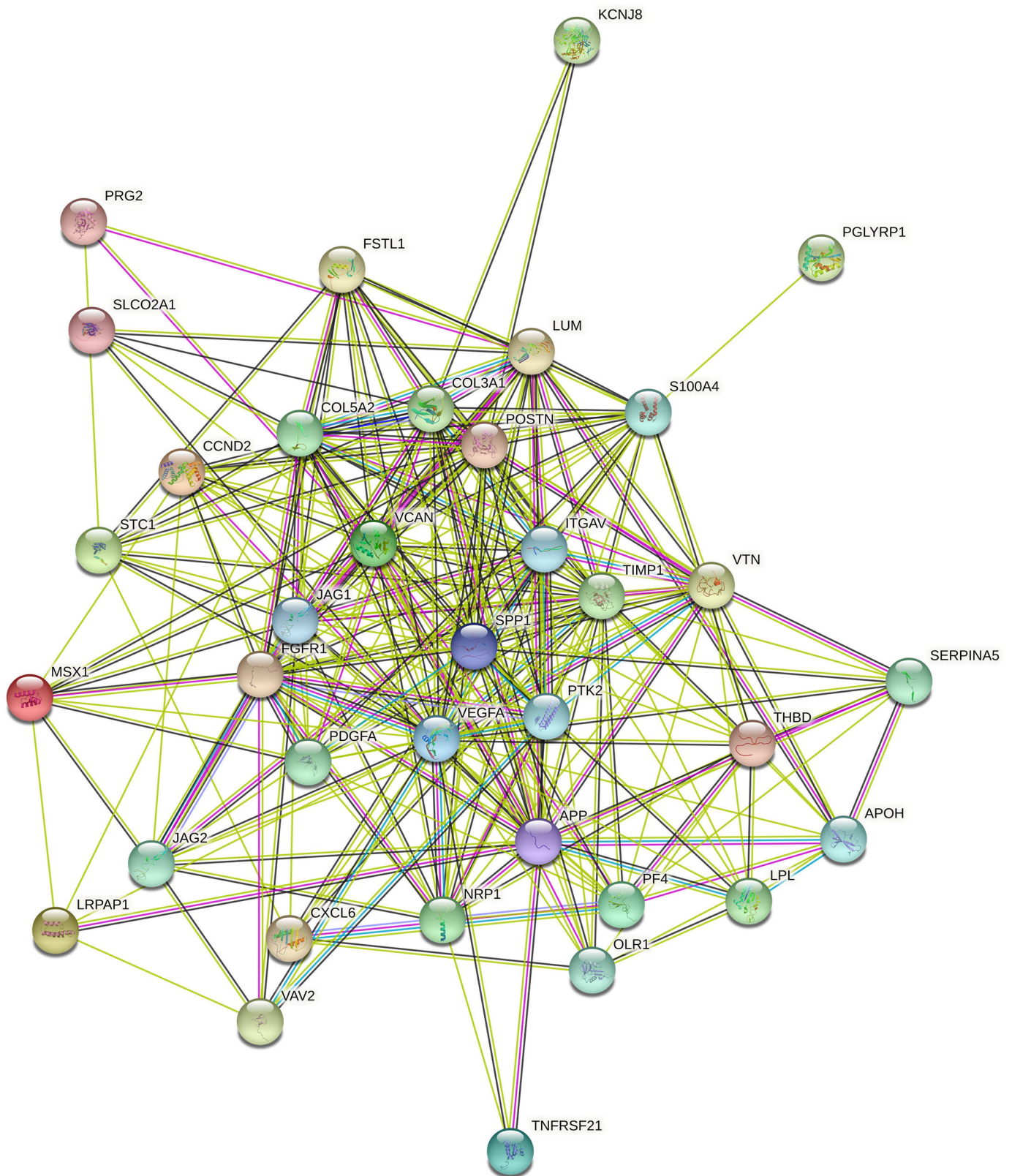

Supplement: Supplementary Materials — Supplementary Table S1. The association between clinical features and subtypes. Supplementary Table S2. Detailed information on GO and KEGG enrichment analyses of N gene sets. Supplementary Table S3. Detailed information on GO and KEGG enrichment analyses of T gene sets. Figure S1. The protein-protein interaction network for N gene sets. Figure S2. The protein-protein interaction network for T gene sets. [file 7887711.f1.zip › FigureS2.pdf]
